# UHPLC-MS Characterization and Biological Insights of Different Solvent Extracts of Two *Achillea* Species (*A. aleppica* and *A. santolinoides*) from Turkey

**DOI:** 10.3390/antiox10081180

**Published:** 2021-07-24

**Authors:** Reneta Gevrenova, Gokhan Zengin, Kouadio Ibrahime Sinan, Evren Yıldıztugay, Dimitrina Zheleva-Dimitrova, Carene Picot-Allain, Mohamad Fawzi Mahomoodally, Muhammad Imran, Stefano Dall’Acqua

**Affiliations:** 1 Department of Pharmacognosy, Faculty of Pharmacy, Medical University, 1431 Sofia, Bulgaria; rgevrenova@gmail.com (R.G.); dimizheleva@gmail.com (D.Z.-D.); 2 Department of Biology, Science Faculty, Selcuk University, Konya 42130, Turkey; sinankouadio@gmail.com; 3 Department of Biotechnology, Science Faculty, Selcuk University, Konya 42130, Turkey; eytugay@gmail.com; 4 Department of Health Sciences, Faculty of Medicine and Health Sciences, University of Mauritius, Réduit 80837, Mauritius; picotcarene@yahoo.com (C.P.-A.); f.mahomoodally@uom.ac.mu (M.F.M.); 5 Faculty of Allied Health Sciences, Institute of Diet and Nutritional Sciences, The University of Lahore, Lahore 54590, Pakistan; muhammad.imran8@dnsc.uol.eud.pk; 6 Department of Pharmaceutical and Pharmacological Sciences, University of Padova, Via Marzolo 5, 35131 Padova, Italy

**Keywords:** medicinal plants, biopharmaceuticals, hyperpigmentation, phenolics

## Abstract

In the current study, *Achillea santolinoides* and *Achillea aleppica* aeral parts and root were extracted with ethyl acetate, methanol, and water. Detailed phytochemical profiles were obtained using UHPLC-MS, yielding the identification of hydroxybenzoic and hydroxycinnamic acids, phenolic acid glycosides and sugar esters, acylquinic acids, *O*-glycosyl flavones and flavonols, and flavonoid aglycons, among others. The antioxidant properties and enzyme inhibitory activities of the extracts were assayed with in vitro tests. The phenolic content of the water extracts was significantly higher as compared to the ethyl acetate and methanol ones. *A. aleppica* aerial parts methanol extract possessed highest flavonoid content (49.18 mg rutin equivalent/g). Antioxidant properties assessment revealed that the methanol extract of *A. santolinoides* roots actively scavenged DPPH (54.11 mg TE/g) and ABTS radicals (112.53 mg TE/g) and possessed highest reducing potential (183.55 and 129.92 mg TE/g, for CUPRAC and FRAP, respectively). The ethyl acetate extracts of aerial parts and roots of both species showed highest inhibition against BuCHE (6.07–6.76 mg GALAE/g). The ethyl acetate extract of *A.* *santolinoides* aerial part showed highest inhibition against tyrosinase (73.00 mg KAE/g). These results showed that the tested *Achillea* species might represent novel phytotherapeutic avenues for the management of Alzheimer’s disease and epidermal hyperpigmentation conditions, which are both associated with oxidative stress. This paper could shed light into future potential industrial applications using the tested *Achillea* species.

## 1. Introduction

The *Achillea* genus, one of the most important genera of the Asteraceae family with ethnopharmacological significance, consists of approximately 85 species mainly distributed in Middle East regions, such as Iran, Turkey, and Serbia and Eastern regions of Europe [1]. *Achillea* species have been reported to possess highly bioactive compounds and were rich in flavones and other flavonoids [2], non-saturated carboxylic acids [3], phenolic glycosides [4], guaianolides [5], lignans [6], phthalate derivatives [7], piperidine amides, proazulenes [8], sesquiterpenes [9], sesquiterpene lactone-diol [10], sesquiterpene lactones [11], polyacetylenes [12], spirodepressolide [13], tannins [14], and triterpene alkamide [15]. An ethnobotanical survey conducted by Mohammadnoseini and colleagues (2017) highlighted the use of several *Achillea* species in traditional medicine for the management of several ailments [16]. In addition, pharmacological studies have demonstrated that various *Achillea* species possess biological activities, such as antioxidant, antibacterial, antispasmodic, and anti-inflammatory [1].

Traditionally *Achillea wilhelmsii* C. Koch (new name: *A.* *santolinoides* subsp. *wilhelmsii*) flowers powder was sprinkled on wound to promote wound healing, the decoction of the plant was used as abortifacient, against stomach pain, fever, motion of children and jaundice while teas made from young shoots were used to manage stomach disorders [17]. The use of *A. wilhelmsii* also vary according to different locations, as such, *A. wilhelmsii* is used for its antihypertensive and antihyperlipidemic properties in Iran, to treat gastrointestinal disorders in Italy, hemorrhoids in Turkey, stomachache, diabetes, gastric, and obesity in Pakistan, and detoxification, hemostasia and acesodyne in China [16]. *A. wilhelmsii* rich in flavonoids and sesquiterpene lactones, have been reported to exhibit antiproliferative and apoptotic effects in PC3 cell line by suppressing the expression of oncogene hTERT in PCa [18]; essential oil of *A. wilhelmsii* showed anxiolytic effects in rats [19]; a clinical trial conducted on 120 randomly selected men and women, aged 40–60 years, revealed that treatment with hydroalcoholic extract of *A. wilhelmsii* significantly decreased triglycerides, total cholesterol, and LDL-cholesterol levels and decreased diastolic and systolic blood pressure [18,19,20]. Although *A. aleppica* subsp. *aleppica* has been reported to be used in traditional medicine, no record of the subspecies *zederbaueri* was found. Baris and colleagues reported the antioxidant and antimicrobial activities of *A. aleppica* subsp. *zederbaueri* ethanol extract [21].

Some enzymatic activities are considered valuable targets for drugs in the management or treatment of different serious diseases. In this regard, some enzymes are great of importance in the pharmaceutical area. For example, cholinesterases are related to manage Alzheimer disease and their inhibition could increase the level of acetycholine in the synaptic cleavage and improving memory function in Alzheimer’s patients [22]. α-amylase and α-glucosidase are main enzymes in the hydrolysis of starch and the blood glucose level can be controlled with their inhibition [23]. Tyrosinase is main enzyme in the synthesis of melanin and thus its inhibition could be valuable for controlling hyperpigmentation problems [24]. In the light of these facts, the discovery of new and effective enzyme inhibitors, especially from natural sources, is gaining great interest in the scientific platform [25,26,27].

This work attempts to comparatively assess the biological activity of the different extracts obtained from *A.* *santolinoides* subsp. *wilhelmsii* and *A. aleppica* subsp. *zederbaueri* aerial parts and roots. To study differences due to extraction procedures different organic solvents were used, namely ethyl acetate and methanol operating with maceration at room temperature. Furthermore, water extracts of plant materials were obtained using boiling water as mimic of traditional preparations as infusion that use boiling water. Data obtained from the chemical investigations as well as the in vitro bioassays were then combined using multivariate data approaches to evaluate possible correlations between the observed effects and the different chemical composition of the studied extracts.

## 2. Materials and Methods

### 2.1. Plant Collection and Extract Preparation

*Achillea santolinoides* subsp. *wilhelmsii* (K. Koch) Greuter and *Achillea aleppica* subsp. *zederbaueri* (Hayek) Hub.-Mor. were collected around Konya in June 2020. The aerial parts and roots were carefully separated and then dried in a shaded and well-ventilated environment at room temperature. After drying (about 10 days), the plant materials were powdered using a laboratory mill.

The powdered plant samples were extracted by different solvents, namely ethyl acetate, methanol and water. To obtain ethyl acetate and methanol extracts, the plant samples (10 g) were macerated with 200 mL of these solvents for 24 h in room temperature. Then, the extracts were filtered and evaporated to dryness. Regarding water extracts, the plant materials (10 g) were kept in 200 mL of boiled water for 15 min, this to mimic traditional preparation that in general use hot and boiled water to prepare extraction. The water extracts were filtered and then lyophilized. Obtained extracts were stored at 4 °C until experimentation.

### 2.2. Total Phenolic and Flavonoid Content

Spectrophotometric methods were used to determine total phenolic and flavonoid content as conducted in earlier papers. Standard equivalents (gallic acid equivalent: GAE for phenolic and rutin equivalent: RE for flavonoid) were used to explain the contents in the plant extracts [28,29].

### 2.3. Ultra-High Performance Liquid Chromatography Coupled with High Resolution Mass Spectrometry (UHPLC-HRMS)

UHPLC-HRMS analysis was performed as described elsewhere (Ak et al., 2021). Briefly, the separation was carried out on a reversed phase column Waters Cortecs C18 (2.7 µm, 2.1 × 100 mm) column maintained at 40°C. The binary mobile phase consisted of 0.1% formic acid in water (A) and B: 0.1% formic acid in acetonitrile (B). The gradient program began at 5% B for one min, gradually turned to 30% B over 19 min, increased gradually to 50% B over 5min, increased gradually to 70% B over 5 min, increased gradually to 95% over 3 min and finally the system was then turned to the initial condition of 5% B, and equilibrated over 4 min. The flow rate and the injection volume were set to 300 µL/min and 1 µL, respectively. Samples were prepared as follows: methanol and aqueous extracts were dissolved in methanol-water (1:1, *v/v*) by ultrasound (20 μg/mL), while for the etylacetate extracts methanol was used preparing sample at the same concentrations. The solutions were filtered thought syringe filters 0.22 μm (Filtratech, France) and injected into chromatographic system.

Mass spectrometry analyses were carried out on a Q Exactive Plus mass spectrometer (ThermoFisher Scientific, Inc., Waltham, MA, USA) equipped with a heated electrospray ionization (HESI-II) probe (ThermoScientific, Waltham, MA, USA). The tune parameters were as follows: spray voltage −2.5 kV; sheath gas flow rate 38; auxiliary gas flow rate 12; spare gas flow rate 0; capillary temperature 320 °C; probe heater temperature 320 °C and S-lens RF level 50. Acquisition was acquired at Full scan MS and Data Dependent-MS^2^ modes. Full scan spectra over the *m/z* range 100 to 1500 were acquired in negative ionization mode at a resolution of 70,000. Other instrument parameters for Full MS mode were set as follows: automatic gain control (AGC) target 3 × 106, maximum injection time (IT) 100 ms, number of scan ranges one. For DD-MS^2^ mode, instrument parameters were as follows: microscans 1, resolution 17,500, AGC target 1 × 105, maximum IT 50 ms, MSX count 1, Top5, isolation window 2.0 *m/z*, stepped normalized collision energy (NCE) 10, 20, 60 eV. Data acquisition and processing were carried out with Xcalibur 4.0 software (ThermoScientific, Waltham, MA, USA). All chromatograms and MS/MS data for each identified compound including fragmentation patterns are given in Supplemental Materials (Figures S1–S9).

### 2.4. Determination of Antioxidant and Enzyme Inhibitory Effects

Antioxidant protocols included reducing power (cupric reducing antioxidant capacity (CUPRAC) and ferric reducing power (FRAP)), metal chelating, phosphomolybenum and free radical scavenging (2,2-diphenyl-1-picrylhydrazyl (DPPH) and 3-ethylbenzothiazoline-6-sulphonic acid (ABTS)) activities. Trolox and ethylenediaminetetraacetic acid (EDTA) were used as standards in the antioxidant assays and the results were expressed as the equivalents of these standards. Experimental details were given in our previous paper [30].

Inhibitory effects of the extracts were tested against different enzymes (tyrosinase, α-amylase, α-glucosidase and cholinesterases (AChE and BuChE). Several compounds were used as standards (galatamine for cholinesterases; kojic acid for tyrosinase; acarbose for α- amylase and α-glucosidase) and the results were expressed as the equivalents of these standards. The enzyme inhibitory assays were performed as done in our earlier paper [31].

### 2.5. Statistical Analysis

Relative quantitative data of extracts molecules obtained from UHPLC-MS analysis was submitted to principal component analysis and Clustered Image Maps successively, for viewing the differential expression of molecules among extracts. Afterward, for biological, One-way ANOVA following by Tukey’s test were performed to determine any differences between the extracts of each studied species. *p* < 0.05 were assigned to be statistically significant. Then, for comparison both species extracts biological activities, principal component analysis (PCA) and Clustered Image Maps was subsequently achieved. For both realized Clustered Image Maps, “Wards” and “Euclidean” were use as linkage rule and similarity measure, respectively. The relationship between metabolites and biological activities was investigated using partial least squared regression analysis. The goodness of the model was measured through the estimation of the cumulative modeled variation in the metabolite matrix R^2^X(cum) and the cumulative modeled variation in the biological activities matrix R^2^Y(cum). All statistical procedures were performed using R software v. 3.6.1.

### 2.6. Bioinformatics Analysis

To investigate the genes targeted by the sesquiterpene lactones and derivatives and some phenolic compounds, the datasets for mRNA of DIGEP-Pred web-sever [32] was employed. The compounds were artabsin, dehydroleucodin, dihydrosantamarin, leucodin, matricin, tanaparthin peroxide, neochlorogenic acid, chlorogenic acid, homoorientin, vitexin and isovitexin. Only the genes with Pa (probability “to be active”) higher than 0.5 were retained. Then for KEGG pathway analysis, the obtained up-regulated and down-regulated mRNA data were submitted to Enrichr websever [33].

## 3. Results and Discussion

### 3.1. Chemical Profile

After the qualitative screening of metabolites profiles in the different extracts of the species, unsupervised principal component analysis (PCA) was carried out on the relative intensities of metabolites peak area obtained through UHPLC-MS analysis in order to screen the molecules variation between both species’ samples. Before PCA processing, metabolites profiles were log transformed and autoscaled to ensure an equal contribution of variables in prediction outcomes. From the extracted principal components (PCs), only the first six showed eigenvalue above one. In addition, they displayed a cumulative proportion explained variance higher than 80%, therefore there were used as recommended by Kaiser [34]. The molecules strongly associated with each of them were summarized in Table S1. Overall, 18, 9, 5, 5, 10, and eight molecules had the highest contribution scores on PC1, PC2, PC3, PC4, PC5, and PC6, respectively.

**Figure 1:**
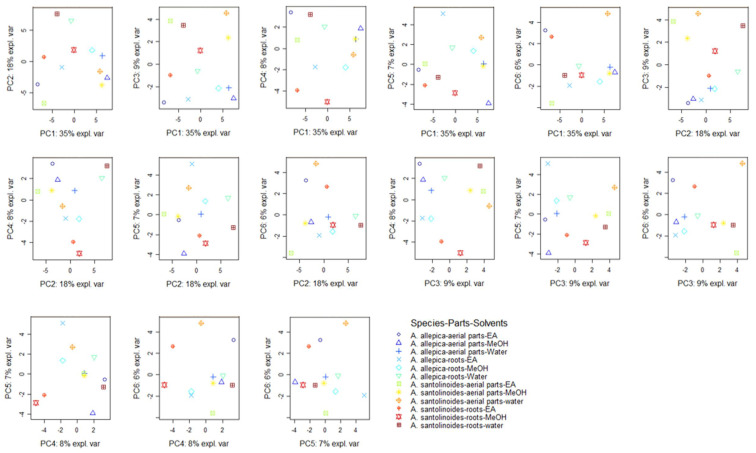
Score plots of principal component analysis on the relative quantitative metabolites data of *Achillea* species.

**Figure 2:**
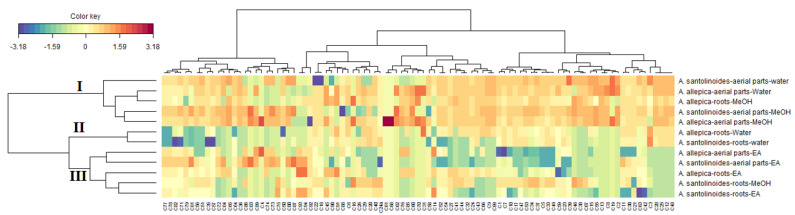
Clustered Image Map on the relative quantitative metabolites data obtained through UHPLC-MS analysis (Red color: High concentration, Blue color: low concentration). (C1) protocatechuic acid-O-hexoside, (C2) caffeoylgluconic acid, (C3) protocatechuic acid, (C4) p-hydroxyphenylacetic acid-O-hexoside, (C5) protocatechuic acid-O-hexoside isomer, (C6) syringic acid 4-O-hexoside (C7) neochlorogenic (3-caffeoylquinic) acid, (C8) caffeoylgluconic acid isomer, (C9) caffeic acid-O-hexoside, (C10) gentisic acid-O-hexoside, (C11) vanillic acid 4-O-hexoside, (C12) caffeoylgluconic acid isomer, (C13) O-caffeoyl hexose isomer, (C14) 4-hydroxybenzoic acid, (C15) 4-hydroxybenzoic acid-hexoside, (C16) p-hydroxyphenylacetic acid O-hexoside, (C17) quinic acid, (C18) chlorogenic (5-caffeoylquinic) acid, (C19) p-coumaric acid, (C20) 3-feruloylquinic acid, (C21) p-hydroxyphenylacetic acid, (C22) caffeic acid, (C23) gentisic acid, (C24) 5-p-coumaroylquinic acid, (C24a) 1,3-dicaffeoylquinic acid, (C25) caffeic acid-O-hexoside isomer, (C26) 5-feruloylquinic acid, (C27) m-coumaric acid, (C28) 5-p-coumaroylquinic acid isomer, (C29) 4-feruloylquinic acid, (C30) vanillic acid, (C31) o-coumaric acid, (C32) vanillic acid-4-O-(6-O-caffeoyl)-hexoside, (C33) 3,4-dicaffeoylquinic acid, (C34) 1,5-dicaffeoylquinic acid, (C35) 3,5-dicaffeoylquinic acid, (C36) dicaffeoyl-tetrahydroxy-pentanoic acid, (C37) 4,5-dicaffeoylquinic acid, (C38) shikimic acid, (C39) salicylic acid, (C40) 3-feruloyl-4-caffeoylquinic acid, (C41) 3-p-coumaroyl-5-caffeoylquinic acid, (C42) caffeic acid-O-(salicyl)-hexoside, (C43) 3-feruloyl-5-caffeoylquinic acid, (C44) 4-p-coumaroyl-5-caffeoylquinic acid, (C45) 1-caffeoyl-3-feruloylquinic acid, (C46) 3,4,5-tricaffeoylquinic acid, (C47) 6, 8-diC-hexosidyl-luteolin, (C48) O,C-dihexosyl-luteolin, (C49) diC-hexosyl-apigenin, (C50) 6-C-hexosyl-8-C-pentosyl-luteolin, (C51) 2″-O-pentosyl-6-C-hexosyl-luteolin, (C52) homoorientin, (C53) 6-C-hexosyl-8-C-pentosyl apigenin, (C54) orientin (luteolin-8-C-glucoside), (C55) C-hexosyl-C-pentosyl methylluteolin, (C56) rutin, (C57) vitexin, (C58) isovitexin, (C59) 2″-O-pentosyl-6-C-hexosyl-methylluteolin, (C60) Luteolin-7-O-glucosidea, (C61) chrysoeriol-6-C-hexoside, (C62) nepetin-O-hexuronide, (C63) 6-methoxykaempferol-O-hexoside, (C64) nepetin-O-hexoside, (C65) kaempferol-3-O-glucoside, (C66) isorhamnetin 3-O-glucoside, (C67) apigenin-7-O-glucoside, (C68) cirsiliol-O-hexoside, (C69) chrysoeriol-O-hexuronide, (C70) jaceosidin-O-hexuronide, (C71) luteolin, (C72) quercetin, (C73) patuletin (6-methoxyquercetin), (C74) axillarin, (C75) apigenin, (C76) kaempferol, (C77) hispidulin (scutellarein-6-methyl ether), (C78) chrysoeriol, (C79) cirsiliol, (C80) quercetagetin-3,6,3′(4′)-trimethyl ether, (C81) cirsimaritin (6-hydroxyapigenin-6,7-dimethyl ether), (C82) santin/eupatilin, (C83) acacetin, (C84) tanaparthin-peroxide, (C85) achillicin/matricin, (C86) dehydroachillin/dehydroleucodin, (C87) achillin/leucodin, (C88) artabsin, (C89) dihydrosantamarin, (C90) tetradecenoic acid amide, (C91) linolenamide, (C92) linoleamide, (C93) palmitamide, (C94) oleamide.

Afterwards, looking at the different score plot displayed in Figure 1, a considerable difference between the samples was observed. On the other hand, despite some samples seemed have common characteristics, it was difficult to clearly identify the different samples. For this purpose, an additional analysis i.e., Clustered Image Maps was performed from the coordinates of the samples derived from PCA. The samples can be split into three main clusters, the cluster I and III comprised on five samples respectively and the cluster II was represented by two samples (Figure 2). Of these three clusters, the samples of the clusters I were remarkably rich in several molecules. Hence most of the molecules were occurred predominantly in the methanol and water extracts obtained from both species the aerial parts as well as the methanol extract of *A. aleppica* subsp. *zederbaueri* roots. This finding reflects the polar character of the molecules present in these two species.

The total phenolic and flavonoid contents were determined using Folin Ciocalteau and aluminum chloride colorimetric methods, respectively. In *A. alleppica* extracts, water extract of root possessed the highest level of total phenolic (43.24 mg GAE/g), while the methanol extract of root contained the highest amounts of total phenolic (52.07 mg GAE/g) in *A. santolinoides* extracts. On the other hand, methanol extract of *A. aleppica* aerial part and ethyl acetate extract of *A. santolinoides* aerial part were found to have the highest flavonoid content respectively (49.18 and 19.58 mg RE/g) (Table 1).

**Figure 3:**
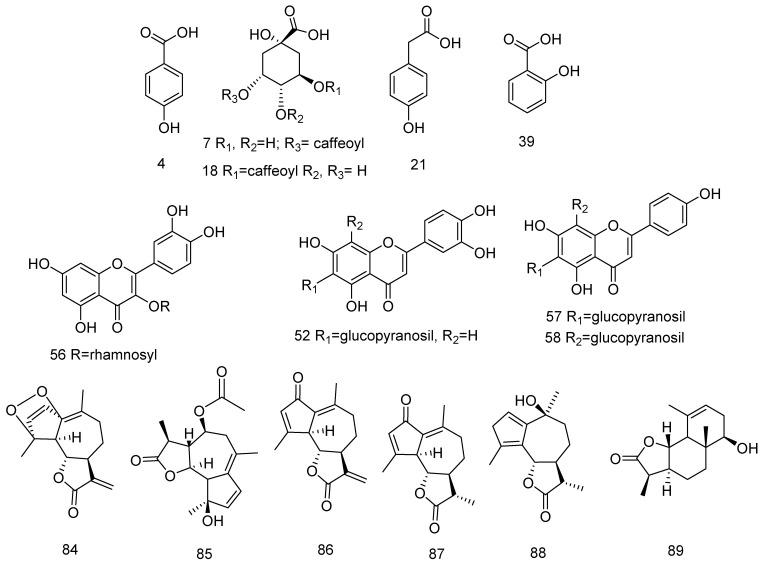
The main components in the tested *Achillea* extracts (for the compound numbers see Table 2).

To identify the metabolites present in the studied extracts, non-targeted profiling was performed by ultra-high-performance liquid chromatography-quadrupol-Orbitrap high resolution mass spectrometry (UHPLC-HRMS). Under the conditions of Full scan-ddMS^2^/Top 5, the mass range for survey full scan was set at *m/z* 100–1200 and the MS/MS analyses were acquired by stepped higher energy collision-induced dissociation (hcd) at 10, 20, and 60 eV for data dependent (dd) MS^2^scans. The key points in the compounds annotation/dereplication were the accurate masses in Full MS and ddMS^2^, MS/MS fragmentation patterns, relative abundance of the precursor and fragment ions, elemental composition, matching with the simulated monoisotopic peak profiles, and consistence with the retentions times and fragmentation spectra of reference standards and literature data [35,36,37]. The chemical structures of main components are depicted in Figure 3.

A variety of metabolites were identified and tentatively elucidated in the assayed extracts, including, 14 hydroxybenzoic and hydroxycinnamic acids together with 12 phenolic acid glycosides and sugar esters, 18 acylquinic acids, 11 *C*-glycosyl flavones, 2 *C*, *O*-glycosyl flavones, 11 *O*-glycosyl flavones and flavonols, and 12 flavonoid aglycons, six sesquiterpene lactons, and five fatty acid amides (Table 2, Figure S1–S4). All compounds are reported for the first time in the studied *Achillea* sp.

#### 3.1.1. Hydroxybenzoic, Hydroxycinnamic and their Glycosides, and Sugar Esters

Based on the fragmentation patterns and retention times of reference standards, five hydroxybenzoic acids (3, 14, 23, 30, 39) and four hydroxycinnamic acids (19, 22, 27, 31) together with *p*-hydroxyphenylacetic acid (21) were identified in the extracts (Table 2, Figure S1). In addition, 7 hydroxybenzoic and hydroxycinnamic acids hexosides (1, 4–6, 9–11, 15, 16, 25) together with a sugar ester *O*-caffeoyl-hexose (13) were tentatively elucidated (Ak et al., 2021). MS/MS spectra of 3 caffeoylgluconic isomers 2, 8, and 12 ([M-H]^−^ at *m/z* 357.084) were obtained (Table 2, Figure S1). They yielded a base peak at *m/z* 195.050 (C_6_H_11_O_7_^−^) corresponding to the [gluconic acid-H]^−^ supported by the fragment ions at *m/z* 177.040 [GA-H-H_2_O]^−^, 87.007 [GA-H-C_3_H_8_O_4_]^−^ and 59.012 [GA-H-C_3_H_8_O_4_-CO]^−^ (Table 2).

Vanillic acid-4-*O*-(6-caffeoyl)-hexoside (32) was deduced from the loss of vanillic acid (168 Da) at *m/z* 323.077 and a subsequent transition 323.077→221.046 [M-H-102]^−^ arising from the hexose cross ring cleavage (^0,4^X). The latter ion points out to the caffeoyl moiety at Hex C-6. Regarding 42, the prominent ion at *m/z* 323.077 [M-H-C_7_H_6_O_3_]^−^ and a base peak at *m/z* 137.023 [salicylic acid-H]^−^ together with *m/z* 93.033 [salicylic acid-H-CO_2_]^−^ were in accordance with caffeic acid-*O*-(salicyl)-hexoside (Table 2). Both 32 and 42 were annotated in *Tanacetum vulgare* [35].

Among the compounds of the group, phenolic acid-hexosides 5, 15, and 16 were the major compounds in the aerial parts of both species (Figure S1A,B), especially protocatechuic acid- and 4-hydroxybenzoic acid-hexoside in *A. santolinoides*. Syringic acid-hexoside (6) was presented mainly in *A. allepica* roots (Table 2, Figure S1D). In addition, quinic acid was commonly found in all samples.

#### 3.1.2. Acylquinic Acids

Overall, 7 *mono*AQA, 10 *di*AQA and 1 *tri*AQA were identified in the studied extracts, mostly in the methanol and water extracts (Table 2, Figure S2). Their recognition was based on the fragmentation patterns and diagnostic ions for different subclasses AQA reported elsewhere [35,36]. Thus, 18, 24, 26, and 28 were assigned to 5-AQA as suggested a base peak at *m/z* 191.055 [quinic acid-H]^−^, while 7 and 20 were identified as 3-AQA.

Five peaks 24, 33–35, and 37 ([M-H]^−^ at *m/z* 515.119) afforded prominent ions at *m/z* 353.088 and 191.055 indicating the subsequent losses of a caffeoyl moiety (Table 2). The vicinal *di*CQA 33 and 37 were witnessed by the “dehydrated” ion of quinic acid at *m/z* 173.044 (100%) supported by the diagnostic ions at *m/z* 335.0771 [CQA-H-H_2_O]^−^ and 135.044 [caffeic acid-H-CO_2_]^−^ in 3,4-*di*CQA (33) (Table 2). The second isomer was assigned to 4,5-*di*CQA as suggested by the lack of ion at *m/z* 335 and the chromatographic behavior on the reverse phase (the most lipophilic *di*CQA isomer). The base peak at *m/z* 191.055 evidenced 1,3-*di*CQA (24a), 1,5-*di*CQA (34) and 3,5-*di*CQA (35) supported by the relative abundance of the ions at *m/z* 179.034 and *m/z* 135.044: 73.2% and 58.7% (24a), 6.2% and 6.6% (34), and 53.1% and 52.7% (35), respectively.

Two *p*-coumaroyl-caffeoylquinic acids (*p*-CoCQA) isomers 41 and 44 at *m/z* 499.122 (C_25_H_23_O_11_) were deduced from the distinctive fragments at *m/z* 337.093 [M-H-caffeoyl]^−^, *m/z* 163.039 [*p*-CoA-H]^−^ and *m/z* 119.049 [*p*-CoA-H-CO_2_]^−^ for *p*-coumaric acid (Table 2). Compound 41 afforded an abundant ion *m/z* 337.093 (83.6%) indicating a loss of caffeoyl residue before the *p*-coumaroyl one. This assignment was also supported by the base peak at *m/z* 163.039 as was registered in 3-*p*-CoQA [35]. Thus, 41 was identified as 3-*p*-Co-5CQA, while *vic* 4-*p*-Co-5-CQA was supported by the abundant ions at *m/z* 337.093 (61.7%) and 173.044 (100%).

Three peaks 40, 43, and 45 yielded a precursor ion at *m/z* 529.136 (C_26_H_25_O_12_) along with prominent fragments at *m/z* 367.103 [M-H-caffeoyl]^−^ and *m/z* 353.270 [M-H-feruloyl]^−^ for feruloyl-caffeolylquinic acids (FCQA). The fragment ion at *m/z* 335.0754 [M-H-FA]^−^ accompanied by the “dehydrated” form of quinic acid suggested 3F-4CQA (40) [36]. The assignment of 3F-5CQA was witnessed by the base peak at *m/z* 193.050 together with the abundant ion at *m/z* 134.036 (74.4%) as was registered in 3-FQA (Table 2). 1C-3FQA (45) was discernible by the base peak at *m/z* 161.023 [CA-H-H_2_O]^−^ accompanied by the abundant ions at *m/z* 179.034 [CA-H]^−^ (42.3%) and 367.104 (34.1%) [36]. The MS/MS spectrum of 46 was consistent with 3,4,5-*tri*CQA [35].

Clorogenic acid (18) was the main *mono*AQA in the aerial parts and roots of both *Achillea* sp. *di*CQA were dominated by 3,5-*di*CQA (35) (Figure S2) except for *A. santolinoides* roots where 1,3-*di*CQA (24a) was a major compound of the group (Figure S2C).

#### 3.1.3. Flavonoids

##### C-, C,O- and O-Flavonoid Glycosides

MS/MS spectra of the *C*-glycosyl flavones 52, 54, 57, and 58 were acquired (Table 2, Figure S3). In the (−) ESI mode 54 and 57 yielded a base peak ^0,2^X^−^ [(M-H)-120]^−^ at *m/z* 327.051 (54) and 311.056 (57) supported by the relevant ions at *m/z* 299.056 ^0,2^X/CO^−^ [(M-H)-120–28]^−^ and *m/z* 283.061, respectively. This fragmentation pathway was consistent with *C*-8 hexosyl luteolin/apigenin [38]. In contrast, corresponding *C*-6 hexosyl isomers 52 and 58 was shown by the ions at *m/z* 447.094 [M-H]^−^ (100%) and 431.099, as well as ^0,3^X^−^ at *m/z* 357.062 and 341.067, and ^0,2^X^−^ at *m/z* 327.051 and 311.056. The aglycones luteolin (52, 54) and apigenin (57, 58) were discernable by the RDA ions ^1,3^A^−^ (*m/z* 151.022), ^0,4^A^−^ (*m/z* 107.012), ^1,3^B^−^ at *m/z* 133.028 (52, 54) and 117.033 (57, 58). Based on the comparison with reference standards, compounds 52, 54, 57, and 58 were identified as homoorientin, orientin, vitexin, and isovitexin, respectively.

Three isobars species 49, 55, and 59 shared the same [M-H]^−^ at *m/z* 593.152 (Table 2, Figure S3). Concerning 49, typical ions of the *C*-glycosyl flavon pathway were produced at *m/z* 473.109 [(M-H)-120]^−^, 383.077 [(M-H)-90–120]^−^ and 353.067 [(M-H)-2 × 120]^−^ suggesting the presence of two *C*-hexosyl moieties on the flavonoid skeleton [35]. Considering that the *C* glycosylation appears exclusively at C-6 and 8 of flavones, compound 47 was assigned as 6, 8-di*C*-hexosyl-apigenin. *C*-hexosyl-*C*-pentosyl methylluteolin (55) was discernible by the prominent ions [^0,3^X_0_/^0,2^X_1_]^−^ at *m/z* 413.088 [(M-H)-60–120]^−^ and [^0,1^X_0_/^0,1^X_1_]^−^ at 323.057 [(M-H)-120-150]^−^ suggesting the presence of both *C*-pentosyl (X_0_) and *C*-hexosyl (X_1_) moieties. Additionally, methylluteolin was assigned on the basis of specie at *m/z* 299.560 [MeLu-H]^−^ and 298.048 Y_0_/^0,2^X_1_/•CH_3_/CO [38]. On the other hand, compound 59 yielded prominent ions at *m/z* 323.057 ([(M-H)-(132 + H_2_O)-120]^−^ and 443.097 ([(M-H)-(132 + H_2_O)]^−^ suggesting *O*-pentosyl unit at 2″ of the primary hexose [38,39]. Diagnostic ions at *m/z* 308.032 (Z_1_^−^/^0,2^X_0_/•CH_3_) and 298.049 (Y_1_^−^/^0,2^X_0_/•CH_3_/CO) allowed for the annotation of methylluteolin. Thus, compound 59 was identified as 2″-*O*-pentosyl-6-*C*-hexosyl-methylluteolin.

Among the isobar species indicted as 47, 48, and 56 with [M-H]^−^ at 609.147, 47 was annotated as 6, 8-di*C*-hexosyl-luteolin, while 48 was assigned to *O*, *C*-dihexosyl-luteolin. The latter structure was shown by a series of diagnostic ions at *m/z* 447.093 [M-H-Hex]^−^_,_ 357.062 [M-H-Hex-90]^−^ and 327.051 [M-H-Hex-120]^−^. Additionally, ions at *m/z* 298.048 (Y_1_^−^/^0,2^X_0_/CHO•), 175.039 (^1,3^A^−^/H_2_O^−^) and 133.028 ^1,3^A^−^ indicated luteolin. The sugar chain of 56 was consistent with rutinose (308 Da); aglycone quercetin was witnessed by a series of fragments including RDA ions at *m/z* 178.998 [^1,2^A-H]^−^, 163.003 [^0,2^A-H]^−^, 151.002 [^1,3^A]^−^, 121.028 [^1,2^B]^−^, 107.012 [^0,4^A]^−^. Based on comparison with reference standard, 56 (rutin), 60 (luteolin-7-glucoside), 65 (kaempferol-3-glucoside), 66 (isorhamnetin-3-glucoside), 67 (apigenin-7-glucoside), luteolin (50), quercetin (72), apigenin (75), kaempferol (77) and chrysoeriol (78) were unambiguously identified (Table 2).

Compounds 62, 69, and 70 presented similar fragmentation patterns yielding base peaks at *m/z* 315.051 (61), 299.056 (69, 70) and 329.067 (71) [(M-H)-HexA]^−^, respectively, indicating flavonoid hexuronides (Table 2).

Nepetin-*O*-hexuronide (62) was deduced from the fragment ions at *m/z* 243.030 [(M-H)-HexA-CH_3_-HCO•-CO]^−^, 227.035 [(M-H)-HexA-CH_3_-HCO•-CO_2_]^−^ as well as RDA ions at *m/z* 133.028 (^1,3^B^−^). Compound 69 was ascribed to chrysoeriol-*O*-hexuronide (^1,3^A^−^ at *m/z* 151.002, ^0,4^A^−^ at *m/z* 107.013), while 70 was consistent with jaceosidin-*O*-hexuronide [40]. It should be noted that in both *Achillea* species the predominant compounds among the flavonoid glycosides were *C*-glycosyl flavons homoorientin (52) and vitexin (57) together with *C*-pentosyl-*C*-hexosyl-apigenin/methylluteolin (53, 55) (Table 2, Figure 3). Despite the similarity of the composition, compound 57 was mostly produced by the *A. wilhemsii* aerial parts.

##### 6-Methoxyflavonoids

6-Methoxyflavonoids annotation was based on the characteristic fragment ions delineated in the previous studies on *Tanacetum* sp. [35,36].

**Figure 4:**
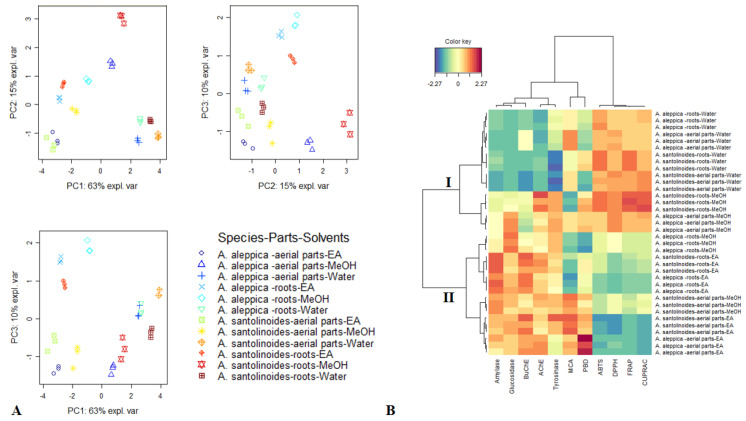
Exploratory multivariate analysis on biological activities of *Achillea* species. (**A**) Score plots of principal component analysis; (**B**) Clustered Image Map (**Red** color: High activity, **Blue** color: low activity.

Compound 79 ([M-H]^−^ at *m/z* 329.067 (C_17_H_14_O_7_) could be used to illustrate the fragmentation pattern of 6-methoxylated flavones (Table 2, Figure 4). In (−) ESI-MS/MS 79 yielded fragment ions at *m/z* 314.043 [M-H-•CH_3_]^−^, 299.016 [M-H-2•CH_3_]^−^, 271.025 [M-H-2•CH_3_-CO]^−^, 255.029 [M-H-2•CH_3_-CO_2_]^−^, 243.029 [M-H-2•CH_3_-2CO]^−^, 230.147 [M-H-2•CH_3_-2CO-CHO•]^−^ and 227.034 [M-H-2•CH_3_-CO-CO_2_]^−^. Consistent with the Orbitrap-based approach for the recognition of methoxylated flavonoids, RDA ions were registered at *m/z* 163.002 (^1,3^A^−^-H_2_O-CH_2_), 136.987 (^1,3^A^−^-CO-2•CH_3_), and 135.007 (^1,3^A^−^-H_2_O-CO-CH_2_) [35,36]. On the other hand, ^1,3^B^−^ at *m/z* 133.028 indicated two hydroxyl groups in the ring B. Thus, compound 79 was assigned as 6-hydroxyluteolin-6, 7-dimethyl ether (cirsiliol), previously reported in *Achillea* sp. [41].

In addition, quercetagetin-3, 6-dimethyl ether (axillarin) (74) was deduced from the typical losses from RDA ion (^1,3^A^−^) at *m/z* 165.990 (^1,3^A^−^-•CH_3_), 139.039 (^1,3^A^−^-CO-CH_2_), 136.986 (^1,3^A^−^-CO-CH_4_) and ^1,2^ B^−^ at *m/z* 121.028. Within this group, compounds 73 (patuletin), 74 (axillarin), and quercetagetin-3,6,3′(4′)-trimethyl ether (80) were quercetagetin derivatives, while compounds 77 (hispidulin) and 81 (cirsimaritin) were scutellarein derivatives (Table 2, Figure S4). In the (−) ESI mode compound 82 gave consequent losses of 3 methyl radicals at *m/z* 328.059, 313.036 and 298.012. Despite the lack of the initial RDA, a series of low abundant ^1,3^A^−^ ions were generated at *m/z* 165.990 (^1,3^A^−^-•CH_3_), 164.981 (^1,3^A^—^CH_4_), 163.002 (^1,3^A^−^-H_2_O), 136.987 (^1,3^A^−^-CO-CH_4_). Moreover, (^1,3^B^−^-•CH_3_-CH_2_) at *m/z* 132.020 indicated 2 methoxy groups either in C-3, C-4′ or C-3′, C-4′, as was observed in santin and eupatilin, respectively [35,42].

Overall, flavonoid aglycones fingerprints of both *A. allepica* and *A. santolinoides* aerial parts extracts were dominated by cirsimaritin (81) and santin/eupatilin (82) (Figure S4).

##### Sesquiterpene Lactones (STLs)

The dereplication of STLs was based on the fragmentation patterns and diagnostic ions in positive ion mode as more informative for this class of natural compounds [36,43]. Based on accurate masse in Full MS, MS/MS fragmentation patterns, relative abundance of precursor and fragment ions, and elemental composition, 6 STLs were tentatively annotated in *Achillea* extracts.

MS/MS spectrum of 84 [M + H]^+^ at *m/z* 279.1226, yielded a fragment ions at m/z 261.111 [M + H-H_2_O]^+^ and 243.101 [M + H-2H_2_O]^+^ and a base peak at m/z 237.111 [M + H-H_2_O-CH_2_]^+^. This fragmentation pathway could be associated with the presence of peroxide group and 84 was tentatively ascribed to tanaparthin-peroxide, previously isolated from *Achillea nobilis* (Table 2) [44]. Compound 85 [M + H]^+^ at *m/z* 307.153 gave a base peak at *m/z* 247.132 [M + H-CH_3_COOH]^+^ which is in accordance with the structure of achillicin/matricin. Compound 87 differs from 85 for 60 Da (CH_3_COOH) and revealed the same fragmentation patterns as 85. Thus, compound 87 was tentatively annotated as achillin/leucodin (Table 2). Similarly, 86 [M + H]^+^ at *m/z* 245.117 was related to dehydroachillin/dehydroleucodin. Based on MS/MS fragmentation pathway, including characteristic ions corresponded to the loss of H_2_O (−18 Da), 2xH_2_O (−36 Da), CO (−28 Da), as well as concomitant loss of H_2_O + CO (−46 Da), 2H_2_O + CO (−64 Da), 88 and 89 were ascribed to artabsin and dihydrosantamarin, respectively, and were previously isolated from *Achillea collina* [45].

##### Fatty Acids Amides

The peak at 92 afforded a precursor ion at *m/z* 280.263 (C_18_H_33_NO) together with distinctive fragments at *m/z* 263.236 [M + H-NH_3_]^+^ and *m/z* 245.225 [M + H-NH_3_-H_2_O]^+^, suggesting amide of octadecadienoic acid. Additionally, the suggested structure was supported by the fragments at *m/z* 81.070 (C_6_H_9_), 69.070 (C_5_H_9_), 57.070 (C_6_H_9_) (Table 2). Thus, 92 was assigned as linoleamide [46]. Similarly, 90, 91*,* 93, and 94 were related to tetradecenoic acid amide, linolenamide, palmitamide and oleamide, respectively (Table 2) [46].

### 3.2. Antioxidant Effects

The total antioxidant capacity of the extracts was determined using the phosphomolybdenum assay. As shown in Table 1, for both species, the aerial part ethyl acetate extracts (2.33 and 1.95 mmol TE/g) showed the highest activity. Further antioxidant assays, free radical scavenging (DPPH and ABTS), reducing power (FRAP and CUPRAC), and metal chelating were conducted in order to obtain a comprehensive understanding of the antioxidant potential of the extracts and results were presented in Table 3. The ability of the extracts to scavenge free radicals was summarized in Table 3. Methanol extracts of *A. aleppica* aerial parts (55.15 mg TE/g) and *A. santolinoides* roots (54.11 mg TE/g) showed highest scavenging activity against DPPH. In contrast *A. aleppica* roots water extract (101.88 mg TE/g) and *A. santolinoides* roots methanol extract (112.53 mg TE/g) were most potent in scavenging ABTS. Protocatechuic acid and its derivatives identified in the A*. aleppica* roots water extract, *A. aleppica* aerial parts methanol extract, and *A. santolinoides* roots methanol extract, has been reported to exhibit radical scavenging activity [47,48]. Neochlorogenic (3-caffeoylquinic) acid also identified in these extracts was previously reported to exhibit scavenging activity against DPPH [49]. The reducing capacity of the extracts to donate electron and thus act as reducing agents is commonly assessed using two widely used methods, namely FRAP (ferric ion) and CUPRAC (cupric ion) assays. Similar to the DPPH assay, methanol extracts of *A. aleppica* aerial parts and *A. santolinoides* roots showed highest reducing capabilities (Table 3). The chelating capacity of the extracts was also evaluated. The water extract of the aerial parts of *A. aleppica* (25.37 mg EDTAE/g) and ethyl acetate and water extract of the aerial parts of *A. santolinoides* (27.37 and 26.06 mg EDTAE/g), respectively possessed strong chelating ability. Caffeic acid, chlorogenic acid, and protocatechuic acid were identified in aerial parts of A. aleppica water and A. santolinoides ethyl acetate extracts. Interestingly, a study conducted by Andjelković, et al. [50] has assessed the metal chelating potential of these phenolic compounds and reported that caffeic acid and chlorogenic acid were the strongest metal chelators. It can also be suggested that the presence of these metal chelators created a synergistic effect, therefore enhancing the metal chelating properties of these extracts. The hydroalcoholic extract of *A.* *santolinoides* was previously reported to possess antioxidant effect on brain tissues in pentylenetetrazole-induced seizures Wistar rat models [51]. The essential oil of *A.* *santolinoides* was also found to exhibit antioxidant potential against DPPH radical (IC_50_ = 129–372 mg/mL) [52].

### 3.3. Enzyme Inhibitory Effects

The inhibitory ability of extracts prepared from the aerial parts and roots of the selected *Achillea* species against enzymes targeted in the management of diabetes mellitus type II, Alzheimer’s disease, and skin hyperpigmentation problems was investigated. Alzheimer’s disease has escalated to epidemic proportions and the need for complementary therapeutic agents to effectively manage this debilitating condition is of paramount importance. From Table 4, *A. aleppica* aerial parts ethyl acetate extract and *A. santolinoides* roots methanol exhibited highest inhibition against AChE. A previous molecular docking study confirmed the interaction of orientin with AChE which showed least binding energy and highest binding affinity [53]. Vitexin also identified in these extracts was previously reported to bind effectively with AChE through strong hydrogen bonding [54]. Acacetin was previously reported to exhibit moderate to potential AChE inhibitory properties [55]. However, in the present study, acacetin was not identified in extracts showing more potent inhibitory activity against AChE. Santin/eupatilin identified in the ethyl acetate extracts of *A. aleppica* roots and *A. santolinoides* aerial parts was previously reported to inhibit BuChE in an in silico study. On the other hand, the ethyl acetate extracts of *A. aleppica* aerial parts and roots (6.07 and 6.73 mg GALAE/g) and as well as that of *A. santolinoides* aerial parts (6.76 and 6.70 mg GALAE/g) were most active against BuChE. The inhibition of BuChE has been advocated in the later stage of Alzheimer’s disease. During the progression of the disease, BuChE level increases, exacerbating the conditions of the patient [56]. The ability of the extracts to inhibit enzymes targeted in the management of diabetes type II, namely α-amylase and α-glucosidase, was presented in Table 4. A low inhibition against both enzymes was noted, suggesting that the different extracts of *A. aleppica* and *A. santolinoides* aerial parts and roots possessed weak anti-diabetic properties. Tyrosinase, a rate limiting enzyme responsible for the biosynthesis of melanin, is considered to be a key therapeutic strategy for the management of skin hyperpigmentation conditions. In the present study, methanol extracts of *A. aleppica* aerial parts and roots showed the highest inhibitory activity against tyrosinase. In other side, ethyl acetate and methanol extracts of both studied parts of *A. santolinoides* displayed strongest anti-tyrosinase activity. Hispidulin, isolated from *Phyla nodiflora* and identified in extracts which actively inhibited tyrosinase was previously reported to exhibit inhibitory action against tyrosinase with an IC_50_ value of 146 µM [57].

### 3.4. Data Mining

Subsequent to comparison of the bioactivities of the samples of each species, principal component analysis (PCA) was used in order to uncover the similarities/differences among the extracts of both species, in light of assessed antioxidant and enzyme inhibitory activities. The results of PCA were displayed in Figure 4. 88% variability of the data were captured by the first three Principal components (PCs) which each exhibited eigenvalue greater than 1. Therefore, these PCs were retained according to the method outlined by Kaiser [34]. By Referring to Sup 2, the first PC had higher correlation with more bioactivities, notably ABTS, DPPH, FRAP, CUPRAC, BuChE, amylase and glucosidase. The second PC was predominated by MCA, AChE and tyrosinase while the third PC was dominated by PBD and MCA. From the three score plots summarized in Figure 4A, a tendency to differentiate certain groups was noted. Hence, in PC1 vs. PC3 and PC2 vs PC3, extracts from *A. aleppica* roots EA and MeOH and *A. santolinoides* roots EA were grouped together. Similarly, in PC1 vs PC2 and PC1 vs. PC3, *A. A. santolinoides* roots MeOH and *A. aleppica* aerial parts MeOH were close together. Following PCA, a hierarchical classification was done to obtain a clearer picture of the different group. Based on the scores of samples on the three PCs, the hierarchical analysis revealed two principal clusters, each of which was divided into two sub-clusters (Figure 4B). The samples of the first cluster (*A. aleppica* roots water*, A. aleppica* aerial parts water*, A. santolinoides* roots water, *A. santolinoides* aerial parts water, *A. santolinoides* roots MeOH and *A. aleppica* aerial parts MeOH) were characterized by higher antioxidant activity while samples of the second cluster (*A. aleppica* roots MeOH*, A. A. santolinoides* roots EA, *A. wilhelmsii, A. aleppica* roots EA, *A. A. santolinoides* aerial parts MeOH, *A. A. santolinoides* aerial parts EA and *A. aleppica* aerial parts EA) were marked by stronger enzyme inhibitory activity.

**Figure 5:**
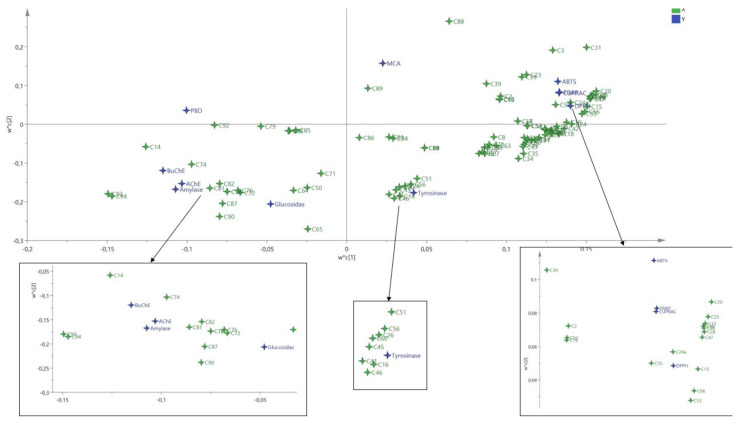
The loading plot obtained from Partial least squared regression describing relationship between chemical molecules and biological activities. (C1) protocatechuic acid-O-hexoside, (C2) caffeoylgluconic acid, (C3) protocatechuic acid, (C4) p-hydroxyphenylacetic acid-O-hexoside, (C5) protocatechuic acid-O-hexoside isomer, (C6) syringic acid 4-O-hexoside (C7) neochlorogenic (3-caffeoylquinic) acid, (C8) caffeoylgluconic acid isomer, (C9) caffeic acid-O-hexoside, (C10) gentisic acid-O-hexoside, (C11) vanillic acid 4-O-hexoside, (C12) caffeoylgluconic acid isomer, (C13) O-caffeoyl hexose isomer, (C14) 4-hydroxybenzoic acid, (C15) 4-hydroxybenzoic acid-hexoside, (C16) p-hydroxyphenylacetic acid O-hexoside, (C17) quinic acid, (C18) chlorogenic (5-caffeoylquinic) acid, (C19) p-coumaric acid, (C20) 3-feruloylquinic acid, (C21) p-hydroxyphenylacetic acid, (C22) caffeic acid, (C23) gentisic acid, (C24) 5-p-coumaroylquinic acid, (C24a) 1,3-dicaffeoylquinic acid, (C25) caffeic acid-O-hexoside isomer, (C26) 5-feruloylquinic acid, (C27) m-coumaric acid, (C28) 5-p-coumaroylquinic acid isomer, (C29) 4-feruloylquinic acid, (C30) vanillic acid, (C31) o-coumaric acid, (C32) vanillic acid-4-O-(6-O-caffeoyl)-hexoside, (C33) 3,4-dicaffeoylquinic acid, (C34) 1,5-dicaffeoylquinic acid, (C35) 3,5-dicaffeoylquinic acid, (C36) dicaffeoyl-tetrahydroxy-pentanoic acid, (C37) 4,5-dicaffeoylquinic acid, (C38) shikimic acid, (C39) salicylic acid, (C40) 3-feruloyl-4-caffeoylquinic acid, (C41) 3-p-coumaroyl-5-caffeoylquinic acid, (C42) caffeic acid-O-(salicyl)-hexoside, (C43) 3-feruloyl-5-caffeoylquinic acid, (C44) 4-p-coumaroyl-5-caffeoylquinic acid, (C45) 1-caffeoyl-3-feruloylquinic acid, (C46) 3,4,5-tricaffeoylquinic acid, (C47) 6, 8-diC-hexosidyl-luteolin, (C48) O,C-dihexosyl-luteolin, (C49) diC-hexosyl-apigenin, (C50) 6-C-hexosyl-8-C-pentosyl-luteolin, (C51) 2″-O-pentosyl-6-C-hexosyl-luteolin, (C52) homoorientin, (C53) 6-C-hexosyl-8-C-pentosyl apigenin, (C54) orientin (luteolin-8-C-glucoside), (C55) C-hexosyl-C-pentosyl methylluteolin, (C56) rutin, (C57) vitexin, (C58) isovitexin, (C59) 2″-O-pentosyl-6-C-hexosyl-methylluteolin, (C60) Luteolin-7-O-glucosidea, (C61) chrysoeriol-6-C-hexoside, (C62) nepetin-O-hexuronide, (C63) 6-methoxykaempferol-O-hexoside, (C64) nepetin-O-hexoside, (C65) kaempferol-3-O-glucoside, (C66) isorhamnetin 3-O-glucoside, (C67) apigenin-7-O-glucoside, (C68) cirsiliol-O-hexoside, (C69) chrysoeriol-O-hexuronide, (C70) jaceosidin-O-hexuronide, (C71) luteolin, (C72) quercetin, (C73) patuletin (6-methoxyquercetin), (C74) axillarin, (C75) apigenin, (C76) kaempferol, (C77) hispidulin (scutellarein-6-methyl ether), (C78) chrysoeriol, (C79) cirsiliol, (C80) quercetagetin-3,6,3′(4′)-trimethyl ether, (C81) cirsimaritin (6-hydroxyapigenin-6,7-dimethyl ether), (C82) santin/eupatilin, (C83) acacetin, (C84) tanaparthin-peroxide, (C85) achillicin/matricin, (C86) dehydroachillin/dehydroleucodin, (C87) achillin/leucodin, (C88) artabsin, (C89) dihydrosantamarin, (C90) tetradecenoic acid amide, (C91) linolenamide, (C92) linoleamide, (C93) palmitamide, (C94) oleamide.

The relationship between the metabolites and biological activities Partial was assessed and result was reported in Figure 5. As can be observed, the different biological activities were related to the synergetic action of various metabolites. In depth examination, antioxidant activities of the species could be result from the synergetic action of metabolites such as (C12) caffeoylgluconic acid isomer, (C15) 4-hydroxybenzoic acid-hexoside, (C20) 3-feruloylquinic acid, (C24a) 1,3-dicaffeoylquinic acid, (C25) caffeic acid-O-hexoside isomer, (C28) 5-p-coumaroylquinic acid isomer, (C40) 3-feruloyl-4-caffeoylquinic acid, (C47) 6, 8-diC-hexosidyl-luteolin, (C48) O,C-dihexosyl-luteolin, (C53) 6-C-hexosyl-8-C-pentosyl apigenin, (C55) C-hexosyl-C-pentosyl methylluteolin, (C66) isorhamnetin 3-O-glucoside while tyrosinase inhibitory activity of the species may be from (C16) p-hydroxyphenylacetic acid O-hexoside, (C26) 5-feruloylquinic acid, (C41) 3-p-coumaroyl-5-caffeoylquinic acid, (C45) 1-caffeoyl-3-feruloylquinic acid, (C46) 3,4,5-tricaffeoylquinic acid, (C51) 2″-O-pentosyl-6-C-hexosyl-luteolin, (C56) rutin, (C60) Luteolin-7-O-glucoside. Similarly, anti-amylase, anti-AChE and anti-BChE activities were probably arise from the action of (C14) 4-hydroxybenzoic acid, (C72) quercetin, (C74) axillarin, (C76) kaempferol, (C77) hispidulin (scutellarein-6-methyl ether), (C78) chrysoeriol, (C81) cirsimaritin (6-hydroxyapigenin-6,7-dimethyl ether), (C82) santin/eupatilin. Partial least-squares regression model resumed about 0.88% and 0.87% of the total variation in metabolites (R^2^X) and biological activities (R^2^Y) respectively, indicating the good performance of the model.

### 3.5. KEGG Analysis

**Figure 6:**
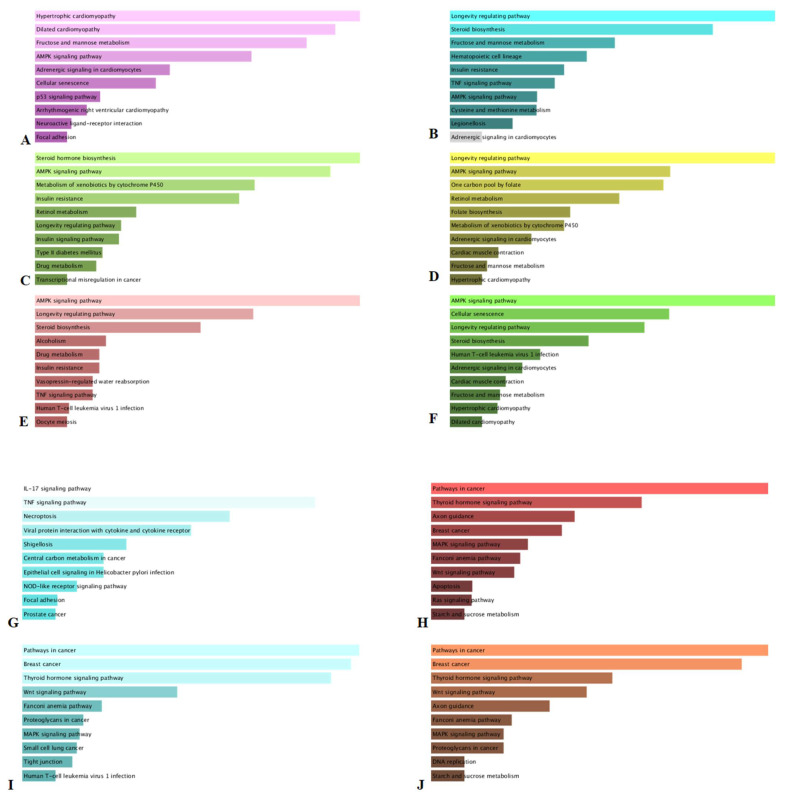
Top 10 KEGG pathway enrichment for the selected compounds. The bars in the panels represents the *p*-values computed using the Fisher exact test. The longer bars and lighter colored bars mean that the term is more significant. (**A**) artabsin; (**B**) dehydroleucodin; (**C**) dihydrosantamarin; (**D**) leucodin; (**E**) matricin; (**F**) tanaparthin peroxide; (**G**) neochlorogenic acid and chlorogenic acid; (**H**) homoorientin; (**I**) vitexin; (**J**) isovitexin.

After the phytochemical screening and in vitro evaluation of biological properties of the samples, we have been engaged in the investigation of KEGG pathway enrichment analysis of identified sesquiterpene lactones and derivatives and five of the main phenolics of *Achillea* species. In respect of the genes modulation, 73, 122, 280, 122, 113, 122, 57, 57, 254, 287 and 272 mRNA were found to be up-regulated and down-regulated by artabsin, dehydroleucodin, dihydrosantamarin, leucodin, matricin, tanaparthin peroxide, neochlorogenic acid, chlorogenic acid, homoorientin, vitexin and isovitexin respectively (Table S3). As regards the first enriched pathway, “hypertrophic cardiomyopathy”, “longevity regulating pathway”, “steroid hormone biosynthesis”, “AMPK signaling pathway”, “IL-17 signaling pathway” and “pathways in cancer” were found to be modulated by the mRNA targeted by artabsin, dehydroleucodin, dihydrosantamarin, leucodin, matricin, tanaparthin peroxide, neochlorogenic acid, chlorogenic acid, homoorientin, vitexin and isovitexin respectively (Figure 6). Structure of these compounds are reported in Figure 3. Moreover, it is worth noting that “AMPK signaling pathway” was predicted to be regulated by nine compounds except neochlorogenic acid and chlorogenic acid. AMP-activated protein kinase (AMPK) is one of the central mediators of cellular and organismal metabolism. It has key roles in promoting catabolic pathways to produce more ATP and in inhibiting anabolic pathways [58]. Once activated, AMPK leads to a concomitant activation of ATP-producing catabolic pathways, such as glycolysis and fatty acid oxidation and inhibition of energy-consuming biosynthetic pathways, such as fatty acid, protein, and glycogen synthesis. Otherwise, AMPK is a known target for treating type-2 diabetes and metabolic syndrome and for reducing the incidence of cancer [59]. Sesquiterpenes lactones have been reported to induce an anticancer actions through an impact on multiple signaling pathways as well as a changes in the redox cell balance [60]. These effects lead to the increase in apoptotic factors and the reduction of metastasis, cellular invasion and anti-apoptotic factors. Illustratively, earlier study demonstrated the potentiality of matricin to significantly exert anti-proliferative and apoptosis-inducing effects in non-small cell lung cancer cells via activation of MAPK pathway [61]. Additionally, expression of anti-apoptotic proptein Bcl-2 was significantly decreased while the level of pro-apoptosis protein Bax as well as the activity of apoptosis marker enzymes caspase-9, caspase-8 and caspase-3 were significantly increased. Similarly, in a study of the anti-alcoholic liver disease activity of leucodin isolated from *Artemisia capillaries*, it has been demonstrated that leucodin dose dependently enhances phosphorylation of AMPK in alcohol-exposed HepG2 cells [62]. Furthermore, homoorientin has been demonstrated to have anti-pancreatic cancer activity via the AMPK signaling pathways [63]. While the literature has reported multiple biological mechanism, notably the regulation of AMPK pathway, to explain the pharmacological activities of vitexin and isovitexin [64]. This finding demonstrated that homoorientin, vitexin, isovitexin and both sesquiterpene lactones compounds can modulate AMPK signaling pathway. Hence, the nine compounds present in the different parts of both studied species could serve as AMPK activators and could be a promising candidate for the prevention and treatment of cancer. However, further studies on purified compounds will be necessary to confirm the conclusions of the present bioinformatics study.

## 4. Conclusions

This study allowed obtaining a detailed phytochemical fingerprint of *A.* *aleppica* and *A. santolinoides* roots and aerial part. Chlorogenic acid was the main derivative in aerial parts of both the species. 3,5-diCQA was the most important *di*CQA derivative in *A. aleppica* while 1,3*di*CQA was the most significant in *A santolinoides*. Sesquiterpene lactone and fatty acid amides have been also detected showing large chemical diversity in the constituents of the plant. The extraction with ethyl acetate, methanol and water allowed to prepare samples with different composition that were used to assess their in vitro bioactivity on several antioxidant and enzyme inhibition assays. The methanol extract of *A. santolinoides* roots possessed significant antioxidant activities. The ethyl acetate extracts of the aerial parts and roots of both *Achillea* species showed significant inhibition against butyrylcholinesterase while the ethyl acetate extract of *A. santolinoides* aerial part actively inhibited tyrosinase. The detailed phytochemical investigation, the evaluation of in vitro bioactivity, of the two *Achillea* species indicate these plants as valuable starting point for potential future studies and possible applications extracts in cosmetic, pharmaceuticals and nutraceuticals products. KEGG mapping using some of the phenolics and sesquiterpenes of the plants allowed to predict some of the possible molecular targets for significant bioactivities. This information opens new opportunities of research and application for *A. aleppica* and *A. santolinoides* extracts and isolated compounds.

## Figures and Tables

**Table 1:** Extraction yields (%), total bioactive compounds and total antioxidant capacity (by phosphomolybdenum assays) of the tested extracts *.

Species	Parts	Solvents	Yields	TPC (mg GAE/g)	TFC (mg RE/g)	PBD (mmol TE/g)
A. aleppica	Aerial parts	EA	4.61	20.77 ± 0.83 ^e^	13.23 ± 0.52 ^c^	2.33 ± 0.09 ^a^
		MeOH	10.01	41.41 ± 0.88 ^b^	49.18 ± 0.98 ^a^	1.92 ± 0.04 ^b^
		Water	9.88	36.56 ± 0.01 ^c^	16.62 ± 0.17 ^b^	1.45 ± 0.05 ^d^
	Roots	EA	1.88	22.41 ± 0.59 ^d^	3.95 ± 0.11 ^e^	1.65 ± 0.04 ^c^
		MeOH	2.85	23.83 ± 0.24 ^d^	5.93 ± 0.04 ^d^	1.27 ± 0.03 ^e^
		Water	4.58	43.24 ± 0.19 ^a^	4.12 ± 0.05 ^e^	1.57 ± 0.04 ^cd^
A. santolinoides	Aerial parts	EA	7.40	20.69 ± 0.32 ^f^	19.58 ± 0.32 ^a^	1.95 ± 0.05 ^a^
		MeOH	16.04	32.20 ± 0.22 ^d^	8.42 ± 0.63 ^d^	1.90 ± 0.09 ^ab^
		Water	19.05	44.97 ± 0.49 ^c^	18.08 ± 0.23 ^b^	1.33 ± 0.03 ^c^
	Roots	EA	1.41	26.27 ± 0.90 ^e^	5.07 ± 0.23 ^e^	1.73 ± 0.10 ^b^
		MeOH	6.92	52.07 ± 1.58 ^a^	11.09 ± 0.18 ^c^	1.93 ± 0.12 ^ab^
		Water	4.91	47.39 ± 0.05 ^b^	3.59 ± 0.27 ^f^	1.88 ± 0.04 ^ab^

* Values are reported as mean ± SD. EA: Ethyl acetate; MeOH: Methanol; TPC: Total phenolic content; TFC: Total flavonoid content; PBD: Phosphomolybdenum; GAE: Gallic acid equivalent; RE: Rutin equivalent; TE: Trolox equivalent. Different letters in same column indicate significant differences for each *Achillea* species (*p* < 0.05).

**Table 2:** Secondary metabolites in the studied *Achillea* extracts by UHPLC-ESI-MS/MS. Compound distribution is reported in the last column and different part and extracts are numberedas follows: 1. *A. allepica*-Aerial parts-EA; 2. *A. allepica*-Aerial parts-MEOH; 3. *A. allepica*-Aerial parts-WATER, 4. *A. allepica*-Roots-EA, 5. *A. allepica*-Roots-MEOH, 6. *A. allepica*-Roots-WATER, 7. *A. santolinoides*-Aerial parts-EA, 8. *A. santolinoides*-Aerial parts-MEOH, 9. *A. santolinoides*-Aerial parts-WATER, 10. *A. santolinoides*-Roots-EA, 11. *A. santolinoides*-Roots-MEOH, 12. *A. santolinoides*-Roots-WATER. ^a,b^ compound identity is confirmed by comparison with reference standards.

No.	Identified/Tentatively Annotated Compound	Molecular Formula	Exact Mass [M − H]^−^	Fragmentation Pattern In (−) ESI-MS/MS	t_R_ (Min)	Δ ppm	Distribution
Hydroxybenzoic, hydroxycinnamic and acylquinic acids, and derivatives							
1	protocatechuic acid-*O*-hexoside	C_13_H_16_O_9_	315.0722	315.0725 (100), 153.0179 (30.5), 109.0279 (99.3)	1.71	0.385	2,3,4,5,6,7,8,9,10,11,12
2	caffeoylgluconic acid	C_15_H_18_O_10_	357.0827	357.0827 (8.1), 195.0503 (100), 179.0340 (27.2), 177.0397 (18.2), 135.0440 (25.0), 87.0073 (3.6), 59.0121 (11.1)	2.01	−0.020	2,3,8,9
3	protocatechuic acid ^a^	C_7_H_6_O_4_	153.0182	153.0180 (14.6), 123.0435 (100), 109.0278 (40.8)	2.03	−1.362	1,2,3,4,5,6,7,8,9,10,11,12
4	*p*-hydroxyphenylacetic acid-*O*-hexoside	C_14_H_18_O_8_	313.0929	313.0923 (2.7), 151.0387 (100), 123.0071 (0.9)	2.67	−0.591	1,2
5	protocatechuic acid-*O*-hexoside isomer	C_13_H_16_O_9_	315.0722	315.0723 (100), 153.0180 (60.3), 123.0437 (17.1), 109.0279 (75.9)	2.14	0.145	2,3,4,5,6,8,9,10,11,12
6	syringic acid 4-*O*-hexoside	C_15_H_20_O_10_	359.0984	359.0984 (9.1), 197.0445 (100), 182.0210 (19.2), 166.9974 (7.6), 153.0544 (14.8), 138.0307 (28.5), 123.0072 (32.0)	2.28	−0.010	1,2,3,4,5,6,9,10
7	neochlorogenic (3-caffeoylquinic) acid ^a^	C_16_H_18_O_9_	353.0867	353.0879 (42.4), 191.0551 (100), 179.0339 (60.4), 173.0444 (3.7), 161.0236 (4.2), 135.0437 (53.1), 127.0387 (2.4), 93.0331 (4.9), 85.0277 (9.9)	2.31	0.115	2,3,4,5,6,7,8,9,10,11,12
8	caffeoylgluconic acid isomer	C_15_H_18_O_10_	357.0827	357.0810 (4.8), 195.0500 (72.1), 179.0338 (100), 177.0395 (7.3), 161.0234 (1.1), 135.0437 (77.2), 129.0177 (2.2), 87.0070 (5.6), 59.0124 (2.4)	2.40	−1.730	2,3,5,8,9
9	caffeic acid*-O*-hexoside	C_15_H_18_O_9_	341.0867	341.0880 (5.0), 179.0338 (100), 135.0436 (62.0)	2.40	0.195	2,3,4,5,6,8,9
10	gentisic acid-*O*-hexoside	C_13_H_16_O_9_	315.0722	315.0724 (33.5), 153.0180 (70.9), 135.0072 (4.3), 109.0279 (100), 91.0171 (0.4)	2.58	0.205	2,3,4,5,6,7,8,9,11,12
11	vanillic acid 4-*O*-hexoside	C_14_H_18_O_9_	329.0878	329.0878 (27.1), 197.0446 (100), 182.0210 (15.5), 167.0335 (5.5), 153.0544 (28.7), 123.0073 (19.0),	2.69	-0.035	2,3,4,5,6,7,8,9,11,12
12	caffeoylgluconic acid isomer	C_15_H_18_O_10_	357.0827	357.0828 (23.9), 339.0726 (11.2), 195.0500 (100), 179.0339 (18.9), 177.0392 (16.9), 161.0235 (3.4), 135.0437 (22.5), 129.0174 (9.7), 87.0071 (10.6), 59.0124 (1.4)	2.81	0.044	2,3,5,6,8,9,12
13	*O*-caffeoyl hexose isomer	C_15_H_18_O_9_	341.0867	341.0869 (23.7), 281.0665 (94.7), 251.0557(54.2), 221.0448 (44.0), 179.0339 (100), 161.0231 (56.9), 135.0437 (72.4)	2.82	−0.955	2,5,8,9
14	4-hydroxybenzoic acid ^a^	C_7_H_6_O_3_	137.0244	137.0229 (12.6), 108.0208 (0.1), 93.0329 (100)	2.86	−1.527	1,2,3,4,5,6,7,8,9,10,11,12
15	4-hydroxybenzoic acid-hexoside	C_13_H_16_O_8_	299.0772	299.0773 (1.5), 137.0230 (100), 93.0330 (54.3)	3.00	0.029	1,2,3,4,5,6,7,8,9,10,11,12
16	*p*-hydroxyphenylacetic acid *O*-hexoside	C_14_H_18_O_8_	313.0929	313.0932 (13.4), 151.0386 (100), 123.0070 (0.9)	3.00	0.309	2,5,11
17	quinic acid	C_7_H_12_O_6_	191.0561	191.0550 (100), 173.0444 (2.0), 155.0332 (0.3), 127.0386 (4.0), 111.0436 (1.6), 93.0330 (6.2), 85.0279 (19.1)	3.16	−1.101	2,3,4,5,6,8,9,11,12
18	chlorogenic (5-caffeoylquinic) acid ^a^	C_16_H_18_O_9_	353.0867	353.0857 (1.9), 191.0550 (100), 161.0230 (1.5), 93.0331 (1.5), 85.0278 (8.8)	3.19	0.835	1,2,3,4,5,6,7,8,10,11,12
19	*p*-coumaric acid ^a^	C_9_H_8_O_3_	163.0389	163.0385 (12.4), 135.0438 (4.2), 119.0487 (100)	3.35	−1.527	2,3,9
20	3-feruloylquinic acid ^b^	C_17_H_20_O_9_	367.1035	367.1035 (22.0), 193.0496 (100), 191.0550 (2.6), 173.0443 (3.9), 134.0358 (64.7), 93.0329 (1.4), 85.0281 (0.9)	3.44	−0.005	2,3,5,6,8,10,11,12
21	p-hydroxyphenylacetic acid ^a^	C_8_H_8_O_3_	151.0401	151.0387 (100), 107.0486 (1.4), 136.0154 (1.5), 123.0072 (4.2)	3.48	1.397	2,3,4,5,6,7,8,9,10,12
22	caffeic acid ^a^	C_9_H_8_O_4_	179.0338	179.0339 (3.6), 135.0437 (100), 151.0754 (2.4), 107.0489 (1.6)	3.56	−1.092	1,2,3,4,5,6,7,8,10,11,12
23	gentisic acid ^a^	C_7_H_6_O_4_	153.0182	153.0180 (73.8), 135.0073 (31.4), 109.0279 (100), 91.0173 (6.3)	3.87	−1.372	1,23,5,6,8,9,10,11,12
24	5-*p*-coumaroylquinic acid	C_16_H_18_O_8_	337.0929	337.0933 (9.3), 191.0550 (100), 173.0444 (6.8), 163.0388 (6.1), 161.0229 (0.2), 127.0385 (1.2), 119.0487 (5.3), 93.0329 (17.9), 85.0278 (5.1)	3.96	0.369	1,2,3,4,5,6,8,9,11,12
24a	1,3-dicaffeoylquinic acid	C_25_H_24_O_12_	515.1195	115.1199 (78.8), 353.0873 (36.6), 335.0779 (10.9), 191.0550 (100), 179.0338 (73.2), 173.0452 (5.6), 161.0232 (9.9), 135.0435 (58.7), 111.0434 (1.7)	4.13	0.401	1,2,3,4,5,6,8,9,11,12
25	caffeic acid-*O*-hexoside isomer	C_15_H_18_O_9_	341.0867	341.0830 (5.5), 179.0335 (6.2), 161.0230 (39.2), 135.0436 (63.6)	4.34	−4.765	2,3,5,6,8,9,10,12
26	5-feruloylquinic acid	C_17_H_20_O_9_	367.1035	367.1035 (18.5), 193.0498 (8.3), 191.0552 (100), 173.0444 (24.1), 134.0359 (12.2), 127.0382 (1.0), 111.0436 (5.0), 93.0329 (30.0), 85.0278 (6.0)	4.42	−0.015	2,3,4,5,7,9,10,11,12
27	*m*-coumaric acid ^a^	C_9_H_8_O_3_	163.0389	163.0387 (9.0), 135.0434 (1.8), 119.0486 (100)	4.57	−1.367	2,3,4,5,6,8,9,10,11
28	5-*p*-coumaroylquinic acid isomer	C_16_H_18_O_8_	337.0929	337.0934 (7.0), 191.0550 (100), 173.0444 (1.9), 163.0387 (2.2), 127.0385 (2.2), 119.0487 (1.6), 111.0434 (1.3), 93.0332 (5.0), 85.0278 (8.1)	4.62	0.489	2,3,5,6,8,9,12
29	4-feruloylquinic acid	C_17_H_20_O_9_	367.1035	367.1034 (89.4), 193.0497 (9.8), 173.0443 (63.1), 155.0338 (4.1), 134.0358 (21.8), 111.0436 (14.7), 93.0329 (100), 85.0276 (0.5)	4.66	−0.055	2,3,4,5,6,10,11,12
30	vanillic acid ^a^	C_8_H_8_O_4_	167.0350	167.0338 (100), 152.0101 (27.8), 123.0071 (4.8), 95.0124 (3.4)	4.79	−1.232	2,3,4,5,6,7,8,9,11,12
31	*o*-coumaric acid ^a^	C_9_H_8_O_3_	163.0389	163.0387 (19.5), 135.0441 (4.1), 119.0487 (100)	4.84	−1.367	2,3,6
32	vanillic acid-4-*O*-(6-*O*-caffeoyl)-hexoside ^b^	C_23_H_24_O_12_	491.1195	491.1209 (100.0), 323.0774 (23.3), 221.0458 (4.6), 179.0343 (10.6), 167.0338 (16.2), 161.0231 (38.9), 152.0101 (18.3), 135.0437 (14.8), 123.0436 (1.2)	5.52	0.928	2,3,4,5,6,8,9,11
33	3,4-dicaffeoylquinic acid ^a^	C_25_H_24_O_12_	515.1195	515.1179 (94.1), 353.0875 (62.2), 335.0771 (6.7), 299.0573 (13.6), 203.0339 (41.1), 191.0548 (32.6), 179.0339(76.0), 173.0444 (100), 161.0233 (13.6), 135.0437 (77.0), 111.0436 (4.2), 93.0330 (38.4), 85.0278 (3.9)	5.60	−1.579	2,3,4,5,6,8,9,10,11,12
34	1,5-dicaffeoylquinic acid ^a^	C_25_H_24_O_12_	515.1195	515.1189 (15.1), 353.0878 (33.0), 335.0774 (2.2), 191.0550 (100), 179.0338(6.2), 173.0446 (3.1), 161.0231 (5.0), 135.0436 (6.6), 127.0382 (1.8), 111.0433 (1.1), 93.0331 (4.5), 85.0278 (7.6)	5.70	−0.599	1,2,3,4,5,6,7,8,9,10,11,12
35	3,5-dicaffeoylquinic acid ^a^	C_25_H_24_O_12_	515.1195	515.1204 (22.6), 353.0878 (100), 191.0551 (96.5), 179.0338 (53.1), 173.0441 (5.3), 161.0229 (7.9), 135.0437 (52.7), 111.0433 (1.1), 93.0328 (4.7), 85.0279 (9.1)	5.86	0.921	1,2,3,4,5,6,7,8,9,10,11,12
36	dicaffeoyl-tetrahydroxy-pentanoic acid	C_23_H_22_O_12_	489.1038	489.1030 (43.3), 327.0720 (40.6), 165.0392 (100), 179.0341 (17.2), 161.0231 (3.2),	6.12	−0.849	2,3,5,8,9
37	4,5-dicaffeoylquinic acid ^a^	C_25_H_24_O_12_	515.1195	515.1204 (84.6), 353.0877 (76.6), 191.0549 (50.2), 179.0338 (72.4), 173.0443 (100), 161.0232 (8.0), 111.0435 (2.0), 93.0330 (27.1), 85.0278 (4.2)	6.23	0.901	1,2,3,4,5,6,7,8,9,10,11,12
38	shikimic acid	C_7_H_10_O_5_	173.0455	173.0444 (100), 155.0335 (2.0), 137.0232 (1.4), 127.0390 (0.5), 111.0437 (10.0), 93.0330 (68.4)	6.23	−6.453	2,3,5,8,9,11
39	salicylic acid ^a^	C_7_H_6_O_3_	137.0244	137.0228 (15.2), 109.0279 (0.7), 93.0330 (100)	6.29	−1.467	1,2,3,4,5,6,7,8,9,10,11,12
40	3-feruloyl-4-caffeoylquinic acid	C_26_H_26_O_12_	529.1351	529.1339 (100), 335.075 (9.3), 193.0498 (60.1), 191.0558 (7.6), 179.0336 (29.5), 173.0441 (29.3), 161.0231 (8.7), 135.0434 (32.1), 134.0357 (49.5), 93.0330 (6.8)	6.49	−1.299	2,3,5,6,9,11,12
41	3-*p*-coumaroyl-5-caffeoylquinic acid	C_25_H_24_O_11_	499.1246	499.1388 (44.0), 353.0885 (5.4), 337.0932 (83.6), 191.0553 (31.7), 173.0443 (7.0), 163.0388 (100.0), 135.0429 (1.7), 93.0326 (7.6), 85.0278 (1.8)	6.53	14.205	2,3,4,5,8,9,11
42	caffeic acid-*O*-(salicyl)-hexoside	C_22_H_21_O_11_	461.1089	461.1093 (49.1), 371.0756 (0.5), 341.0656 (1.9), 323.0774 (24.5), 299.0767 (1.6), 179.0340 (5.1), 161.0231 (23.1), 137.0229 (100), 93.0330 (61.3)	6.56	0.405	1,2,3,4,5,6,7,8,9,11,12
43	3-feruloyl-5-caffeoylquinic acid	C_26_H_26_O_12_	529.1351	529.1355 (17.3), 367.1033(3.0), 353.2703 (1.6), 335.0754 (1.5), 193.0496 (100), 191.0554 (8.0), 173.0450 (9.0), 161.0230 (10.50), 134.0359 (74.4), 93.0331 (2.6)	6.82	0.351	2,3,4,5,6,8,9,10,12
44	4-*p*-coumaroyl-5-caffeoylquinic acid	C_25_H_24_O_11_	499.1246	499.1218 (-0.871), 337.0932 (61.7), 179.0339 (9.0), 173.0442 (100), 163.0390 (21.2), 135.0437 (4.7), 119.0487 (8.7), 111.0437 (2.8)	6.90	−2.755	2,3,4,5,6,8,9,11
45	1-caffeoyl-3-feruloylquinic acid	C_26_H_26_O_12_	529.1351	529.1355 (17.3), 367.1033(3.0), 353.2703 (1.6), 335.0754 (1.5), 193.0496 (100), 191.0554 (8.0), 173.0450 (9.0), 161.0230 (10.50), 134.0359 (74.4), 93.0331 (2.6)	7.23	0.651	2,4,5,10
46	3,4,5-tricaffeoylquinic acid	C_34_H_30_O_15_	677.1512	677.1509 (100). 515.1186 (32.4), 353.0879 (40.3), 335.0752 (13.7), 191.0552 (36.9), 179.0338 (70.6), 173.0444 (69.4), 161.0231 (19.8), 135.0437 (74.8), 111.0442 (3.6), 93.0330 (17.5)	7.80	−0.253	2,5,8,9,11
Flavonoids							
47	6, 8-di*C*-hexosidyl-luteolin	C_27_H_30_O_16_	609.1461	609.1467 (100), 519.1136 (4.1), 489.1045 (14.6), 471.0941 (0.9), 429.0831 (6.1), 399.0722 (24.4), 369.0617 (26.2), 339.0507 (3.5), 311.0547 (5.2), 175.0387 (1.2), 133.0283 (6.7)	3.64	0.622	2,3,5,6,8,9,10,11,12
48	*O*,*C*-dihexosyl-luteolin	C_27_H_30_O_16_	609.1461	609.1469 (100), 447.0930 (24.2), 387.0808 (1.1), 369.0595 (1.6), 357.0616 (16.3), 327.0509 (54.5), 299.0557 (10.2), 298.0480 (6.7), 297.0403 (5.5), 175.0386 (1.6), 133.0283 (6.8)	3.87	0.742	2,3,5,6,8,9,12
49	di*C*-hexosyl-apigenin	C_27_H_30_O_15_	593.1512	593.1518 (100), 503.1205 (3.9), 473.1089 (14.9), 455.0996 (1.5), 413.0878 (1.9), 383.0773 (14.7), 353.0667 (31.6), 325.0723 (2.4), 309.0763 (1.8), 297.0769 (12.4), 175.0389 (1.5), 117.0331 (4.4)	4.03	0.597	2,3,4,5,6,8,9,10,11,12
50	6*-C*-hexosyl-8-*C*-pentosyl-luteolin	C_26_H_28_O_15_	579.1355	579.1362 (100), 519.1219 (1.4), 489.1044 (10.7), 471.0909 (2.3), 459.0936 (9.1), 441.0836 (4.1), 429.0844 (7.1), 411.0721 (2.6), 399.0720 (26.7), 381.0613 (2.3), 369.0617 (24.6), 339.0504 (4.7), 311.0559 (4.3), 298.0483 (4.5), 175.0390 (0.9), 133.0280 (5.5)	4.12	0.627	1,2,3,4,5,7,8,9,10,11,12
51	2”-*O*-pentosyl-6-*C*-hexosyl-luteolin	C_26_H_28_O_15_	579.1355	579.1362 (100), 459.0923 (9.6), 429.0820 (3.7), 399.0729 (1.4), 369.0618 (3.7), 357.0618 (25.8), 327.0514 (48.0), 309.0394 (7.2), 299.0558 (6.5), 298.0485 (17.0), 297.0400 (10.8), 175.0392 (1.4), 133.0278 (9.7)	4.47	0.687	2,3,4,5,7,8,9,10,11
52	Homoorientin ^a^	C_21_H_20_O_11_	447.0933	447.0935 (100), 429.0830 (3.6), 411.0712 (0.9), 399.0707 (1.1), 387.0720 (0.5), 381.0609 (0.5), 369.0618 (2.8), 357.0617 (47.4), 327.0511 (68.9), 299.0559 (11.8), 298.0477 (9.1), 297.0403 (12.0), 285.0402 (8.6), 269.0449 (1.8), 199.0391 (1.5), 133.0280 (15.0), 107.0119 (0.7)	4.53	0.225	1,2,3,4,5,6,8,9,10,11
53	6*-C*-hexosyl-8-*C*-pentosyl apigenin	C_26_H_28_O_14_	563.1406	563.1412 (100), 503.1214 (3.6), 473.1079 (6.7), 443.0990 (7.5), 425.0868 (2.9), 413.0889 (2.9), 383.0774 (20.1), 365.0665 (2.0), 353.0668 (25.9), 325.0715 (2.1), 324.0595 (0.4), 323.0562 (1.4), 297.0765 (9.3), 283.0611 (2.2), 175.0393 (1.3), 135.0434 (2.0), 117.0330 (3.1)	4.53	0.541	1,2,3,4,5,6,7,8,9,10,11,12
54	orientin (luteolin-8-*C*-glucoside)	C_21_H_20_O_11_	447.0933	447.0935 (89.2), 369.0621 (3.2), 357.0616 (31.4), 327.0511 (100), 299.0560 (10.7), 298.0476 (7.0), 297.0404 (13.5), 285.0397 (6.4), 269.0457 (1.6), 133.03 (19.4), 119.0485 (1.4), 151.022 (0.7), 107.0121 (0.6)	4.68		1,2,4,5,6,8,9,10,11
55	*C*-hexosyl-*C*-pentosyl methylluteolin	C_27_H_30_O_15_	593.1512	593.1520 (100), 503.1203 (9.8), 473.1092 (11.1), 443.0963 (2.7), 425.0835 (1.0), 413.0881 (14.7), 395.0765 (0.6), 383.0775 (24.8), 341.0677 (1.1), 323.0550 (1.1), 313.0683 (1.9), 312.0639 (18.5), 299.0557 (0.5), 298.0476 (2.4), 283.0614 (1.8)	4.75	0.787	1,2,3,4,5,6,7,8,9,10,11,12
56	Rutin ^a^	C_27_H_30_O_16_	609.1461	609.1464 (100), 301.0347 (39.7), 300.0274 (70.1), 271.0247 (39.2), 255.0297 (18.2), 243.0294 (9.3), 227.0342 (2.7), 211.0394 (0.9), 178.9977 (3.2), 163.0027 (1.6), 151.0024 (7.1), 121.0278 (1.1), 107.0121 (2.2)	5.06	0.512	2,3,4,5,7,8,9,10,11,12
57	Vitexin ^a^	C_21_H_20_O_10_	431.0984	431.0986 (95.1), 341.0666 (0.5), 311.0562 (100), 293.0452 (2.0), 283.0610 (30.3), 117.0330 (15.0)	5.15	0.200	1,2,3,4,5,6,7,8,9,10,11,12
58	Isovitexin ^a^	C_21_H_20_O_10_	431.0984	431.0986 (100), 413.0880 (1.7), 341.0666 (32.8), 311.0562 (69.1), 283.0610 (22.1), 269.0447 (4.1), 239.0706 (1.3), 117.0330 (8.7)	5.30	0.200	1,2,4,5,6,7,9,10,11
59	2”-*O*-pentosyl-6-*C*-hexosyl-methylluteolin	C_27_H_30_O_15_	593.1512	593.1500 (100), 473.1140 (1.7), (443.0968 (6.1), 383.0750 (7.7), 371.0761 (16.5), 341.0664 (36.4), 323.0566 (20.7), 308.0315 (6.7), 299.0526 (2.7), 298.0486 (15.9)	5.36	0.002	1,2,3,5,6,7,8,9,10,11,12
60	Luteolin-7-*O*-glucoside ^a^	C_21_H_20_O_11_	447.0933	447.0935 (100), 285.0404 (82.4), 133.0283 (11.8)	5.39	0.437	2,4,5,8
61	chrysoeriol-6-*C*-hexoside	C_22_H_22_O_11_	461.1078	461.1095 (100), 371.0774 (24.0), 341.0667 (73.2), 298.0481 (44.3), 296.0324 (0.9), 297.0403 (14.6)	5.43	0.535	2
62	nepetin-*O*-hexuronide	C_22_H_20_O_13_	491.0832	491.0829 (72.9), 315.0511 (100), 300.0275 (54.3), 272.0326 (8.6), 243.0297 (0.9), 227.0347 (0.5), 133.0284 (2.1)	5.47	0.335	2,3,8
63	6-methoxykaempferol-*O*-hexoside	C_22_H_22_O_12_	477.1042	477.1041 (100), 315.0512 (56.5), 300.0272 (16.4), 299.0197 (18.2), 271.0247 (52.2), 243.0292 (0.7), 227.0344 (0.4), 151.0020 (1.6), 107.0122 (0.3)	5.48	0.251	1,2,3,4,5,6,7,8,9,10,11,12
64	nepetin-*O*-hexoside	C_22_H_22_O_12_	477.1038	477.1033 (100), 315.0486 (29.7), 300.0269 (15.9), 299.0197 (20.5), 271.0244 (3.6), 255.0307 (1.8), 243.0303 (2.6), 227.0344 (3.5), 165.8804 (0.5), 136.9889 (1.7), 133.0279 (10.0)	5.67	−0.549	1,2,4,5,7,8,9,11,12
65	kaempferol-3-*O*-glucoside ^a^	C_21_H_20_O_11_	447.0933	447.0935 (100), 285.0397 (22.0), 284.0324 (55.3), 255.0294 (41.8), 227.0341 (35.0), 151.0023 (1.6)	5.86	0.195	2,3,4,5,8,9
66	isorhamnetin 3-*O*-glucoside ^a^	C_22_H_22_O_12_	477.1042	477.1041 (100), 315.0493 (10.1), 314.0433 (49.0), 300.0279 (3.1), 299.0212 (4.6), 271.0245 (18.5), 255.0300 (0.8), 243.0291 (19.6), 227.0347 (2.8), 215.0350 (3.8), 151.0022 (2.42)	6.02	0.251	1,2,3,4,5,6,8,9
67	apigenin-7-*O*-glucoside ^a^	C_21_H_20_O_10_	431.0984	431.0986 (100), 269.0450 (27.7), 151.0019 (1.0), 107.0121 (1.4)	6.06	0.200	2,3,5,8,9,11
68	cirsiliol-*O*-hexoside	C_23_H_24_O_12_	491.1184	491.1198 (0.3), 476.0963 (26.6), 461.0726 (9.7), 329.0664 (5.0), 314.0425 (5.5), 313.0355 (13.1), 299.0197 (4.3), 285.0402 (11.8), 271.0245 (9.6), 243.0292 (9.0),	6.31	0.311	2
69	chrysoeriol-*O*-hexuronide	C_23_H_22_O_12_	475.0882	475.0884 (83.8), 299.0560 (100), 284.0325 (65.6), 256.0373 (6.5), 227.0347 (1.1), 175.0237 (15.3), 151.0024 (3.3), 113.0228 (37.6), 85.0278 (22.7),	6.34	0.181	2,3,4,5,6,8,9
70	jaceosidin-*O*-hexuronide	C_23_H_22_O_13_	505.0988	505.0993 (88.3), 329.0667 (100), 314.0432 (18.7), 299.0197 (36.3), 271.0247 (36.7), 243.0290 (0.6), 227.0342 (0.6), 175.0237 (13.2), 161.0229 (0.6), 113.0227 (34.1), 85.0278 (22.6)	6.34	0.566	2,3,5
71	Luteolin ^a^	C_15_H_10_O_6_	285.0405	285.0403 (100), 241.0975 (21.4), 226.075 (8.4)	7.59	−0.181	1,2,3,4,5,6,7,8,9,10,11
72	Quercetin ^a^	C_15_H_10_O_7_	301.0354	301.0353 (100), 273.0405 (1.5), 178.9975 (22.7), 151.0023 (51.2), 121.0281 (12.7), 107.0123 (13.4)	7.62	−0.036	1,2,3,4,5,7,8,9
73	patuletin (6-methoxyquercetin) ^b^	C_16_H_12_O_8_	331.0464	331.0458 (100), 316.0024 (65.9), 287.0190 (14.1), 271.0246 (3.5), 259.0238 (3.1), 243.0285 (2.7), 181.0132 (7.1),165.9885 (19.2), 139.0023 (11.2), 109.9994 (9.6)	7.72	−0.161	1,2,7,8,9
74	axillarin	C_17_H_14_O_8_	345.0616	345.0615 (99.2), 330.0381 (100), 315.0147 (48.0), 287.0196 (12.3), 243.0227 (2.6), 231.0295 (5.8), 215.0342 (4.1), 165.9897 (4.9), 149.0230 (1.2), 139.0385 (4.2), 136.9861 (1.3), 121.0280 (1.6)	8.24	−0.101	1,2,4,7,8,9
75	Apigenin ^a^	C_15_H_10_O_5_	269.0457	269.0453 (100), 225.0553 (1.6), 201.0546 (0.5), 151.0023 (5.4), 149.0239 (4.4), 117.0331 (18.4), 107.0124 (4.8)	8.62	0.870	2,7,8,10,11
76	Kaempferol ^a^	C_15_H_10_O_6_	285.0405	285.0402 (100), 178.9938 (0.9), 151.0026 (1.0), 107.0121 (1.4)	8.83	−0.161	1,2,3,4,5,8
77	hispidulin (scutellarein-6-methyl ether) ^a^	C_16_H_12_O_6_	299.0563	299.0559 (62.4), 284.0324 (100), 255.0303 (1.3), 227.0471 (3.4), 212.0471 (3.2), 211.0389 (2.6), 164.9812 (2.0), 163.0005 (0.3), 149.9963 (1.1), 136.9865 (14.6), 117.0324 (1.5)	8.92	−.201	1,2,3,4,5,7,8,9,10,11
78	Chrysoeriol ^a^	C_16_H_12_O_6_	299.0562	299.0560 (93.1), 284.0324 (100), 256.0372 (6.4), 227.0344 (3.3), 211.0392 (1.8), 151.0024 (5.2), 133.0280 (1.6), 107.0122 (4.6)	8.97	−0.141	1,2,3,4,5,7,8,9,10,11
79	cirsiliol	C_17_H_14_O_7_	329.0677	329.0667(100), 314.0432 (32.6), 299.0160 (21.2), 271.0248 (7.2), 255.0294 (1.0), 243.0294 (2.7), 230.1474 (11.8), 227.0344 (2.3), 163.0024 (2.0), 136.9874 (0.4), 135.0074 (1.4), 133.0282 (8.0)	9.16	0.034	1,2,3,4,7,8,9,10,11
80	quercetagetin-3,6,3’(4’)-trimethyl ether	C_18_H_16_O_8_	359.0772	359.0773 (100), 344.0536 (90.3), 329.0304 (49.3), 314.0068 (7.9), 301.0343 (3.5), 286.0118 (34.9), 258.0168 (10.9), 230.0214 (8.3), 202.0263 (10.1), 164.9807 (1.6), 148.0146 (1.6), 136.9854 (0.4),	9.74	0.059	2,7,8,9,10
81	cirsimaritin (6-hydroxyapigenin-6,7-dimethyl ether)	C_17_H_14_O_6_	313.0719	313.0822 (100), 298.0481 (56.4), 283.0246 (57.8), 269.0455 (2.8), 255.0299 (17.8), 227.0333 (5.8), 211.0333 (2.6), 163.0024 (19.5), 117.0326 (10.6)	10.38	−0.411	1,2,3,4,5,6,7,8,9,10,11,12
82	santin/eupatilin	C_18_H_16_O_7_	343.0812	343.0822 (76.5), 328.0588 (100), 313.0355 (23.9), 298.0119 (19.4), 285.0402 (7.2), 270.0168 (24.2), 257.0085 (1.8), 254.0224 (0.7), 242.0218 (3.1), 226.0267 (1.6), 214.0266 (3.3), 198.0314 (2.3), 165.9895 (1.1), 164.9812 (0.3), 163.0020 (0.2), 136.9866 (1.9), 132.0201 (1.4)	10.68	−0.086	1,2,3,4,5,6,7,8,9,10,11
83	acacetin	C_16_H_12_O_5_	283.0612	283.0610 (100), 268.0375 (72.4), 240.0425 (5.4), 239.0342 (4.8), 151.0026 (5.2), 107.0122 (3.1)	11.44	1.036	2,7,8,9
	**Tentatively Annotated Compound**	**Molecular Formula**	**Exact Mass** **[M + H]^+^**	**Fragmentation Pattern in (+) ESI-MS/MS**	**t_R_ (min)**	**Δ ppm**	**Distribution**
				Sesquiterpene lactones and derivatives			
84	tanaparthin-peroxide	C_15_H_18_O_5_	279.1226	279.1213 (2.82), 261.1115 (39.19), 237.1117 (100), 243.1015 (12.03), 233.1169 (38.41), 221.0806 (77.86), 215.1064 (29.10), 203.0699 (79.07), 193.0857 (48.82), 187.1112 (16.07), 175.0752 (85.47), 165.0909 (47.60), 147.0802 (45.29), 123.0441 (37.47), 105.0701 (37.62), 91.0547 (36.27), 79.0548 (21.43), 67.0550 (13.10)	6.41	−0.395	3,4,7,8,9
85	achillicin/matricin	C_17_H_22_O_5_	307.1537	307.1530 (56.90), 265.1427 (14.78), 247.1324 (100), 229.1220 (34.74), 219.1376 (28.47), 201.1272 (41.37), 173.0956 (29.40), 147.0802 (52.01), 131.0852 (31.79), 105.0700 (25.38), 91.0545 (21.60), 79.0549 (16.19)	8.04	−0.913	1,2,3,4,5,6,8
86	dehydroachillin/ dehydroleucodin	C_15_H_16_O_3_	245.1170	245.1166 (100), 227.1064 (6.46), 209.0956 (6.71), 199.1115 (18.76), 181.1010 (4.52), 156.0932 (3.66), 143.0852 (3.98), 123.0804 (5.57), 105.0701 (4.23), 91.0548 (3.25), 79.0548 (1.97), 69.0341 (10.05)	9.37	−0.860	1,2,3,5,7,8,11
87	achillin/leucodin	C_15_H_18_O_2_	247.1326	247.1323 (100), 229.1213 (1.49), 219.1374 (4.62), 201.1272 (5.13), 191.1426 (4.81), 173.0959 (33.47), 158.0725 (5.02), 145.1009 (9.93), 135.0803 (3.08), 117.0699 (1.99), 107.0858 (3.94), 97.0651 (2.56), 79.0547 (2.01), 69.0341 (6.12), 55.0550 (0.66)	9.55	−1.412	1,2,3,4,5,6,7,8,9,10,11,12
88	artabsin	C_15_H_20_O_3_	249.1482	249.1479 (73.40), 231.1375 (57.58), 221.1530 (6.61), 213.1268 (8.55), 203.1428 (100), 185.1322 (37.65), 175.1116 (75.95), 161.0958 (11.74), 157.1010 (78.89), 147.1166 (52.45), 133.1012 (32.49), 119.0857 (54.68), 10.0702 (59.52), 93.0703 (28.58), 81.0704 (10.70), 67.0550 (5.91), 55.0551 (8.37)	11.30	−1.409	1,2,3,4,5,6,7,9,10,11,12
89	dihydrosantamarin	C_15_H_22_O_3_	251.1638	251.1635 (31.06), 233.1530 (42.34), 215.1428 (5.23), 205.1584 (100), 187.1478 (52.95), 177.1272 (47.19), 159.1165 (68.27), 147.1166 (20.87), 133.1010 (16.22), 119.0856 (16.77), 105.0701 (27.07), 97.0652 (17.16), 81.0704 (17.26), 67.0549 (5.80)	12.64	−1.597	1,2,3,7,8
Fatty acids amides							
90	tetradecenoic acid amide	C_14_H_25_NO	224.2006	224.2004 (100), 196.2052 (0.09), 182.1537 (0.24), 168.1380 (6.51), 151.1115 (6.61), 123.1168 (2.56), 109.1014 (3.28), 95.0495 (6.80), 81.0340 (9.65), 69.0705 (11.84), 57.0707 (14.36)	15.09	−1.387	1,2,3,4,5,6,8,10,11,12
91	linolenamide	C_18_H_31_NO	278.2473	278.2472 (100), 261.2202 (0.89), 243.2098 (1.08), 219.1740 (0.69), 167.1302 (18.76), 152.1069 (6.86), 135.1169 (1.06), 109.1009 (4.05), 95.0859 (6.54), 81.0703 (9.21), 67.0549 (15.81)	19.82	−1.801	1,3,4,5,6,12
92	linoleamide	C_18_H_33_NO	280.2631	280.2628 (100), 263.2361 (82.19), 245.2258 (64.41), 221.2253 (3.56), 189.1632 (4.79), 179.1793 (9.84), 165.1634 (15.59), 147.1167 (8.77), 133.1011 (15.10), 123.1167 (23.89), 109.1013 (44.22), 95.0859 (71.60), 81.0704 (66.64), 69.0705 (50.93), 57.0706 (23.67)	20.43	−1.432	1,2,3,4,5,6,7,8,9,10,11,12
93	palmitamide	C_16_H_33_NO	256.2631	256.2627 (100), 214.2169 (0.26), 130.1227 (0.29), 116.1070 (1.43), 102.0916 (4.15), 88.0710 (0.33), 74.0607 (2.39)	21.33	−1.683	1,2,3,4,5,6,7,8,9,10,11,12
94	oleamide	C_18_H_35_NO	282.2786	282.2784 (100), 265.2520 (29.11), 247.2415 (25.90), 226.2156 (1.06), 212.2007 (3.29), 191.1790 (5.05), 177.1636 (4.54), 163.1478 (7.65), 149.1321 (12.53), 135.1167 (15.92), 121.1013 (14.04), 97.1015 (34.81), 83.0860 (35.84), 69.0706 (48.28)	21.75	−2.059	1,2,3,4,5,6,7,8,9,10,11,12

**Table 3:** Antioxidant properties of the tested extracts *.

Species	Parts	Solvents	DPPH (mg TE/g)	ABTS (mg TE/g)	CUPRAC (mg TE/g)	FRAP (mg TE/g)	MCA (mg EDTAE/g)
A. aleppica	Aerial parts	EA	13.83 ± 0.07 ^e^	22.04 ± 1.31 ^e^	50.49 ± 3.22 ^e^	27.29 ± 0.21 ^e^	23.55 ± 1.37 ^ab^
		MeOH	55.15 ± 0.05 ^a^	88.93 ± 0.79 ^b^	151.21 ± 5.64 ^a^	101.38 ± 1.79 ^a^	21.51 ± 0.09 ^bc^
		Water	49.71 ± 1.17 ^b^	90.83 ± 0.12 ^b^	138.34 ± 1.94 ^b^	95.19 ± 0.62 ^b^	25.37 ± 0.33 ^a^
	Roots	EA	12.44 ± 0.14 ^e^	30.38 ± 1.31 ^d^	66.55 ± 3.46 ^d^	34.75 ± 1.12 ^d^	10.28 ± 1.37 ^d^
		MeOH	35.66 ± 0.29 ^d^	56.23 ± 0.79 ^c^	88.69 ± 0.57 ^c^	54.62 ± 1.10 ^c^	12.03 ± 0.76 ^d^
		Water	43.44 ± 0.35 ^c^	101.88 ± 0.98 ^a^	143.53 ± 0.75 ^ab^	93.79 ± 0.99 ^b^	20.25 ± 0.52 ^c^
A. santolinoides	Aerial parts	EA	6.57 ± 0.15 ^f^	15.31 ± 0.96 ^f^	51.59 ± 0.11 ^f^	25.96 ± 0.39 ^f^	27.37 ± 0.46 ^a^
		MeOH	30.49 ± 0.30 ^d^	42.06 ± 0.40 ^e^	104.45 ± 3.32 ^d^	50.42 ± 1.61 ^d^	26.06 ± 1.20 ^a^
		Water	51.90 ± 0.67 ^b^	95.34 ± 1.15 ^c^	164.05 ± 1.57 ^b^	105.24 ± 1.07 ^c^	21.33 ± 0.16 ^b^
	Roots	EA	15.93 ± 0.07 ^e^	50.47 ± 1.33 ^d^	85.86 ± 2.57 ^e^	43.33 ± 3.63 ^e^	12.25 ± 1.70 ^d^
		MeOH	54.11 ± 0.03 ^a^	112.53 ± 0.18 ^a^	183.55 ± 1.68 ^a^	129.92 ± 3.18 ^a^	10.72 ± 0.42 ^d^
		Water	47.59 ± 0.07 ^c^	109.04 ± 0.20 ^b^	151.23 ± 0.28 ^c^	118.50 ± 0.41 ^b^	17.59 ± 0.08 ^c^

* Values are reported as mean ± SD. EA: Ethyl acetate; MeOH: Methanol; TE: Trolox equivalent; EDTAE: EDTA equivalents. Different letters in same column indicate significant differences for each *Achillea* species (*p* < 0.05).

**Table 4:** Enzyme inhibitory effects of the tested extracts *.

Species	Parts	Solvents	AChE (mg GALAE/g)	BuChE (mg GALAE/g)	Tyrosinase (mg KAE/g)	Amylase (mmol ACAE/g)	Glucosidase (mmol ACAE/g)
*A. aleppica*	Aerial parts	EA	2.63±0.03 ^a^	6.07±0.14 ^a^	57.63±1.17 ^cd^	0.29±0.04 ^b^	0.64±0.01 ^d^
		MeOH	2.01±0.21 ^bc^	2.12±0.25 ^c^	71.22±0.57 ^a^	0.22±0.01 ^c^	0.78±0.01 ^b^
		Water	0.48±0.04 ^d^	3.83±0.01 ^b^	54.86±2.16 ^d^	0.07±0.01 ^d^	na
	Roots	EA	2.21±0.10 ^b^	6.73±0.25 ^a^	63.26±0.93 ^b^	0.37±0.02 ^a^	0.70±0.04 ^c^
		MeOH	1.83±0.04 ^c^	3.92±0.54^b^	70.36±0.30 ^a^	0.24±0.01 ^c^	0.85±0.01 ^a^
		Water	0.50±0.02 ^d^	1.25±0.04 ^d^	58.83±0.74 ^c^	0.10±0.01 ^d^	na
*A. santolinoides*	Aerial parts	EA	2.02±0.18 ^c^	6.76±0.77 ^a^	73.00±4.87 ^a^	0.30±0.01 ^c^	0.74±0.02 ^a^
		MeOH	2.32±0.23 ^bc^	4.74±0.41 ^b^	69.02±0.86 ^a^	0.35±0.01 ^b^	0.66±0.08 ^ab^
		Water	0.55±0.04 ^d^	na	40.32±1.40 ^b^	0.04±0.01 ^f^	na
	Roots	EA	2.61±0.04 ^ab^	6.70±0.72 ^a^	66.99±1.98 ^a^	0.40±0.01 ^a^	0.60±0.01 ^b^
		MeOH	2.83±0.32 ^a^	3.28±0.17 ^c^	72.60±0.34 ^a^	0.19±0.01 ^d^	0.38±0.07 ^c^
		Water	0.70±0.07 ^d^	0.78±0.02 ^d^	39.23±0.78 ^b^	0.10±0.01 ^e^	na

* Values are reported as mean ± SD. EA: Ethyl acetate; MeOH: Methanol; GALAE: Galatamine equivalent; KAE: Kojic acid equivalent; ACAE: Acarbose equivalent; na: not active. Different letters in same column indicate significant differences for each *Achillea* species (*p* < 0.05).

## Data Availability

Data is contained within the article and Supplementary Materials.

## Supplementary Materials

The following are available online at https://www.mdpi.com/article/10.3390/antiox10081180/s1, Table S1. Percentages of explained variances, eigenvalues and contribution of metabolites on the first six components of PCA. Table S2. Percentages of explained variances, eigenvalues and contribution of biological activities on the first three components of PCA. Table S3. Upregulated and downreagulated mRNA by the six sesquiterpene lactones and some phenolic compounds. Figure S1: Extracted ion chromatogram of hydroxybenzoic and hydroxycinamic acids and derivatives in negative ion mode of methanolic extracts from Achillea wilhemsii aerial parts (A), A. allepica aerial parts (B), A. wilhemsii roots (C), A. allepica roots (D) (for the compound numbers see Table 2). Figure S2: Extracted ion chromatogram of acylquinic acids in negative ion mode of methanolic extracts from Achillea wilhemsii aerial parts (A), A. allepica aerial parts (B), A. wilhemsii roots (C), A. allepica roots (D) (for the compound numbers see Table 2). Figure S3: Extracted ion chromatogram of flavonoid glycosides in negative ion mode of methanolic extracts from Achillea wilhemsii aerial parts (A), A. allepica aerial parts (B), A. wilhemsii roots (C), A. allepica roots (D) (for the compound numbers see Table 2). Figure S4: Extracted ion chromatogram of flavonoid aglycones in negative ion mode of methanolic extracts from Achillea wilhemsii aerial parts (A), A. allepica aerial parts (B), A. wilhemsii roots (C), A. allepica roots (D) (for the compound numbers see Table 2). Figure S5: MS/MS spectra of hydroxybenzoic, hydroxycinnamic acids and their glycosides, and sugar esters (for the compound numbers see Table 2). Figure S6: MS/MS spectra of acylquinic acids (for the compound numbers see Table 2). Figure S7: MS/MS spectra of C-, C,O- and O-flavonoid glycosides(for the compound numbers see Table 2). Figure S8: MS/MS spectra of 6 methoxyflavonoids (aglycones and glycosides) (for the compound numbers see Table 2). Figure S9: MS/MS spectra of sesquiterpene lactones (for the compound numbers see Table 2).
