# Supplementary material for: UHPLC-MS Characterization and Biological Insights of Different Solvent Extracts of Two Achillea Species (A. aleppica and A. santolinoides) from Turkey
Source: Antioxidants (Basel). 2021 Jul 24;10(8):1180. doi: 10.3390/antiox10081180 (PMC8388973; doi:10.3390/antiox10081180)
Supplement: Supplementary file 1 [file antioxidants-10-01180-s001.zip › antioxidants-1294516-supplementary.pdf]

Table S1. Percentages of explained variances, eigenvalues and contribution of metabolites on the first six components of PCA

	PC1	PC2	PC3	PC4	PC5	PC6
Percentages of explained variances (%)	35.34	18.36	09.20	07.57	06.51	05.99
Eigenvalues	33.57	17.44	8.74	7.19	6.19	5.69
C1	1,692	0,385	1,350	1,405	0,003	1,166
C2	1,979	0,509	0,120	0,587	0,101	1,164
C3	0,488	2,214	2,385	1,805	1,204	0,450
C4	0,000	0,691	2,960	2,810	3,147*	0,851
C5	1,938	1,202	0,054	0,980	0,000	0,118
C6	0,121	1,116	2,810	0,768	1,308	2,012
C7	1,181	0,972	1,875	1,487	0,008	1,268
C8	2,449*	0,290	0,008	0,165	0,001	0,337
C9	2,142*	0,003	0,476	0,220	2,753	0,002
C10	2,018*	0,172	1,256	0,065	0,224	2,026
C11	0,976	0,017	1,765	0,054	1,131	6,291*
C12	2,077*	0,373	0,208	2,137	0,020	0,175
C13	2,436*	0,278	0,008	0,107	0,001	0,260
C14	0,100	0,481	1,080	0,000	0,152	6,746*
C15	2,713*	0,000	0,346	0,016	0,004	0,001
C16	0,501	0,006	0,991	1,487	3,088*	1,250
C17	1,978	1,033	0,056	0,011	0,424	0,421
C18	0,007	0,258	2,070	0,000	2,358	8,445*
C19	1,361	0,145	0,022	0,374	0,137	1,936
C20	0,533	2,020	0,082	0,087	4,414*	0,982
C21	0,418	0,066	0,288	0,291	1,736	0,697
C22	0,059	0,270	2,694	0,031	1,664	7,865*
C23	0,935	0,837	0,043	0,166	3,077*	4,430*
C24	1,536	0,712	0,420	0,585	0,347	0,017
C24a	0,430	2,553	0,159	0,949	0,593	2,427
C25	1,976	0,469	0,278	2,340	0,013	0,187
C26	0,164	0,046	0,141	2,641	0,253	0,308
C27	1,104	0,126	1,217	4,615*	0,737	0,096
C28	2,116*	0,349	0,112	2,154	0,004	0,101

C29	0,128	2,750	2,436	0,776	0,431	1,758
C30	1,154	0,108	1,826	0,029	0,756	5,477*
C31	0,592	0,328	1,746	1,832	0,440	0,113
C32	2,197*	0,030	0,258	0,652	1,233	0,096
C33	1,450	1,979	0,021	1,504	0,011	0,046
C34	2,173*	0,311	0,050	2,453	0,038	0,001
C35	0,780	0,995	1,071	3,148*	0,264	0,212
C36	1,636	1,351	0,395	0,309	0,002	0,117
C37	2,356*	0,520	0,152	1,217	0,001	0,005
C38	2,269*	0,115	0,071	1,016	0,574	0,061
C39	1,300	0,046	0,274	0,174	0,807	7,088*
C40	0,451	2,422	0,014	1,101	1,953	0,848
C41	2,027*	0,181	0,258	1,816	0,204	0,188
C42	1,590	0,030	0,085	1,737	1,477	0,934
C43	1,709	0,530	0,022	1,403	2,198	0,136
C44	2,225*	0,042	0,274	0,711	0,940	0,113
C45	0,263	0,000	5,301*	1,071	0,004	0,334
C46	1,443	0,172	0,310	1,439	0,659	0,021
C47	1,664	1,063	0,731	0,246	1,779	0,643
C48	2,080*	0,324	0,297	2,124	0,021	0,186
C49	1,551	1,675	0,125	1,160	0,033	0,086
C50	0,582	0,863	0,414	0,787	0,894	0,000
C51	1,227	1,423	0,185	3,565*	0,000	0,225
C52	1,228	0,063	2,604	0,937	0,166	2,932
C53	2,163*	0,715	1,173	0,161	0,453	0,015
C54	1,232	0,063	2,599	0,927	0,169	2,919
C55	2,566*	0,095	0,260	0,944	0,005	0,016
C56	1,115	1,591	0,098	3,509*	0,004	0,496
C57	0,835	2,026	0,580	1,003	0,231	0,389
C58	0,050	0,285	1,739	1,241	0,200	0,730
C59	0,805	0,000	0,891	0,517	5,450*	0,912
C60	1,368	0,284	2,185	0,028	0,192	2,268
C61	0,482	0,225	1,216	0,689	3,934*	0,143
C62	1,403	0,436	0,291	0,999	1,492	0,346
C63	1,177	0,207	0,454	1,693	0,632	1,063
C64	0,888	1,066	0,398	0,128	0,026	0,763

C65	1,315	0,584	0,011	0,051	1,485	0,019
C66	1,431	0,126	1,573	1,459	2,667	0,772
C67	2,398*	0,161	0,079	0,685	0,500	0,137
C68	0,482	0,225	1,216	0,689	3,934*	0,143
C69	2,258*	0,019	0,248	0,251	2,399	0,061
C70	1,154	0,000	2,850	0,137	0,817	0,657
C71	0,427	3,487*	0,354	0,522	0,316	0,142
C72	0,487	3,525*	0,120	1,122	1,118	0,105
C73	0,074	3,567*	1,200	2,115	0,246	0,319
C74	0,018	3,924*	0,231	1,375	0,449	0,072
C75	0,000	1,146	0,463	1,782	6,604*	1,032
C76	0,505	0,816	5,034*	0,990	0,038	0,266
C77	0,138	4,539*	0,205	1,380	0,025	0,073
C78	0,138	4,539*	0,205	1,380	0,025	0,073
C79	0,010	4,055*	0,000	0,272	0,200	0,513
C80	0,092	2,289	2,427	0,010	0,643	0,280
C81	0,051	5,382*	0,282	0,128	0,009	0,000
C82	0,148	3,881*	0,544	1,035	0,271	0,135
C83	0,132	2,348	0,637	0,966	1,521	3,334*
C84	0,580	2,398	0,450	0,308	0,990	0,640
C85	0,406	0,094	6,499*	2,050	0,675	0,280
C86	0,156	2,002	0,087	0,125	2,638	2,649
C87	0,006	1,275	7,142*	0,642	0,553	0,059
C88	0,269	0,701	0,863	0,072	0,006	0,600
C89	0,064	2,451	0,406	3,814*	1,833	0,305
C90	0,007	0,849	7,117*	0,058	0,028	0,543
C91	0,046	1,759	1,572	0,065	5,204*	2,311
C92	0,962	0,051	1,133	1,239	4,161*	0,081
C93	1,337	0,997	0,502	1,829	2,056	0,334
C94	1,321	0,898	0,472	2,065	2,009	0,356

Table S2. Percentages of explained variances, eigenvalues and contribution of biological activities on the first three components of PCA

		PC1	PC2	PC2
Percentages of explained variances (%)		**63.14**	**15.12**	**10.25**
Eigenvalue		**6.94**	**1.66**	**1.12**
Biological activities	**DPPH**	11,588*	6,255	1,772
	**ABTS**	12,548*	6,207	0,289
	**CUPRAC**	11,543*	8,557	2,165
	**FRAP**	11,989*	7,330	2,554
	**MCA**	0,019	28,044*	30,267*
	**PBD**	2,771	0,172	56,958*
	**AChE**	8,604	17,109*	4,327
	**BChE**	12,371*	0,039	0,533
	**Tyrosinase**	5,814	17,364*	0,975
	**Amylase**	12,531*	2,574	0,130
	**Glucosidase**	10,221*	6,350	0,028

*Biological activities having highest contribution scores on principal components

Table S3. Upregulated and downreagulated mRNA by the six sesquiterpene lactones and some phenolic compounds

	Artabsin	Dehydroleucodin	Dihydrosantamarin	Leucodin	Matricin	Tanaparthin peroxide	Neochlorogenic	Chlorogenic acid	Homoorientin	Vitexin	Isovitexin
Upregulated mRNA	BRMS1<br>SFRP1<br>MAP7<br>CYP3A4<br>HOXA11<br>APPBP2<br>CASP2<br>ALDOA<br>GNMT<br>PFKFB2<br>SH3BGRL<br>TRIM13<br>NUCB2<br>FOXO4<br>SCGB1D2<br>DNAJB14<br>GAL<br>PPP1CB<br>CHEK1<br>FADS1<br>TACC1<br>CACNG4<br>CHRNE<br>CISH<br>EPB41L3<br>PLK3<br>P2RY14<br>MYT1<br>KCNH2<br>PLLP<br>SLC25A4<br>ZYX<br>SFTPB<br>NRTN<br>WISP2<br>ENC1<br>MT1L<br>WT1<br>HMGCR<br>COL22A1<br>KLK13<br>ITGA6	PPP1CB<br>DNAJB14<br>FOXO1<br>CACNG4<br>CHRNE<br>PFKFB2<br>SH3BGRL<br>TRIM13<br>COL22A1<br>KLK13<br>CNN2<br>DYSF<br>FAM110C<br>MYO5B<br>PPFIBP2<br>LGALS9<br>KCNH2<br>PLLP<br>SLC25A4<br>ZYX<br>TMEM56<br>FOXO4<br>CREB3L1<br>RNASEL<br>PDZD2<br>IL4R<br>KLK7<br>PLXNA2<br>SERPINB8<br>ABAT<br>ERMP1<br>MSX2<br>EPB41L3<br>TRIOBP<br>WT1<br>SQLE<br>ITGAM<br>CD68<br>NUCB2<br>NUDT11<br>FAM49A<br>SLC4A11<br>SLC45A2<br>GNMT<br>C8ORF4<br>FOXC1	MYT1<br>PFKFB2<br>SH3BGRL<br>TRIM13<br>CACNG4<br>CHRNE<br>PPP1CB<br>DNAJB14<br>CREB3L1<br>RNASEL<br>KCNH2<br>PLLP<br>SLC25A4<br>ZYX<br>LGALS9<br>CNN2<br>DYSF<br>FAM110C<br>MYO5B<br>PPFIBP2<br>AURKA<br>TMEM56<br>HOXA11<br>IL4R<br>HSPCB<br>PBK<br>WISP2<br>FOXO4<br>SERPINB8<br>COL22A1<br>KLK13<br>TMEM38B<br>KRT6A<br>WT1<br>NUDT11<br>GAS6<br>CD68<br>CDC20<br>UGT2B15<br>ABCC13<br>AURKB<br>CLIC6<br>CSRP2BP<br>CTPS1<br>DEPDC1B<br>PCP4	PPP1CB<br>DNAJB14<br>FOXO4<br>FOXO1<br>PFKFB2<br>SH3BGRL<br>TRIM13<br>WT1<br>COL22A1<br>KLK13<br>CACNG4<br>CHRNE<br>CNN2<br>DYSF<br>FAM110C<br>MYO5B<br>PPFIBP2<br>CYP2B6<br>LGALS9<br>CYP3A4<br>KCNH2<br>PLLP<br>SLC25A4<br>ZYX<br>PDZD2<br>TMEM56<br>HOXA11<br>ALDOA<br>CREB3L1<br>RNASEL<br>MAP7<br>MYT1<br>SERPINB8<br>BRMS1<br>EPB41L3<br>P2RY14<br>IL4R<br>PLXNA2<br>KLK7<br>S100A9<br>PBK<br>SLC4A11<br>GAL<br>MAP3K8<br>MT1L<br>ABAT	AURKA<br>MAP7<br>WT1<br>CYP3A4<br>COL22A1<br>KLK13<br>PFKFB2<br>SH3BGRL<br>TRIM13<br>HMGCR<br>FOXO4<br>CACNG4<br>CHRNE<br>HOXA11<br>BRMS1<br>S100A9<br>PPP1CB<br>FADS1<br>DNAJB14<br>CYP2B6<br>PLXNA2<br>SLC45A2<br>KCNH2<br>PLLP<br>SLC25A4<br>ZYX<br>SQLE<br>LGALS9<br>CNN2<br>DYSF<br>FAM110C<br>MYO5B<br>PPFIBP2<br>APPBP2<br>FAM49A<br>PBK<br>PCDH17<br>CAMKK2<br>TMEM56<br>CREB3L1<br>RNASEL<br>GNMT<br>ABAT<br>ERMP1<br>MSX2<br>SERPINB8	SQLE<br>IL4R<br>PLXNA2<br>SERPINB8<br>FAM49A<br>CD68<br>BRMS1<br>MAP7<br>NUDT11<br>PFKFB2<br>SH3BGRL<br>TRIM13<br>CACNG4<br>CHRNE<br>PPP1CB<br>NUCB2<br>DNAJB14<br>APPBP2<br>GNMT<br>KCNH2<br>PLLP<br>SLC25A4<br>ZYX<br>FADS1<br>CAMKK2<br>CREB3L1<br>RNASEL<br>WT1<br>SLC45A2<br>COL22A1<br>KLK13<br>EPB41L3<br>CNN2<br>DYSF<br>FAM110C<br>MYO5B<br>PPFIBP2<br>FOXO4<br>LGALS9<br>SFRP1<br>BCAS1<br>TMEM56<br>CHEK1<br>HMGCR<br>TNNT1<br>SFTPB	TCF12<br>SMPD1<br>VASP<br>OGG1<br>CASP2<br>FTL<br>FGFR1<br>PCDH17<br>ERBB2<br>COL22A1<br>KLK13<br>LGALS9<br>WT1<br>LST1<br>S100A9<br>GAS6<br>HMGCR<br>FOXO1<br>PLA2G6<br>ITGA6<br>MT1L<br>MAP7<br>AURKA	TCF12<br>SMPD1<br>VASP<br>OGG1<br>CASP2<br>FTL<br>FGFR1<br>PCDH17<br>ERBB2<br>COL22A1<br>KLK13<br>LGALS9<br>WT1<br>LST1<br>S100A9<br>GAS6<br>HMGCR<br>FOXO1<br>PLA2G6<br>ITGA6<br>MT1L<br>MAP7<br>AURKA	CEP72<br>TCF12<br>DSTN<br>NOTCH1<br>RRM2B<br>KDELC1<br>PGM2<br>FAM171A1<br>HSPA12A<br>LIM2<br>MAP3K7IP3<br>NKAIN1<br>PQLC3<br>RPPH1<br>SEMA3G<br>SLC37A2<br>STBD1<br>TMEM2<br>TRIM21<br>WNT11<br>ZIC2<br>TMEM38B<br>HES1<br>CD151<br>ELOVL1<br>GAL3ST1<br>GCOM1<br>KAT2B<br>PLEKHM1<br>SLC20A1<br>CMBL<br>CTTN<br>SLC4A11<br>ANKRA2<br>IGDCC4	CEP72<br>TCF12<br>DSTN<br>RRM2B<br>TMEM38B<br>KDELC1<br>PGM2<br>NOTCH1<br>HES1<br>FAM171A1<br>HSPA12A<br>LIM2<br>MAP3K7IP3<br>NKAIN1<br>PQLC3<br>RPPH1<br>SEMA3G<br>SLC37A2<br>STBD1<br>TMEM2<br>TRIM21<br>WNT11<br>ZIC2<br>SLC20A1<br>CMBL<br>CD151<br>ELOVL1<br>GAL3ST1<br>GCOM1<br>KAT2B<br>PLEKHM1<br>BLM<br>SLC4A11<br>CTTN<br>ANKRA2	CEP72<br>TCF12<br>DSTN<br>KDELC1<br>PGM2<br>TMEM38B<br>NOTCH1<br>RRM2B<br>HES1<br>FAM171A1<br>HSPA12A<br>LIM2<br>MAP3K7IP3<br>NKAIN1<br>PQLC3<br>RPPH1<br>SEMA3G<br>SLC37A2<br>STBD1<br>TMEM2<br>TRIM21<br>WNT11<br>ZIC2<br>CD151<br>ELOVL1<br>GAL3ST1<br>GCOM1<br>KAT2B<br>PLEKHM1<br>SLC20A1<br>CMBL<br>CTTN<br>GFRA1<br>ANKRA2<br>IGDCC4<br>THAP10

		APPBP2<br>MAP3K8<br>BMPR1B<br>CYP2S1<br>GBP1<br>SFTPB<br>PSAP<br>MAP7<br>FADS1<br>S100A9<br>BRMS1<br>ITGA6<br>TSKU<br>ALDOA<br>CA2<br>ENC1	PTGER3<br>SAC3D1<br>SFXN2<br>SLC39A8<br>FOXC1<br>EPB41L3<br>SLC45A2<br>TRIOBP<br>MCM6<br>MCM7<br>KLK7<br>TK1<br>ERBB2<br>C8ORF4<br>CYP2S1<br>SCGB2A1<br>BLM<br>BMPR1B<br>KIAA0101<br>GMNN<br>PLK4<br>GFRA1<br>NUCB2<br>GAL<br>SLC4A11<br>FANCI<br>TRIP13<br>ARL3<br>CENPA<br>FBXO5<br>GINS2<br>KIF11<br>KIF15<br>NCAPD2<br>PLOD1<br>PRIM1<br>TACC3<br>UBE2T<br>TSKU<br>FADS1<br>ATAD2<br>CENPF<br>HMGB2<br>KIF20A<br>KIF4A<br>SMC2<br>APPBP2<br>ECT2<br>IGFBP2<br>HES2	ERMP1<br>MSX2<br>SFRP1<br>C8ORF4<br>ENC1<br>FOXC1<br>HMGCR<br>CYP2S1<br>EXOSC4<br>ITGA6<br>KRT6A<br>FADS1<br>AURKA	IL4R<br>SFRP1<br>FOXO1<br>EPB41L3<br>ABCA1<br>PDZD2<br>NUCB2<br>CDC20<br>SLC4A11<br>MT1L	CDC42EP3<br>KLK2<br>TRIOBP<br>ABAT<br>ERMP1<br>MSX2<br>PDZD2<br>FOXO1<br>HES2<br>KLK7<br>SCGB1D2<br>CISH<br>GAL<br>SLC4A11<br>HOXA11<br>NRTN<br>ALDOA<br>FOXC1<br>ENC1<br>ITGA6			THAP10<br>C1ORF6<br>3<br>BLM<br>MOD2<br>PFKFB2<br>SH3BGR<br>L<br>TRIM13<br>GAS6<br>OGG1<br>FDXR<br>AHRR<br>SFRP1<br>RPS6KA<br>3<br>GALR2<br>UBE3B<br>COX5B<br>FAM46B<br>S100A13<br>RGL1<br>SLC39A<br>4<br>GFRA1<br>ETFB<br>PLK3<br>SMPD1<br>CYP2S1<br>TRIOBP<br>CDT1<br>MASTL<br>MCM10<br>IER5L<br>ERBB2<br>ABCC13<br>AURKB<br>CLIC6<br>CSRP2B<br>P<br>CTPS1<br>DEPDC1<br>B<br>PCP4<br>PTGER3<br>SAC3D1<br>SFXN2<br>SLC39A<br>8<br>SH3RF1	IGDCC4<br>THAP10<br>GFRA1<br>C1ORF6<br>3<br>OGG1<br>CDT1<br>MASTL<br>MCM10<br>MOD2<br>ABCC13<br>AURKB<br>CLIC6<br>CSRP2B<br>P<br>CTPS1<br>DEPDC1<br>B<br>PCP4<br>PTGER3<br>SAC3D1<br>SFXN2<br>SLC39A<br>8<br>MCM6<br>MCM7<br>PPP1R14<br>C<br>COX5B<br>UBE3B<br>GMNN<br>PLK4<br>GAS6<br>AHRR<br>TRIOBP<br>RNFT2<br>FAM122<br>A<br>FDXR<br>TRIP13<br>RPS6KA<br>3<br>CYP2S1<br>GALR2<br>RACGA<br>P1<br>ERBB2<br>FAM46B<br>S100A13<br>PFKFB2	SLC4A1<br>1<br>BLM<br>C1ORF6<br>3<br>SMPD1<br>EID1<br>MOD2<br>FDXR<br>OGG1<br>AHRR<br>PFKFB2<br>SH3BGR<br>L<br>TRIM13<br>RPS6KA<br>3<br>GAS6<br>ABCC13<br>AURKB<br>CLIC6<br>CSRP2B<br>P<br>CTPS1<br>DEPDC1<br>B<br>PCP4<br>PTGER3<br>SAC3D1<br>SFXN2<br>SLC39A<br>8<br>UBE3B<br>FAM122<br>A<br>COX5B<br>GALR2<br>FAM46B<br>S100A13<br>TRIP13<br>CDT1<br>MASTL<br>MCM10<br>RNFT2<br>PLK3<br>SFRP1<br>RACGA<br>P1<br>ARL3<br>CENPA

			TMEM97<br>RACGAP1<br>GNMT<br>CMBL<br>SFRP1<br>MITF<br>CAMKK2<br>PPP2R2C<br>MAP7<br>MYBL2<br>SLC6A14<br>CXCL11<br>PPARGC1A<br>FOXO1<br>ALOX15B<br>AMACR<br>RNF103<br>OLFM1<br>CHEK1<br>PDZD2<br>S100A9<br>UGT2B7<br>CYP3A4<br>SLC2A4<br>HPGD<br>CKS1B<br>GBP1<br>CDC42EP3<br>ITPK1<br>ID1<br>TMPRSS2<br>BCAS1<br>PPL<br>SAA2<br>SQLE<br>MT1L<br>KLK2<br>H6PD<br>HSD11B1<br>ALPK3<br>HHIPL2<br>STAT5A<br>PSMD6<br>DENND5A<br>MPHOSPH9<br>PLXNA2<br>CDT1<br>MASTL<br>MCM10<br>TBC1D1						TSKU<br>HIVEP1<br>PBK<br>TRIP13<br>ARL3<br>CENPA<br>FBXO5<br>GINS2<br>KIF11<br>KIF15<br>NCAPD2<br>PLOD1<br>PRIM1<br>TACC3<br>UBE2T<br>RACGAP1<br>RNFT2<br>ECT2<br>VNN1<br>CASP2<br>ITGA6	SH3BGRL<br>TRIM13<br>ARL3<br>CENPA<br>FBXO5<br>GINS2<br>KIF11<br>KIF15<br>NCAPD2<br>PLOD1<br>PRIM1<br>TACC3<br>UBE2T<br>RGL1<br>SLC39A4<br>ETFB<br>ECT2<br>TSKU<br>PBK<br>CBFA2T3<br>KCNJ5<br>EID1<br>PLK3<br>CBR1<br>IGFBP2<br>CHEK1<br>NRTN<br>IER5L<br>MLPH<br>HIVEP1<br>PON1<br>SFRP1<br>MSMB<br>ADAMTS9<br>SH3RF1<br>PGR<br>ITGA6<br>CKS1B<br>CDC20<br>SMPD1<br>CA2<br>CYTH1<br>VNN1<br>AURKA	FBXO5<br>GINS2<br>KIF11<br>KIF15<br>NCAPD2<br>PLOD1<br>PRIM1<br>TACC3<br>UBE2T<br>TSKU<br>CYP2S1<br>ERBB2<br>PBK<br>ETFB<br>RGL1<br>SLC39A4<br>ECT2<br>TRIOBP<br>CBR1<br>MCM6<br>MCM7<br>CYTH1<br>WISP2<br>PPP1R14C<br>IER5L<br>GMNN<br>PLK4<br>SH3RF1<br>HIVEP1<br>PGR<br>NRTN<br>CKS1B<br>CDC20<br>VNN1

			CA2<br>ST3GAL1<br>GPRC5A<br>OLFML3<br>DLX4<br>RPS6KA3<br>TACC1<br>ABAT<br>ERMP1<br>MSX2<br>CENPN<br>PDS5B<br>SOCS2<br>SHBG<br>PTGER4<br>SFTPB<br>SCGB1D2<br>SCNN1A<br>HMMR<br>BRMS1<br>CITED2<br>FAM49A<br>UBE2C<br>TFF3<br>ITGAM<br>GGH<br>STMN1								
Downreagulated mRNA	NSF<br>SESN1<br>HIG2<br>SLTM<br>ZBTB43<br>MYNN<br>ADORA2B<br>CDCA4<br>CENPJ<br>DNAJC9<br>DONSON<br>HAUS8<br>MCMBP<br>NUP155<br>PARP2<br>RMI1<br>SCARA3<br>SLC16A14<br>SUV39H2<br>TPM1<br>CAB39<br>MBNL2<br>TMSB15A	TMSB15A<br>MYNN<br>C9ORF40<br>ADORA2B<br>CDCA4<br>CENPJ<br>DNAJC9<br>DONSON<br>HAUS8<br>MCMBP<br>NUP155<br>PARP2<br>RMI1<br>SCARA3<br>SLC16A14<br>SUV39H2<br>TMEM194A<br>PPIH<br>CTSL2<br>GPR19<br>LFNG<br>MIR20B<br>NUP54	ADORA2B<br>CDCA4<br>CENPJ<br>DNAJC9<br>DONSON<br>HAUS8<br>MCMBP<br>NUP155<br>PARP2<br>RMI1<br>SCARA3<br>SLC16A14<br>SUV39H2<br>TMSB15A<br>MYNN<br>C9ORF40<br>TMEM194A<br>MAST4<br>MSH5<br>TREX1<br>CTSL2<br>GPR19<br>LFNG	MYNN<br>TMSB15A<br>NOP56<br>ADORA2B<br>CDCA4<br>CENPJ<br>DNAJC9<br>DONSON<br>HAUS8<br>MCMBP<br>NUP155<br>PARP2<br>RMI1<br>SCARA3<br>SLC16A14<br>SUV39H2<br>C9ORF40<br>PPIH<br>HIG2<br>TMEM194A<br>LBR<br>SLTM<br>ZBTB43	SESN1<br>MYNN<br>HIG2<br>SLTM<br>ZBTB43<br>AKR1C3<br>TRAF5<br>ADORA2B<br>CDCA4<br>CENPJ<br>DNAJC9<br>DONSON<br>HAUS8<br>MCMBP<br>NUP155<br>PARP2<br>RMI1<br>SCARA3<br>SLC16A14<br>SUV39H2<br>CCL5<br>TMSB15A<br>CAPRIN1	SESN1<br>GEMIN4<br>RPS19<br>NAP1L1<br>TNPO2<br>NME2<br>ASPH<br>NSF<br>ADORA2B<br>CDCA4<br>CENPJ<br>DNAJC9<br>DONSON<br>HAUS8<br>MCMBP<br>NUP155<br>PARP2<br>RMI1<br>SCARA3<br>SLC16A14<br>SUV39H2<br>MYNN<br>TMSB15A	ADAM19<br>CCL5<br>NOP56<br>CAPRIN1<br>TRAF5<br>SDC4<br>TBL1XR1<br>LGALS1<br>C9ORF40<br>UBE2M<br>FKBP5<br>NPM1<br>CXCL10<br>SAP18<br>TNFRSF10B<br>CCDC93<br>STX1A<br>HK2<br>DEK<br>H1FX<br>HIG2<br>ZFAND5	ADAM19<br>CCL5<br>NOP56<br>CAPRIN1<br>TRAF5<br>SDC4<br>TBL1XR1<br>LGALS1<br>C9ORF40<br>UBE2M<br>FKBP5<br>NPM1<br>CXCL10<br>SAP18<br>TNFRSF10B<br>CCDC93<br>STX1A<br>HK2<br>DEK<br>H1FX<br>HIG2<br>ZFAND5<br>NAP1L1	DMXL1<br>OLFML1<br>TUBGCP3<br>TBC1D9<br>C9ORF41<br>APTX<br>RSBN1<br>TEFM<br>HIG2<br>MAPK8<br>ADAM19<br>SDC4<br>OSBP2<br>GSS<br>DEK<br>STEAP1<br>MAN1A2<br>YPEL5<br>LRP6	DMXL1<br>OLFML1<br>TUBGCP3<br>TBC1D9<br>C9ORF41<br>APTX<br>RSBN1<br>TEFM<br>HIG2<br>GSS<br>MAPK8<br>LRP6<br>YPEL5<br>SDC4<br>STEAP1<br>OSBP2<br>DEK<br>ADAM19<br>MAN1A2	DMXL1<br>OLFML1<br>TUBGCP3<br>APTX<br>RSBN1<br>TBC1D9<br>C9ORF41<br>TEFM<br>LRP6<br>ADAM19<br>MAPK8<br>HIG2<br>OSBP2<br>SDC4<br>ABCA13<br>ABCC13<br>TMEM59L<br>YPEL5<br>STEAP1

	MAST4<br>MSH5<br>TREX1<br>NOP56<br>PPIH<br>TNPO2<br>RPS19<br>C9ORF40	PRR16<br>SP4<br>MAST4<br>MSH5<br>TREX1<br>CBX2<br>GART<br>LBR<br>SESN1<br>RPS19<br>ASPH<br>TNPO2<br>GEMIN4<br>NAP1L1<br>DEK<br>SLTM<br>ZBTB43<br>HIG2<br>NOP56<br>POC1A<br>EFNB2<br>NME2<br>TANC1<br>NDE1<br>BARD1<br>TSPAN5<br>NPM1<br>TAOK3<br>INCENP<br>GSS<br>CXCR7<br>TRIM16<br>HSPA6<br>TRAF5<br>GPLD1<br>DNMT1<br>CAPRIN1	MIR20B<br>NUP54<br>PRR16<br>SP4<br>GART<br>NOP56<br>CBX2<br>RPS19<br>SEC16A<br>PPIH<br>TBC1D9<br>GEMIN4<br>HPS5<br>SESN1<br>AKR1C3<br>YPEL5<br>ASPH<br>MAPK8<br>HSPA6<br>PTPRO<br>EFNB2<br>PLEC<br>ZBTB10<br>HIG2<br>GPLD1<br>SLC14A1<br>ABCC5<br>SLTM<br>ZBTB43<br>GSS<br>KIAA0256<br>MXD4<br>MYH6<br>TCF25<br>UGT1A3<br>BARD1<br>CD58<br>LBR<br>WNT7A<br>HEATR3<br>L1CAM<br>CDK2AP2<br>NDE1<br>DEK<br>NPM1<br>POC1A<br>ELAC2<br>TANC1<br>TRIM16<br>TPM1	CTSL2<br>GPR19<br>LFNG<br>MIR20B<br>NUP54<br>PRR16<br>SP4<br>SESN1<br>MAST4<br>MSH5<br>TREX1<br>TAOK3<br>CBX2<br>WDR77<br>GART<br>GSS<br>ASPH<br>TRAF5<br>EFNB2<br>RPS19<br>TNPO2<br>HSPA6<br>DEK<br>GEMIN4<br>POC1A<br>GPLD1<br>DIO2<br>SEC16A<br>TANC1<br>CAPRIN1<br>NAP1L1<br>NDE1<br>AKR1C3<br>NPM1<br>DHFR<br>ABCC5<br>TPM1<br>BARD1<br>ERCC2<br>TBC1D9	TAOK3<br>WDR77<br>NAP1L1<br>NSF<br>TNPO2<br>MAST4<br>MSH5<br>TREX1<br>CTSL2<br>GPR19<br>LFNG<br>MIR20B<br>NUP54<br>PRR16<br>SP4<br>TMEM194A<br>C9ORF40<br>RPS19<br>EFNB2<br>GEMIN4<br>CBX2<br>ASPH<br>GART<br>NME2<br>ZBTB10<br>PPIH<br>TSPAN5<br>LBR<br>NDE1<br>TOB1<br>POC1A<br>TANC1<br>BARD1<br>NOP56	MBNL2<br>LBR<br>C9ORF40<br>MAST4<br>MSH5<br>TREX1<br>CXCR7<br>CTSL2<br>GPR19<br>LFNG<br>MIR20B<br>NUP54<br>PRR16<br>SP4<br>PPIH<br>GART<br>CBX2<br>TMEM194A<br>SLTM<br>ZBTB43<br>HIG2<br>ETS1<br>NDE1<br>POC1A<br>ZBTB10<br>TANC1<br>TSPAN5<br>OSR2<br>INCENP<br>EFNB2<br>BARD1<br>TPM1<br>NPM1	NAP1L1<br>GPLD1<br>GART<br>KLF10<br>TOB1<br>SOD1<br>TMEM194A<br>TMSB15A<br>SEC16A<br>WWP2<br>MAPK8<br>CXCL2	GPLD1<br>GART<br>KLF10<br>TOB1<br>SOD1<br>TMEM194A<br>TMSB15A<br>SEC16A<br>WWP2<br>MAPK8<br>CXCL2	ITGAV<br>ARNT<br>CADPS2<br>CCDC41<br>FNIP2<br>GCA<br>GPT2<br>ITPKA<br>KCNJ10<br>KLHL14<br>PSD3<br>SPIN3<br>STARD13<br>ZNF711<br>PGM2L1<br>LBR<br>INTU<br>PAQR3<br>QPCT<br>RNFT1<br>MBNL2<br>HK2<br>ABL1<br>HEATR3<br>AIM1<br>C17ORF75<br>COG6<br>MITD1<br>PLSCR1<br>PRKAB2<br>VPS36<br>H1FX<br>ABCA13<br>ABCC13<br>TMEM59L<br>PCOLCE2<br>SLC30A1<br>ZBTB10<br>FTSJD1<br>GUF1<br>MAP4K4<br>TNFRSF10B<br>RASA1<br>NCOA1	ABCA13<br>ABCC13<br>TMEM59L<br>ITGAV<br>ARNT<br>CADPS2<br>CCDC41<br>FNIP2<br>GCA<br>GPT2<br>ITPKA<br>KCNJ10<br>KLHL14<br>PSD3<br>SPIN3<br>STARD13<br>ZNF711<br>TNFRSF10B<br>PGM2L1<br>MBNL2<br>HEATR3<br>AIM1<br>C17ORF75<br>COG6<br>MITD1<br>PLSCR1<br>PRKAB2<br>VPS36<br>INTU<br>PAQR3<br>QPCT<br>RNFT1<br>LBR<br>NCOA1<br>H1FX<br>PCOLCE2<br>FTSJD1<br>GUF1<br>HK2<br>EFNA3<br>ZBTB10<br>SLC30A1<br>RASA1<br>MAP4K4	GSS<br>MAN1A2<br>DEK<br>MFAP4<br>EFNA3<br>ITGAV<br>HK2<br>TNFRSF10B<br>SYMPK<br>ABL1<br>MBNL2<br>ARNT<br>CADPS2<br>CCDC41<br>FNIP2<br>GCA<br>GPT2<br>ITPKA<br>KCNJ10<br>KLHL14<br>PSD3<br>SPIN3<br>STARD13<br>ZNF711<br>LBR<br>PGM2L1<br>HEATR3<br>AIM1<br>C17ORF75<br>COG6<br>MITD1<br>PLSCR1<br>PRKAB2<br>VPS36<br>INTU<br>PAQR3<br>QPCT<br>RNFT1<br>EVI2B<br>PCOLCE2<br>H1FX<br>PDZK1<br>FTSJD1<br>GUF1<br>MAP4K4

			CXCR7<br>TRAF5<br>RPRM<br>INCENP<br>KLF10<br>ERCC2<br>SOX4<br>WDR77<br>PRIMA1<br>OSBP2<br>DNMT1<br>RAD18<br>SPATA5<br>CAPRIN1<br>NCOA1<br>STEAP1<br>CXCL2<br>TGFB2<br>IL24<br>DSC1<br>TAOK3<br>TNPO2<br>DIO2<br>OSR2<br>NAP1L1<br>HK2<br>HIST2H2BE<br>TSPAN5<br>CCL5<br>ARNT2<br>TRA2A<br>KRT23<br>NME2<br>MBNL2						SH3D19<br>EVI2B<br>PHF14<br>DSPP<br>IVNS1A<br>BP<br>CXCL10<br>AADAT<br>CDKAL1<br>CEP70<br>DOCK4<br>EXOC6<br>TMEM1<br>17<br>ROCK2<br>ESM1<br>RAB20<br>FOXP1<br>NTN4<br>POLR3B<br>PRKD3<br>TTC27<br>WDR7<br>ZHX2<br>ZNRF3<br>FNDC3B<br>AKIRIN<br>1<br>BTBD11<br>COBLL1<br>DAB2<br>NADKD<br>1<br>RBM12<br>EFNA3<br>KRAS<br>NAV2<br>ADORA<br>2B<br>CDCA4<br>CENPJ<br>DNAJC9<br>DONSO<br>N<br>HAUS8<br>MCMBP<br>NUP155<br>PARP2<br>RMI1<br>SCARA3	EVI2B<br>AADAT<br>CDKAL1<br>CEP70<br>DOCK4<br>EXOC6<br>TMEM1<br>17<br>SH3D19<br>MFAP4<br>IL15<br>PHF14<br>CXCL10<br>SYMPK<br>RAB20<br>DIAPH2<br>KRAS<br>IVNS1A<br>BP<br>MCL1<br>C9ORF4<br>0<br>FNDC3B<br>ROCK2<br>FOXP1<br>NTN4<br>POLR3B<br>PRKD3<br>TTC27<br>WDR7<br>ZHX2<br>ZNRF3<br>ESM1<br>SIAH1<br>PDZK1<br>AKIRIN<br>1<br>BTBD11<br>COBLL1<br>DAB2<br>NADKD<br>1<br>NAV2<br>RBM12<br>KIAA02<br>56<br>MXD4<br>MYH6<br>TCF25<br>UGT1A3	SH3D19<br>RASA1<br>DSPP<br>NCOA1<br>ZBTB10<br>SLC30A<br>1<br>CXCL10<br>IL15<br>RAB20<br>PHF14<br>AADAT<br>CDKAL1<br>CEP70<br>DOCK4<br>EXOC6<br>TMEM1<br>17<br>FOXP1<br>NTN4<br>POLR3B<br>PRKD3<br>TTC27<br>WDR7<br>ZHX2<br>ZNRF3<br>ESM1<br>DIAPH2<br>ROCK2<br>IVNS1A<br>BP<br>FNDC3B<br>KRAS<br>AKIRIN<br>1<br>BTBD11<br>COBLL1<br>DAB2<br>NADKD<br>1<br>NAV2<br>MCL1<br>HPS5<br>DNMT1<br>C9ORF4<br>0<br>UBE2M<br>PACSIN<br>2<br>RBM12

									SLC16A14<br>SUV39H2<br>SIAH1<br>CUL4B<br>MFAP4<br>TAOK3<br>DNMT1<br>MTIF2<br>PACSIN2<br>HSPA5<br>AHRR<br>CLASP2<br>CREB3L2<br>IGF2BP3<br>PDGFC<br>RAB28<br>RALA<br>SNX24<br>TMEM139<br>MCL1<br>C9ORF40<br>SYMPK<br>CD74<br>IL1R1<br>BGLAP<br>CHST15<br>DRG2<br>FOXN3<br>IL15<br>WWC2<br>HPS5<br>KIAA0256<br>MXD4<br>MYH6<br>TCF25<br>UGT1A3<br>DIAPH2<br>RARB<br>PDZK1<br>TRAF5<br>TMEM121<br>EFNB2	CYP1B1<br>WWC2<br>DNMT1<br>TMEM121<br>CUL4B<br>MTIF2<br>DSPP<br>TAOK3<br>PACSIN2<br>HPS5<br>WNT7A<br>CDK2AP2<br>FOXN3<br>AHRR<br>CLASP2<br>CREB3L2<br>IGF2BP3<br>PDGFC<br>RAB28<br>RALA<br>SNX24<br>TMEM139<br>HSPA5<br>MSI2<br>ADORA2B<br>CDCA4<br>CENPJ<br>DNAJC9<br>DONSON<br>HAUS8<br>MCMBP<br>NUP155<br>PARP2<br>RMI1<br>SCARA3<br>SLC16A14<br>SUV39H2<br>DRG2<br>OCLN<br>IL1R1<br>CD74	ADORA2B<br>CDCA4<br>CENPJ<br>DNAJC9<br>DONSON<br>HAUS8<br>MCMBP<br>NUP155<br>PARP2<br>RMI1<br>SCARA3<br>SLC16A14<br>SUV39H2<br>SIAH1<br>KIAA0256<br>MXD4<br>MYH6<br>TCF25<br>UGT1A3<br>MTIF2<br>CUL4B<br>HSPA5<br>AHRR<br>CLASP2<br>CREB3L2<br>IGF2BP3<br>PDGFC<br>RAB28<br>RALA<br>SNX24<br>TMEM139<br>MSI2<br>TMEM121<br>CDK2AP2<br>TAOK3<br>WWC2<br>TRAF5<br>CD74<br>IL1R1<br>DRG2<br>BGLAP

									PDGFRL<br>TANC1<br>CDK2AP2<br>RPS19<br>CAPRIN1<br>TOB1	UBE2M<br>BGLAP<br>CHST15<br>SRCRB4D<br>HP<br>RARB<br>EFNB2<br>TRAF5<br>PDGFRL<br>ASAH1<br>ZFAND5<br>RPS19<br>WDR4<br>DIO2<br>CAPRIN1<br>PLEC<br>TANC1<br>TOB1	WNT7A<br>FOXN3<br>CAPRIN1<br>CHST15<br>RARB<br>EFNB2<br>SRCRB4D<br>TOB1<br>PDGFRL<br>TANC1<br>WDR4<br>RPS19<br>PLEC

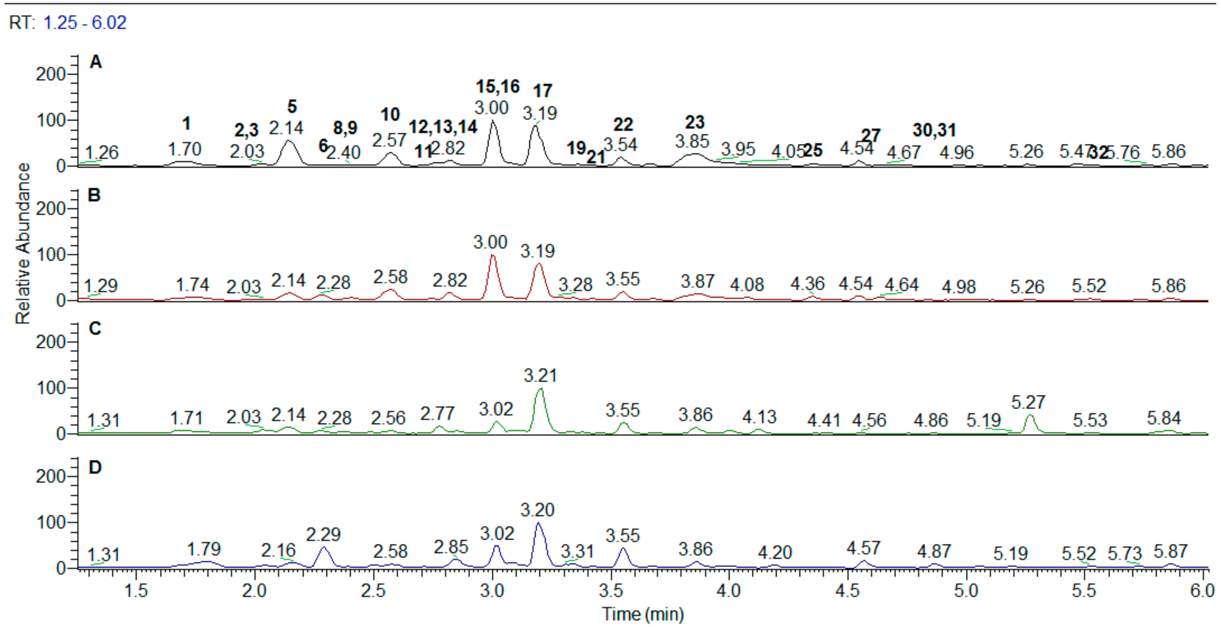

Figure S1. Extracted ion chromatogram of hydroxybenzoic and hydroxycinamic acids and derivatives in negative ion mode of methanolic extracts from *Achillea wilhemsii* aerial parts (A), *A. allepica* aerial parts (B), *A. wilhemsii* roots (C), *A. allepica* roots (D) (for the compound numbers see Table 2).

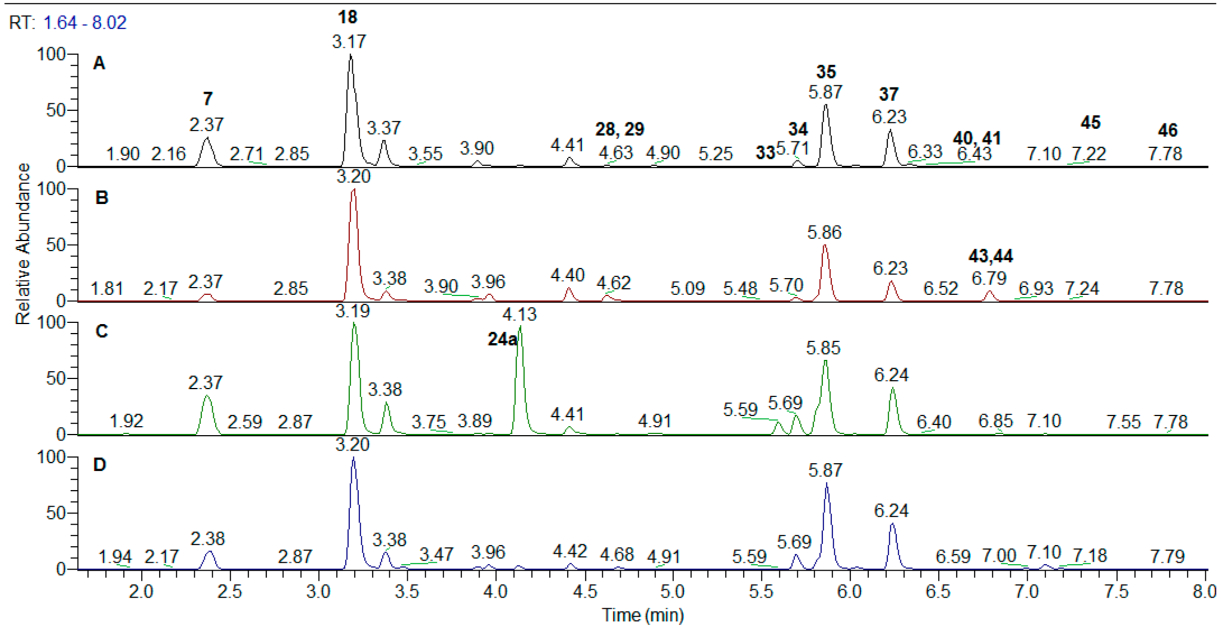

Figure S2. Extracted ion chromatogram of acylquinic acids in negative ion mode of methanolic extracts from *Achillea wilhemsii* aerial parts (A), *A. allepica* aerial parts (B), *A. wilhemsii* roots (C), *A. allepica* roots (D) (for the compound numbers see Table 2).

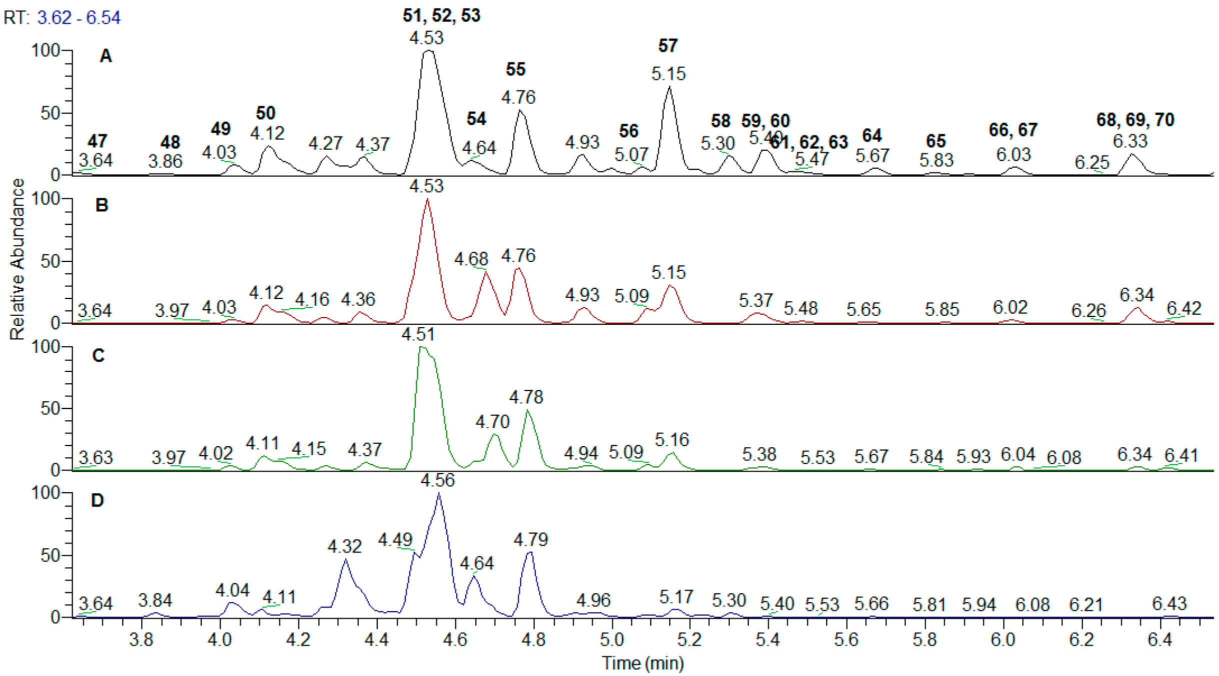

Figure S3. Extracted ion chromatogram of flavonoid glycosides in negative ion mode of methanolic extracts from *Achillea wilhemsii* aerial parts (A), *A. allepica* aerial parts (B), *A. wilhemsii* roots (C), *A. allepica* roots (D) (for the compound numbers see Table 2).

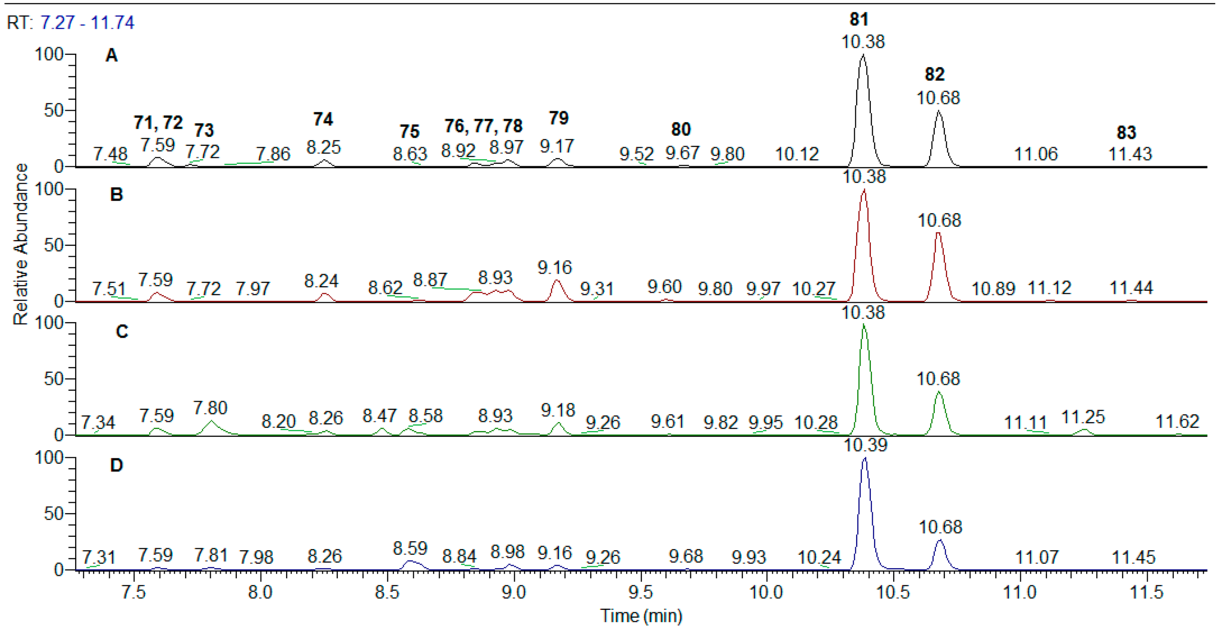

Figure S4. Extracted ion chromatogram of flavonoid aglycones in negative ion mode of methanolic extracts from *Achillea wilhemsii* aerial parts (A), *A. allepica* aerial parts (B), *A. wilhemsii* roots (C), *A. allepica* roots (D) (for the compound numbers see Table 2).

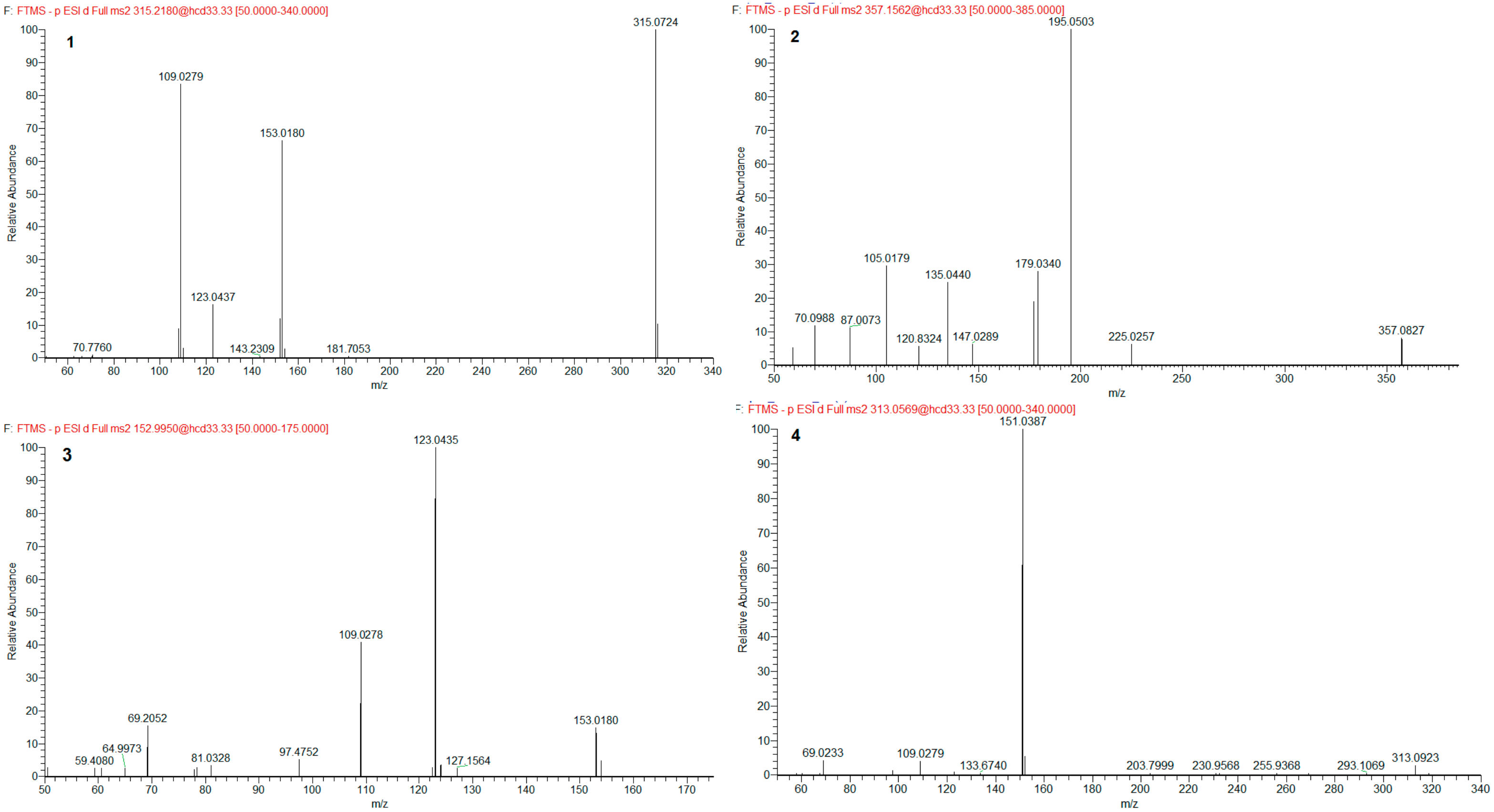
F: FTMS - p ESI d Full ms2 315.2180@hcd33.33 [50.0000-340.0000]
1
315.0724
109.0279
153.0180
123.0437
70.7760
143.2309
181.7053
Relative Abundance
m/z
F: FTMS - p ESI d Full ms2 357.1562@hcd33.33 [50.0000-385.0000]
2
195.0503
105.0179
135.0440
179.0340
70.0988
87.0073
120.8324
147.0289
225.0257
357.0827
Relative Abundance
m/z
F: FTMS - p ESI d Full ms2 152.9950@hcd33.33 [50.0000-175.0000]
3
123.0435
109.0278
69.2052
64.9973
59.4080
81.0328
97.4752
127.1564
153.0180
Relative Abundance
m/z
F: FTMS - p ESI d Full ms2 313.0569@hcd33.33 [50.0000-340.0000]
4
151.0387
69.0233
109.0279
133.6740
203.7999
230.9568
255.9368
293.1069
313.0923
Relative Abundance
m/z

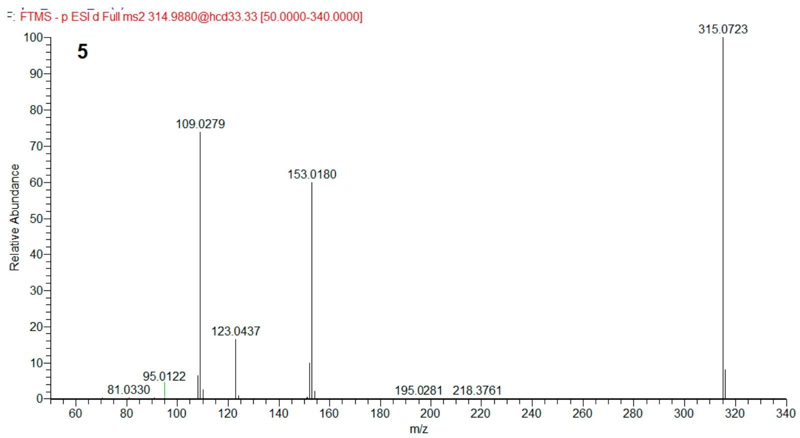
F: FTMS - p ESI d Full ms2 314.9880@hcd33.33 [50.0000-340.0000]
5
315.0723
109.0279
153.0180
123.0437
95.0122
81.0330
195.0281
218.3761
Relative Abundance
m/z

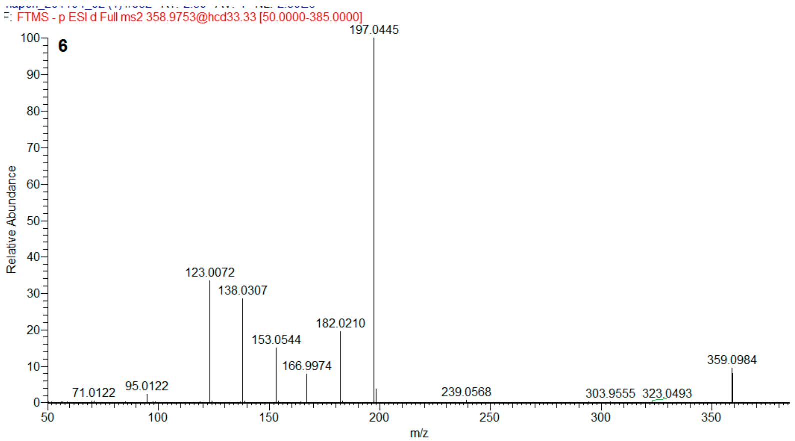
F: FTMS - p ESI d Full ms2 358.9753@hcd33.33 [50.0000-385.0000]
6
197.0445
123.0072
138.0307
182.0210
153.0544
166.9974
359.0984
71.0122
95.0122
239.0568
303.9555
323.0493
Relative Abundance
m/z

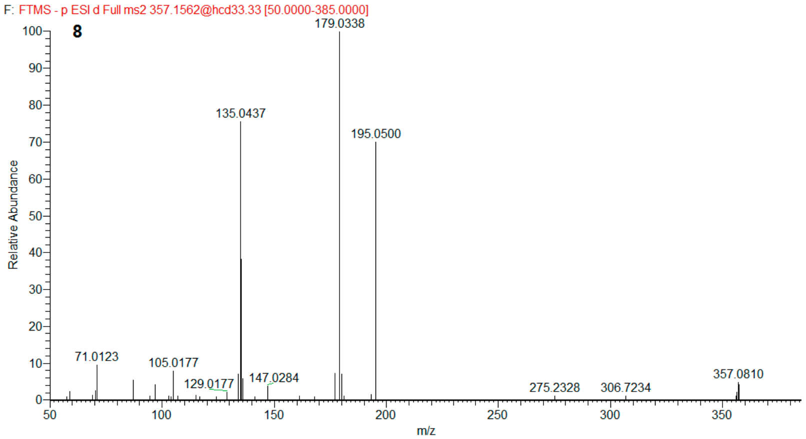
F: FTMS - p ESI d Full ms2 357.1562@hcd33.33 [50.0000-385.0000]
8
179.0338
135.0437
195.0500
71.0123
105.0177
129.0177
147.0284
275.2328
306.7234
357.0810
Relative Abundance
m/z

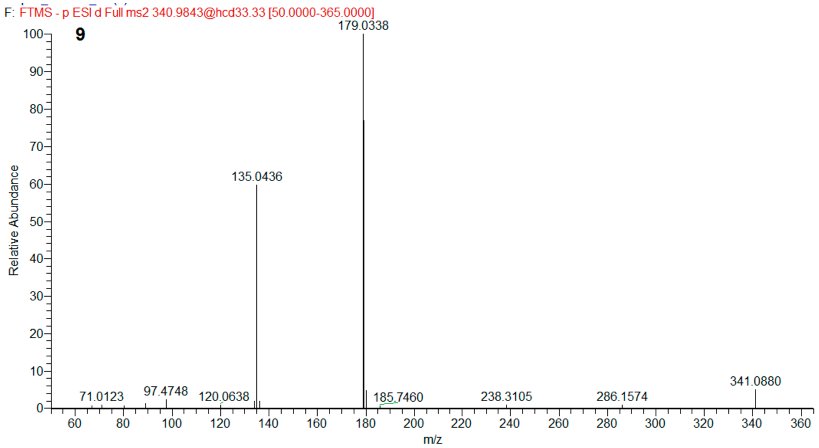
F: FTMS - p ESI d Full ms2 340.9843@hcd33.33 [50.0000-365.0000]
9
179.0338
135.0436
71.0123
97.4748
120.0638
185.7460
238.3105
286.1574
341.0880
Relative Abundance
m/z

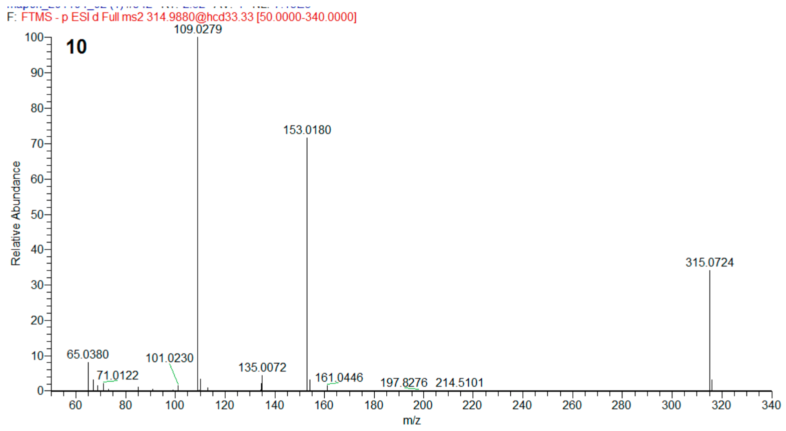
F: FTMS - p ESI d Full ms2 314.9880@hcd33.33 [50.0000-340.0000]
10
109.0279
153.0180
315.0724
65.0380
71.0122
101.0230
135.0072
161.0446
197.8276
214.5101
Relative Abundance
m/z

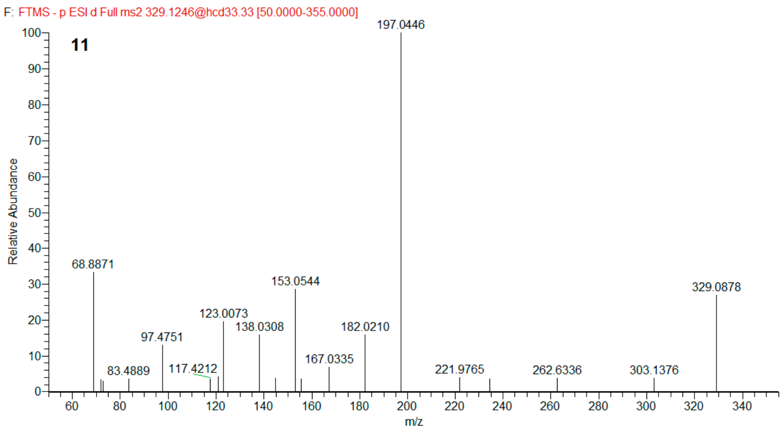
F: FTMS - p ESI d Full ms2 329.1246@hcd33.33 [50.0000-355.0000]
11
197.0446
68.8871
153.0544
329.0878
123.0073
97.4751
138.0308
182.0210
167.0335
83.4889
117.4212
221.9765
262.6336
303.1376
Relative Abundance
m/z

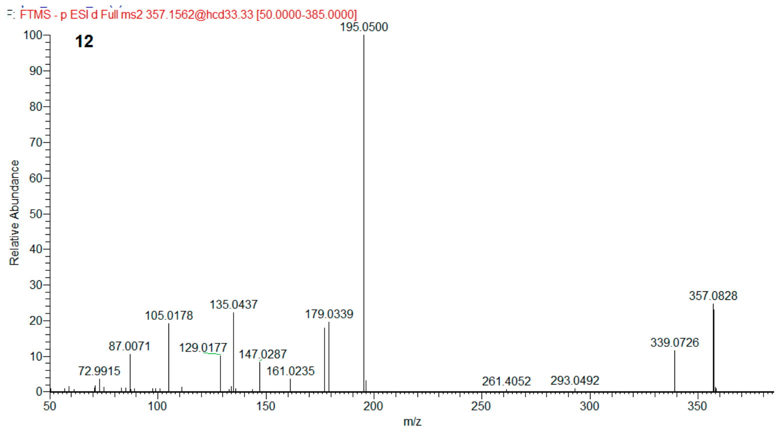
F: FTMS - p ESI d Full ms2 357.1562@hcd33.33 [50.0000-385.0000]
12
195.0500
357.0828
105.0178
135.0437
179.0339
339.0726
87.0071
129.0177
147.0287
72.9915
161.0235
261.4052
293.0492
Relative Abundance
m/z

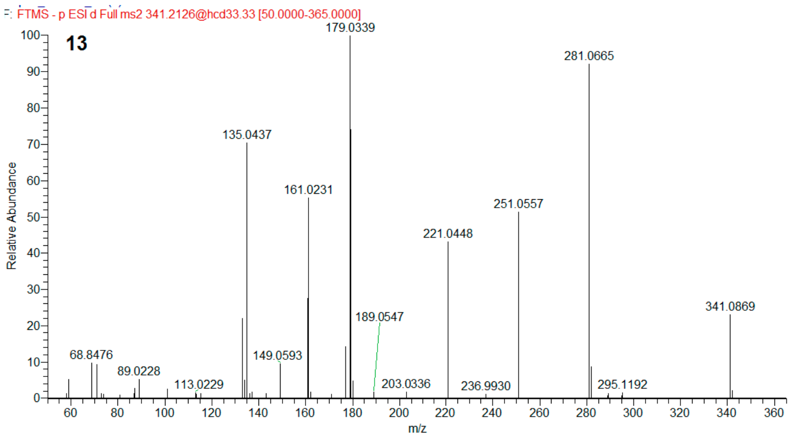
F: FTMS - p ESI d Full ms2 341.2126@hcd33.33 [50.0000-365.0000]
13
179.0339
281.0665
135.0437
161.0231
251.0557
221.0448
189.0547
341.0869
68.8476
149.0593
89.0228
113.0229
203.0336
236.9930
295.1192
Relative Abundance
m/z

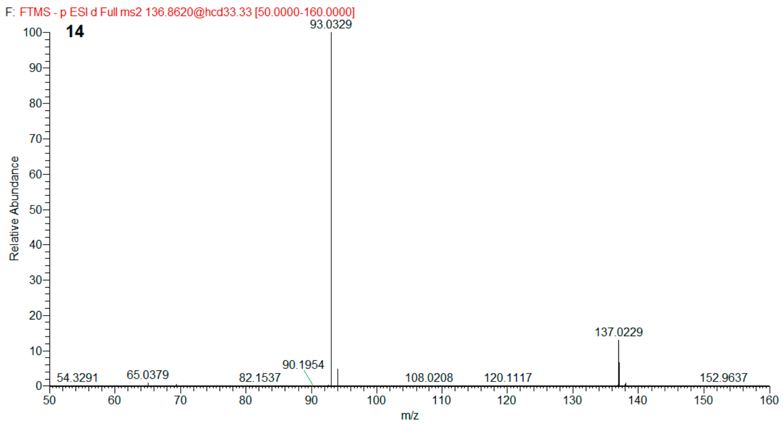
F: FTMS - p ESI d Full ms2 136.8620@hcd33.33 [50.0000-160.0000]
14
Relative Abundance
m/z
93.0329
137.0229
90.1954
54.3291
65.0379
82.1537
108.0208
120.1117
152.9637

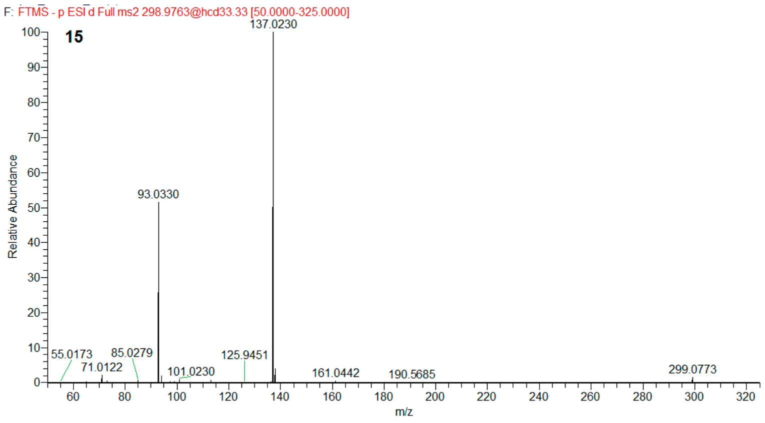
F: FTMS - p ESI d Full ms2 298.9763@hcd33.33 [50.0000-325.0000]
15
Relative Abundance
m/z
137.0230
93.0330
55.0173
85.0279
71.0122
125.9451
101.0230
161.0442
190.5685
299.0773

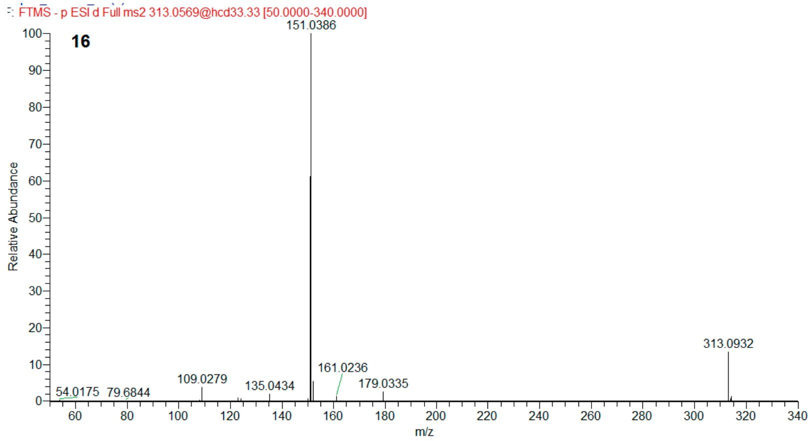
F: FTMS - p ESI d Full ms2 313.0569@hcd33.33 [50.0000-340.0000]
16
Relative Abundance
m/z
151.0386
313.0932
109.0279
161.0236
135.0434
179.0335
54.0175
79.6844

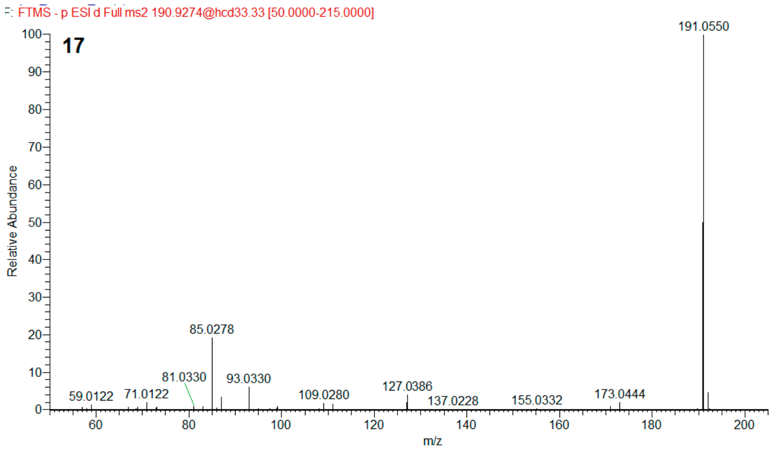
F: FTMS - p ESI d Full ms2 190.9274@hcd33.33 [50.0000-215.0000]
17
Relative Abundance
m/z
191.0550
85.0278
81.0330
93.0330
127.0386
59.0122
71.0122
109.0280
137.0228
155.0332
173.0444

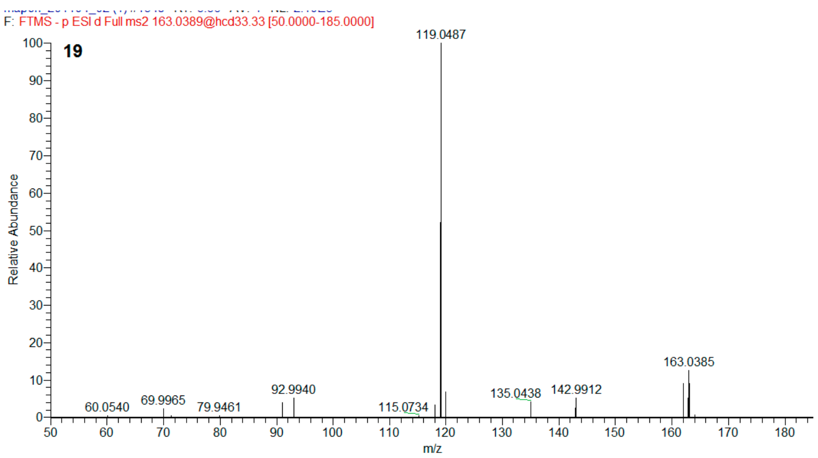
F: FTMS - p ESI d Full ms2 163.0389@hcd33.33 [50.0000-185.0000]
19
Relative Abundance
m/z
119.0487
163.0385
92.9940
142.9912
135.0438
69.9965
60.0540
79.9461
115.0734

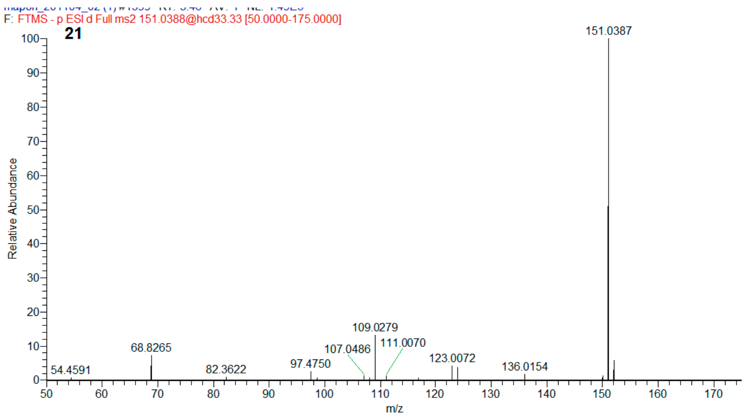
F: FTMS - p ESI d Full ms2 151.0388@hcd33.33 [50.0000-175.0000]
21
Relative Abundance
m/z
151.0387
109.0279
111.0070
107.0486
68.8265
123.0072
97.4750
136.0154
82.3622
54.4591

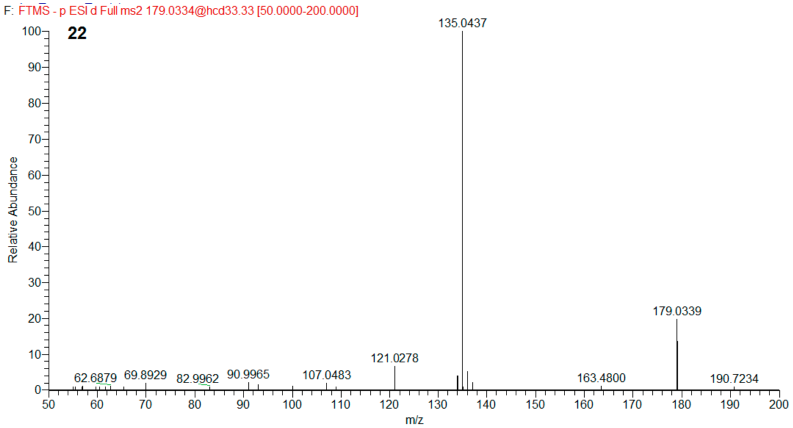
F: FTMS - p ESI d Full ms2 179.0334@hcd33.33 [50.0000-200.0000]
22
Relative Abundance
m/z
135.0437
179.0339
121.0278
62.6879
69.8929
82.9962
90.9965
107.0483
163.4800
190.7234

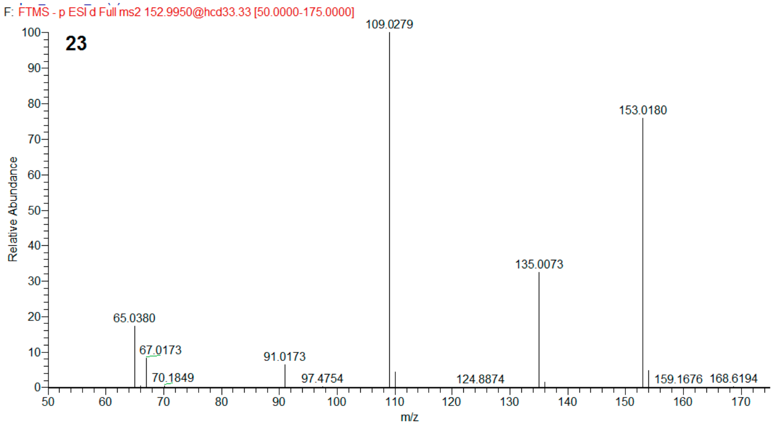
F: FTMS - p ESI d Full ms2 152.9950@hcd33.33 [50.0000-175.0000]
23
Relative Abundance
m/z
109.0279
153.0180
135.0073
65.0380
67.0173
91.0173
70.1849
97.4754
124.8874
159.1676
168.6194

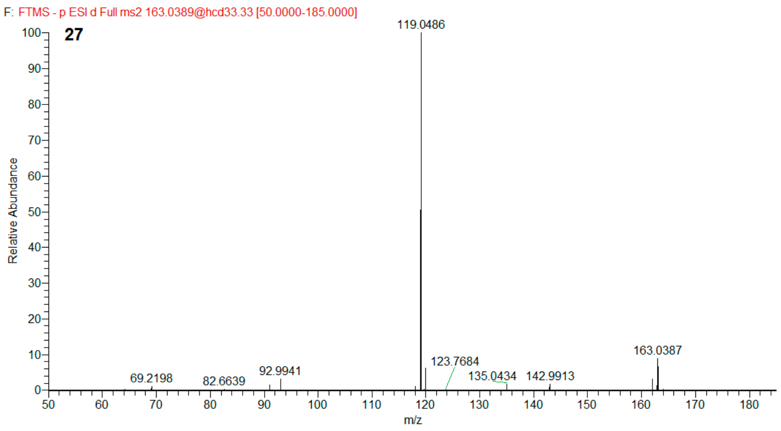
F: FTMS - p ESI d Full ms2 163.0389@hcd33.33 [50.0000-185.0000]
27
119.0486
123.7684
135.0434
142.9913
163.0387
69.2198
82.6639
92.9941
Relative Abundance
m/z

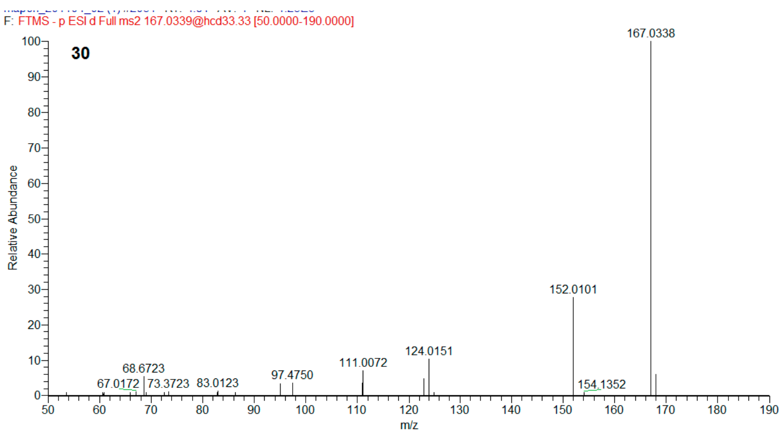
F: FTMS - p ESI d Full ms2 167.0339@hcd33.33 [50.0000-190.0000]
30
167.0338
152.0101
124.0151
111.0072
97.4750
68.6723
67.0172
73.3723
83.0123
154.1352
Relative Abundance
m/z

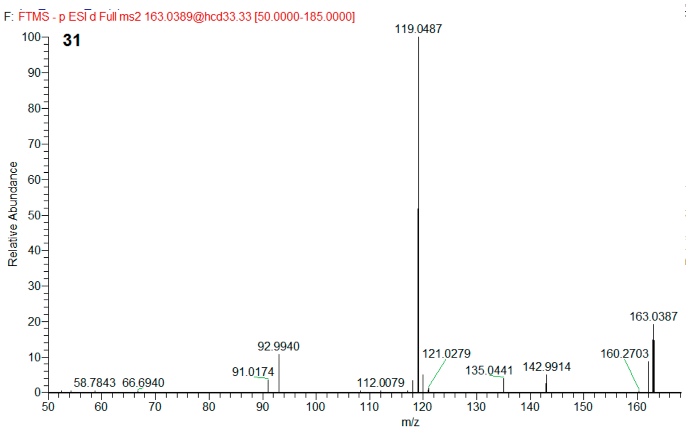
F: FTMS - p ESI d Full ms2 163.0389@hcd33.33 [50.0000-185.0000]
31
119.0487
163.0387
92.9940
121.0279
160.2703
91.0174
135.0441
142.9914
58.7843
66.6940
112.0079
Relative Abundance
m/z

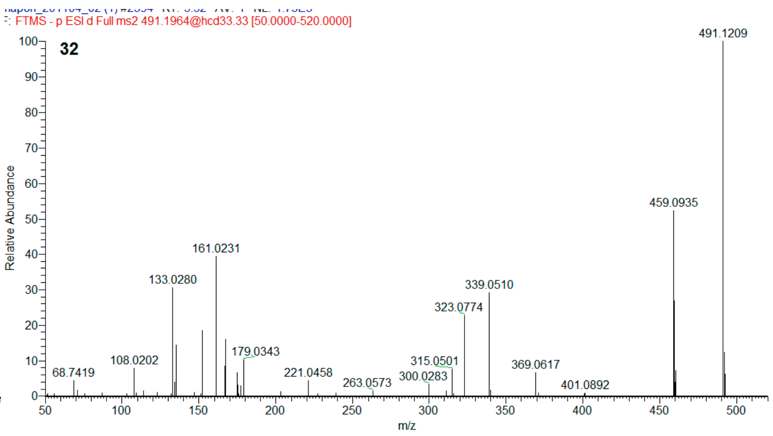
FTMS - p ESI d Full ms2 491.1964@hcd33.33 [50.0000-520.0000]
32
491.1209
459.0935
161.0231
133.0280
339.0510
323.0774
179.0343
108.0202
68.7419
221.0458
263.0573
300.0283
315.0501
369.0617
401.0892
Relative Abundance
m/z

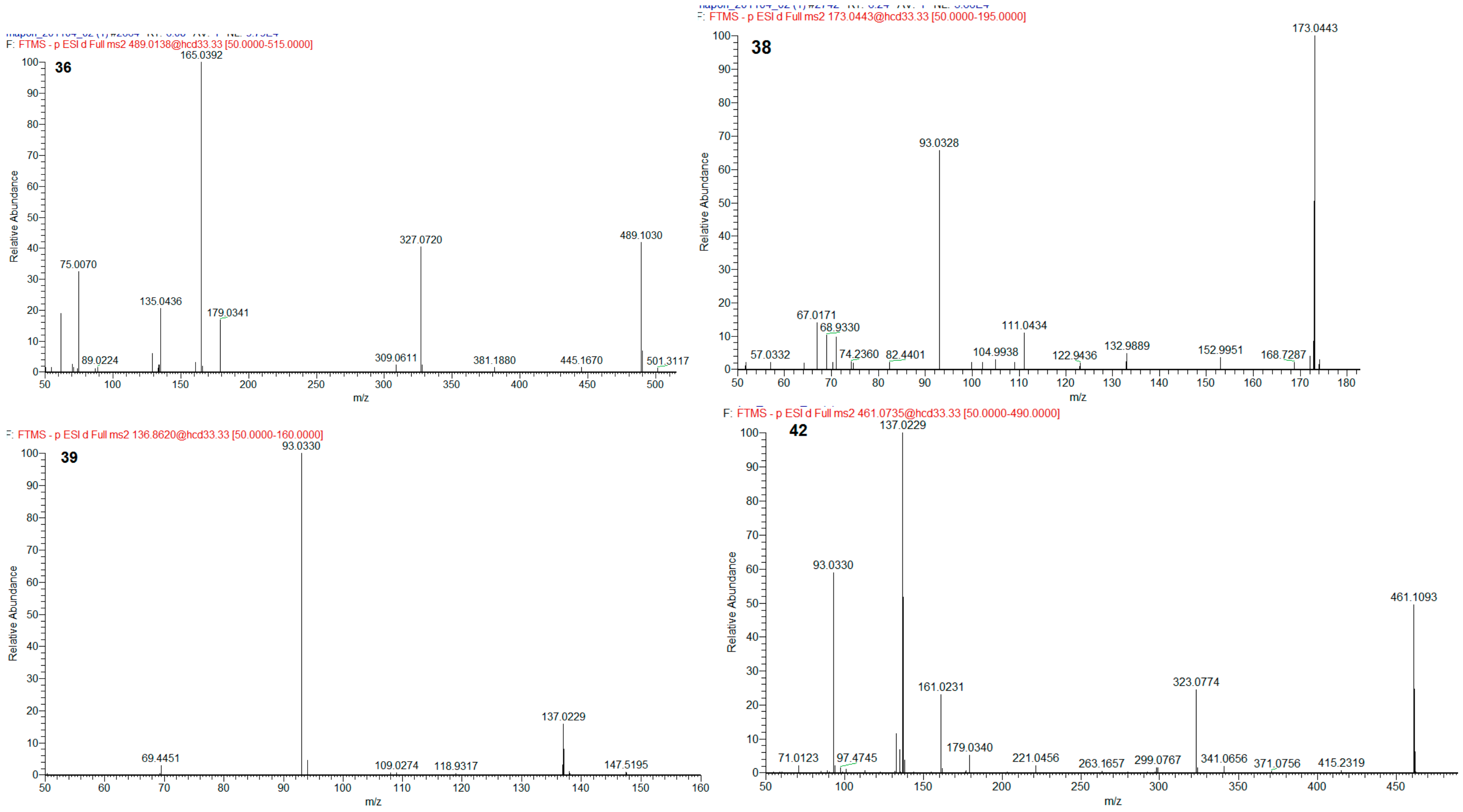

Figure S5. MS/MS spectra of hydroxybenzoic, hydroxycinnamic acids and their glycosides, and sugar esters (for the compound numbers see Table 2).

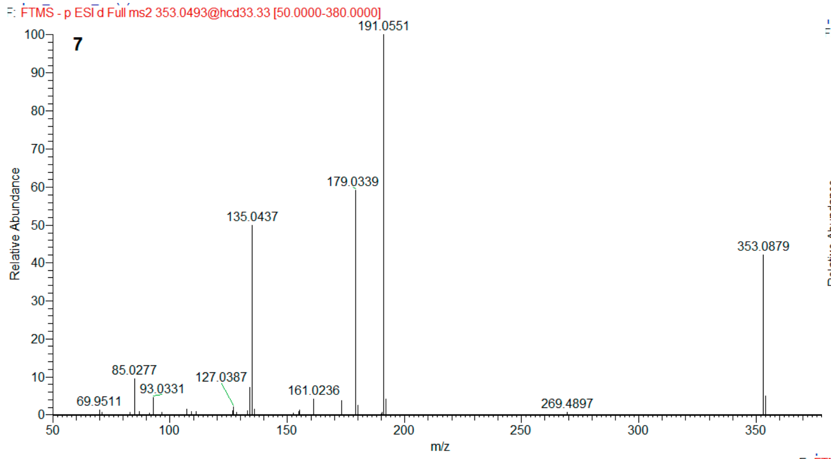
F: FTMS - p ESI d Full ms2 353.0493@hcd33.33 [50.0000-380.0000]
7
191.0551
179.0339
135.0437
353.0879
85.0277
93.0331
127.0387
161.0236
69.9511
269.4897
Relative Abundance
m/z

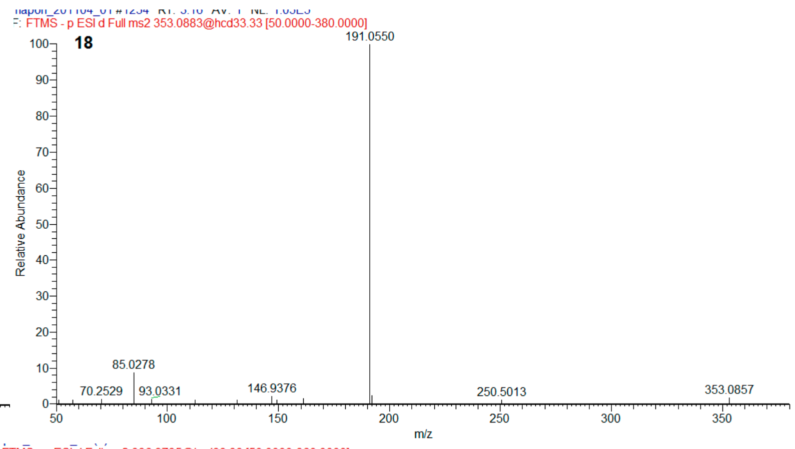
F: FTMS - p ESI d Full ms2 353.0883@hcd33.33 [50.0000-380.0000]
18
191.0550
85.0278
70.2529
93.0331
146.9376
250.5013
353.0857
Relative Abundance
m/z

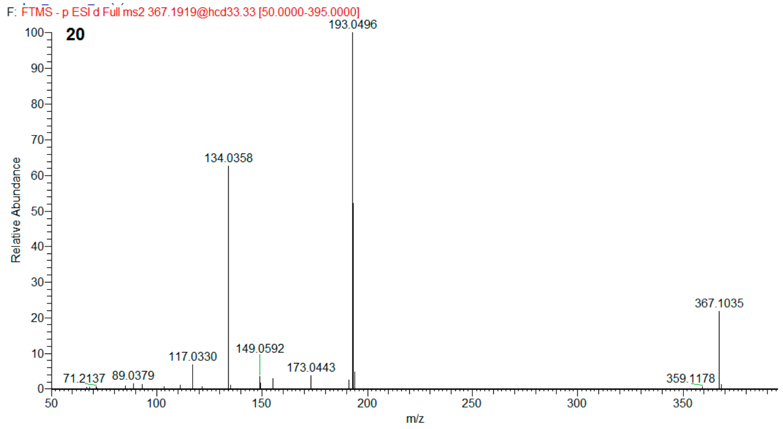
F: FTMS - p ESI d Full ms2 367.1919@hcd33.33 [50.0000-395.0000]
20
193.0496
134.0358
367.1035
117.0330
149.0592
173.0443
71.2137
89.0379
359.1178
Relative Abundance
m/z

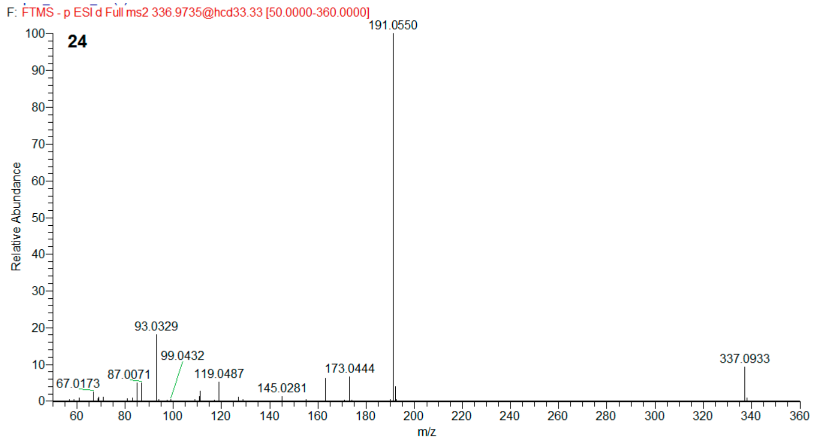
F: FTMS - p ESI d Full ms2 336.9735@hcd33.33 [50.0000-360.0000]
24
191.0550
93.0329
99.0432
87.0071
67.0173
119.0487
145.0281
173.0444
337.0933
Relative Abundance
m/z

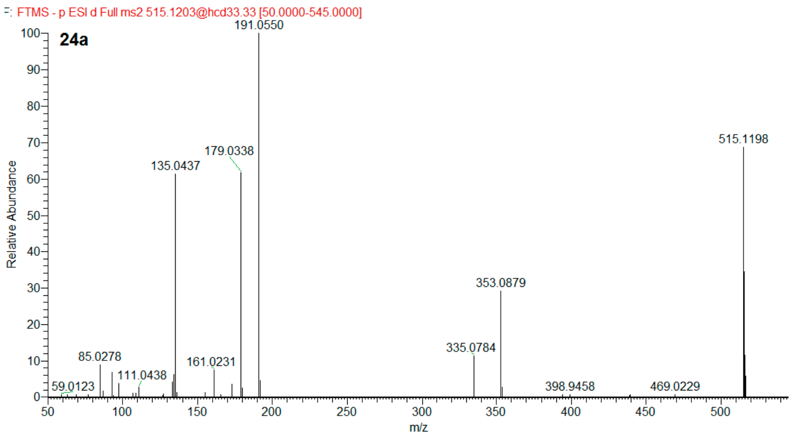
F: FTMS - p ESI d Full ms2 515.1203@hcd33.33 [50.0000-545.0000]
24a
191.0550
515.1198
179.0338
135.0437
353.0879
335.0784
85.0278
161.0231
111.0438
59.0123
398.9458
469.0229
Relative Abundance
m/z

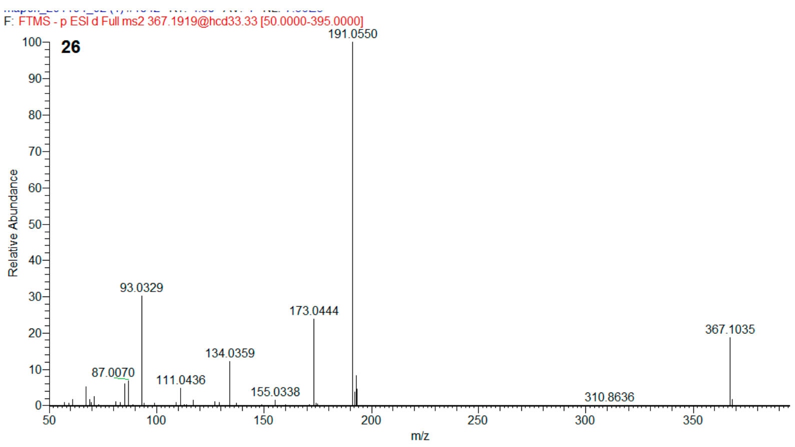
F: FTMS - p ESI d Full ms2 367.1919@hcd33.33 [50.0000-395.0000]
26
191.0550
93.0329
173.0444
367.1035
134.0359
87.0070
111.0436
155.0338
310.8636
Relative Abundance
m/z

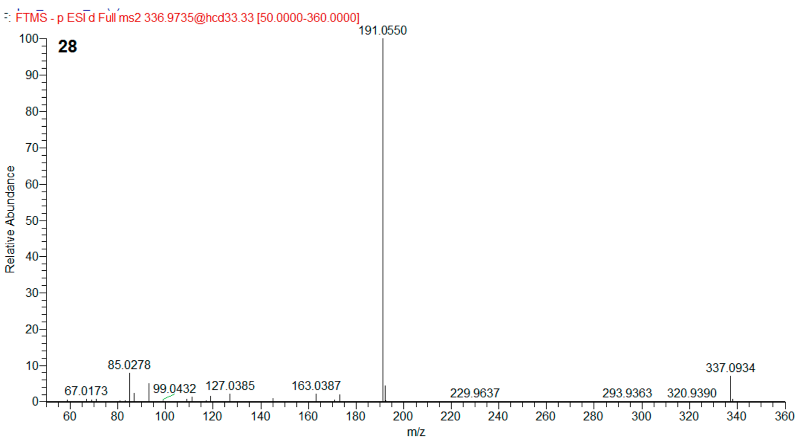
F: FTMS - p ESI d Full ms2 336.9735@hcd33.33 [50.0000-360.0000]
28
191.0550
85.0278
337.0934
67.0173
99.0432
127.0385
163.0387
229.9637
293.9363
320.9390
Relative Abundance
m/z

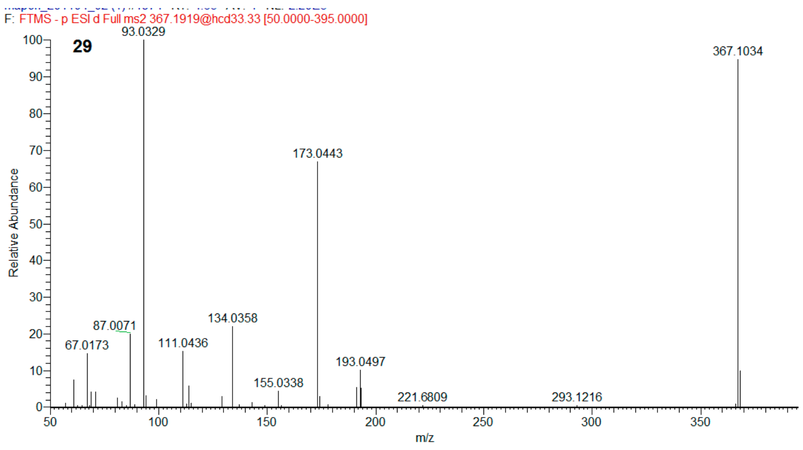
F: FTMS - p ESI d Full ms2 367.1919@hcd33.33 [50.0000-395.0000]
29
93.0329
367.1034
173.0443
87.0071
134.0358
67.0173
111.0436
193.0497
155.0338
221.6809
293.1216
Relative Abundance
m/z

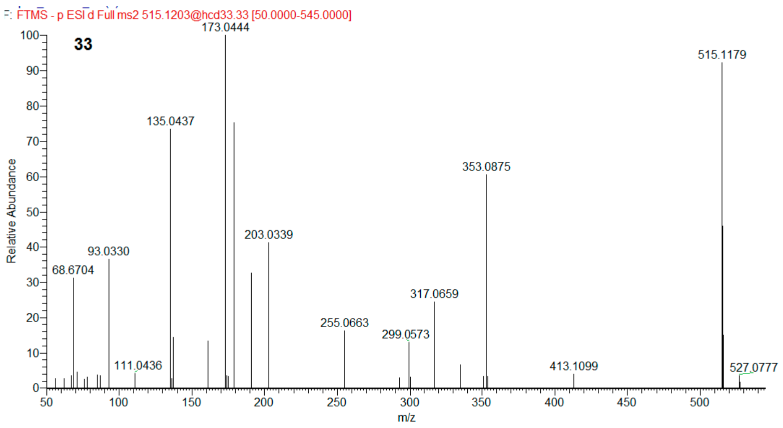
F: FTMS - p ESI d Full ms2 515.1203@hcd33.33 [50.0000-545.0000]
33
173.0444
515.1179
135.0437
353.0875
203.0339
93.0330
68.6704
317.0659
255.0663
299.0573
111.0436
413.1099
527.0777
Relative Abundance
m/z

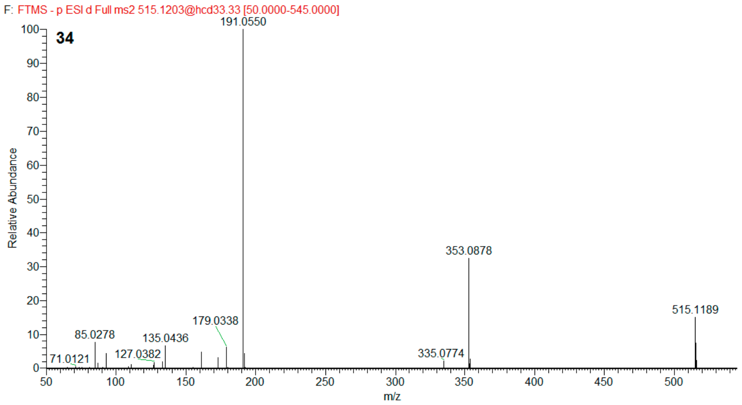
F: FTMS - p ESI d Full ms2 515.1203@hcd33.33 [50.0000-545.0000]
34
191.0550
353.0878
515.1189
179.0338
85.0278
135.0436
127.0382
71.0121
335.0774
Relative Abundance
m/z

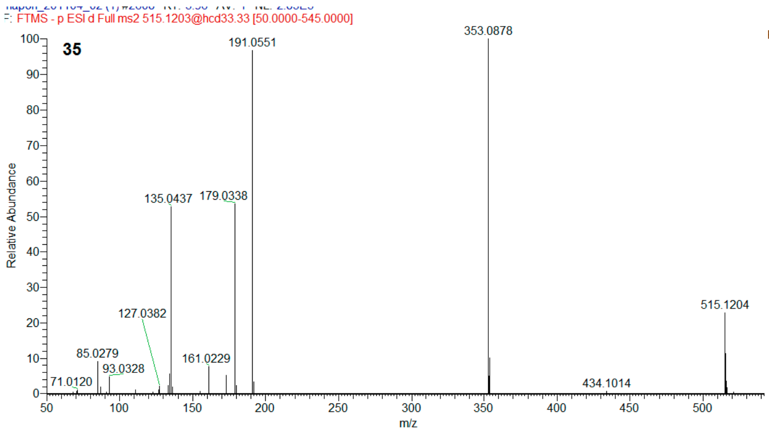
F: FTMS - p ESI d Full ms2 515.1203@hcd33.33 [50.0000-545.0000]
35
353.0878
191.0551
135.0437
179.0338
127.0382
515.1204
85.0279
93.0328
161.0229
71.0120
434.1014
Relative Abundance
m/z

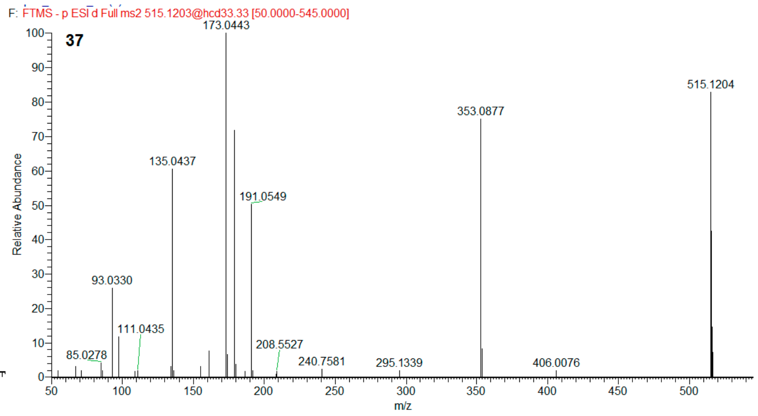
F: FTMS - p ESI d Full ms2 515.1203@hcd33.33 [50.0000-545.0000]
37
173.0443
515.1204
353.0877
135.0437
191.0549
93.0330
111.0435
85.0278
208.5527
240.7581
295.1339
406.0076
Relative Abundance
m/z

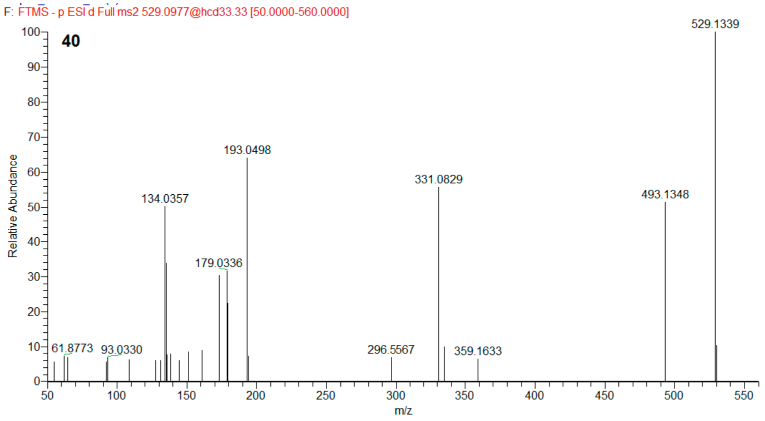
F: FTMS - p ESI d Full ms2 529.0977@hcd33.33 [50.0000-560.0000]
40
529.1339
193.0498
331.0829
493.1348
134.0357
179.0336
61.8773
93.0330
296.5567
359.1633
Relative Abundance
m/z

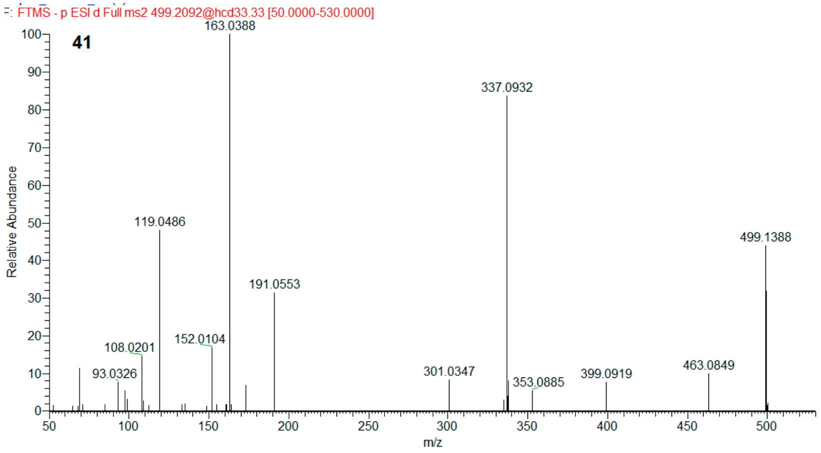
F: FTMS - p ESI d Full ms2 499.2092@hcd33.33 [50.0000-530.0000]
41
163.0388
337.0932
119.0486
499.1388
191.0553
152.0104
108.0201
93.0326
301.0347
353.0885
399.0919
463.0849
Relative Abundance
m/z

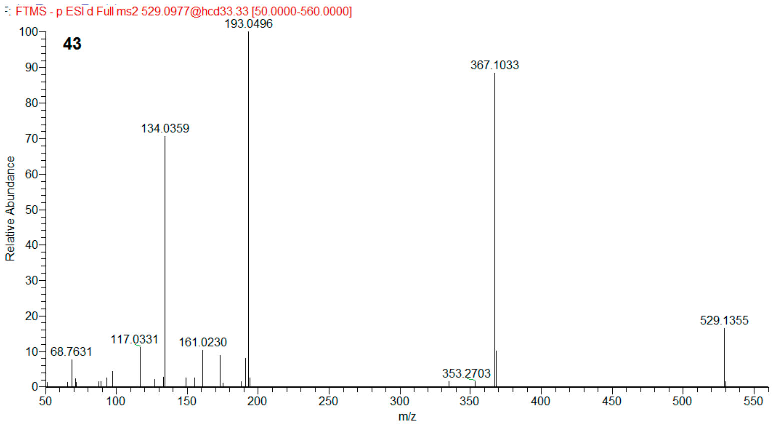
F: FTMS - p ESI d Full ms2 529.0977@hcd33.33 [50.0000-560.0000]
43
193.0496
367.1033
134.0359
529.1355
68.7631
117.0331
161.0230
353.2703
Relative Abundance
m/z

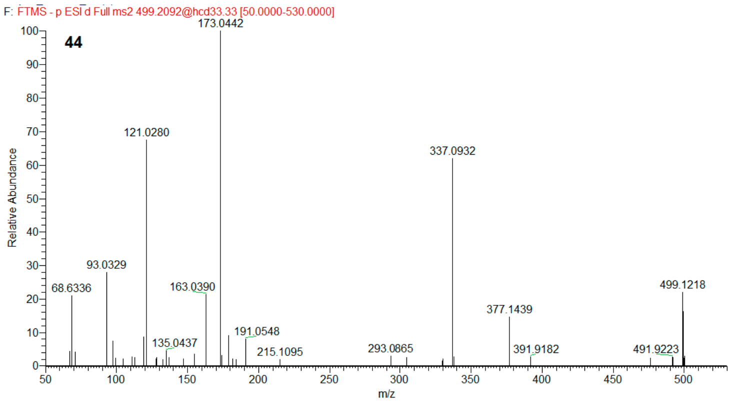
F: FTMS - p ESI d Full ms2 499.2092@hcd33.33 [50.0000-530.0000]
44
173.0442
121.0280
337.0932
93.0329
68.6336
163.0390
499.1218
377.1439
135.0437
191.0548
215.1095
293.0865
391.9182
491.9223
Relative Abundance
m/z

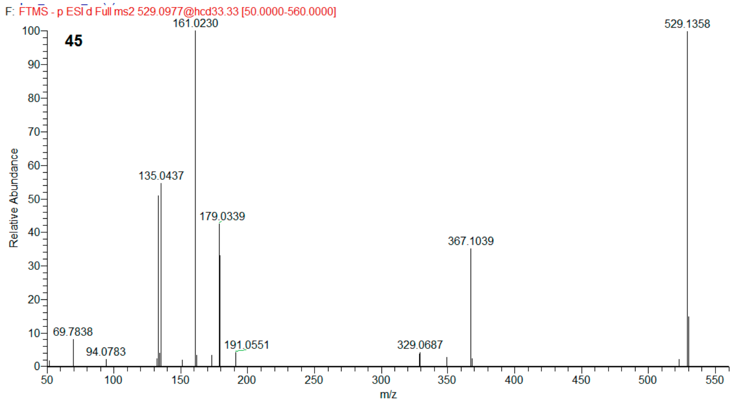

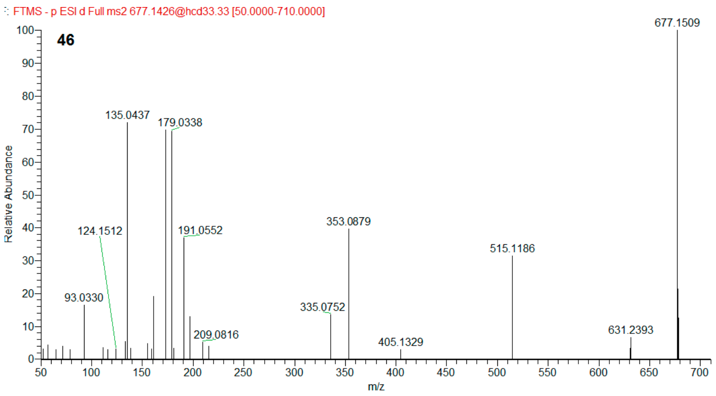

Figure S6. MS/MS spectra of acylquinic acids (for the compound numbers see Table 2).

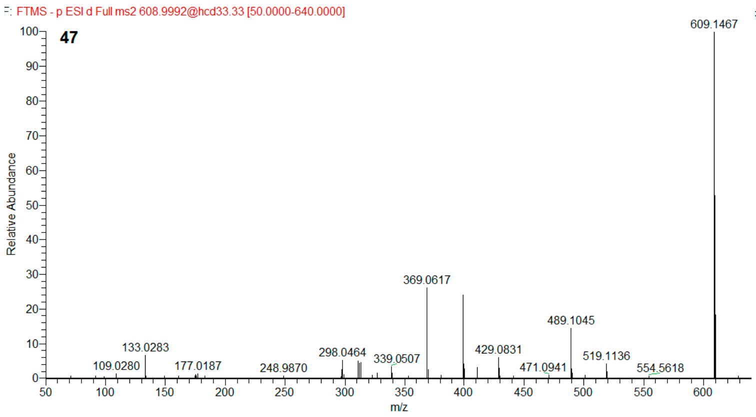
F: FTMS - p ESI d Full ms2 608.9992@hcd33.33 [50.0000-640.0000]
47
609.1467
369.0617
489.1045
133.0283
298.0464
429.0831
519.1136
109.0280
177.0187
248.9870
339.0507
471.0941
554.5618
Relative Abundance
m/z

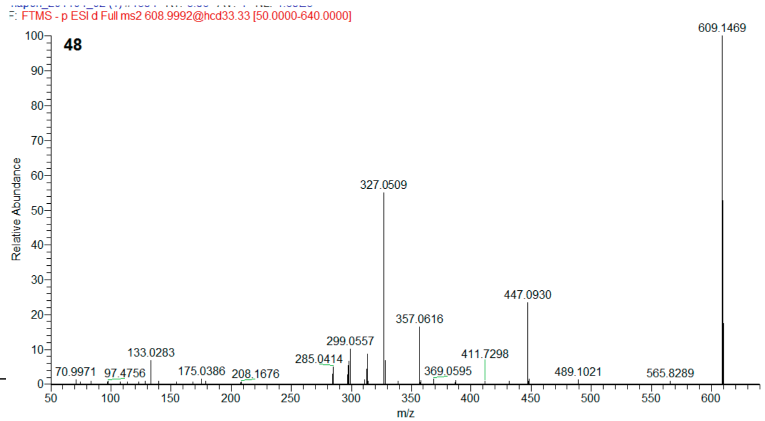
F: FTMS - p ESI d Full ms2 608.9992@hcd33.33 [50.0000-640.0000]
48
609.1469
327.0509
447.0930
357.0616
299.0557
133.0283
285.0414
411.7298
70.9971
97.4756
175.0386
208.1676
369.0595
489.1021
565.8289
Relative Abundance
m/z

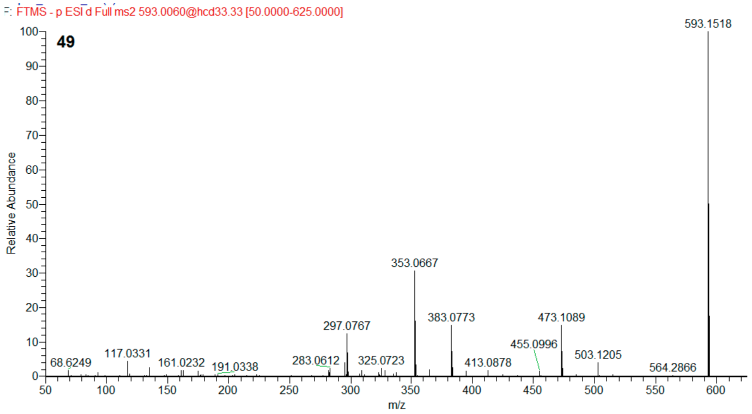
F: FTMS - p ESI d Full ms2 593.0060@hcd33.33 [50.0000-625.0000]
49
593.1518
353.0667
383.0773
473.1089
297.0767
455.0996
117.0331
503.1205
68.6249
161.0232
191.0338
283.0612
325.0723
413.0878
564.2866
Relative Abundance
m/z

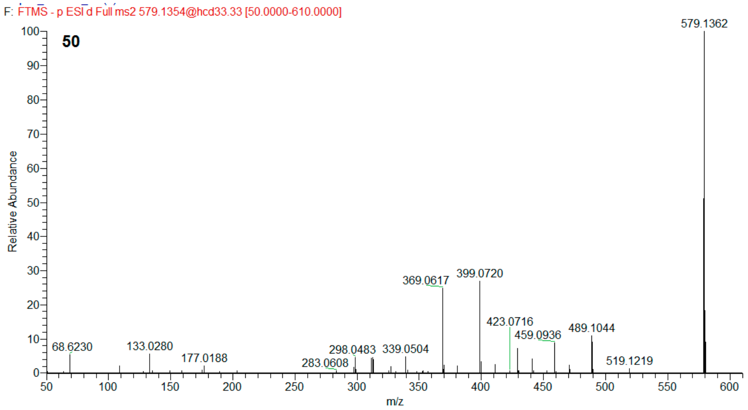
F: FTMS - p ESI d Full ms2 579.1354@hcd33.33 [50.0000-610.0000]
50
579.1362
399.0720
369.0617
423.0716
489.1044
459.0936
68.6230
133.0280
298.0483
339.0504
177.0188
283.0608
519.1219
Relative Abundance
m/z

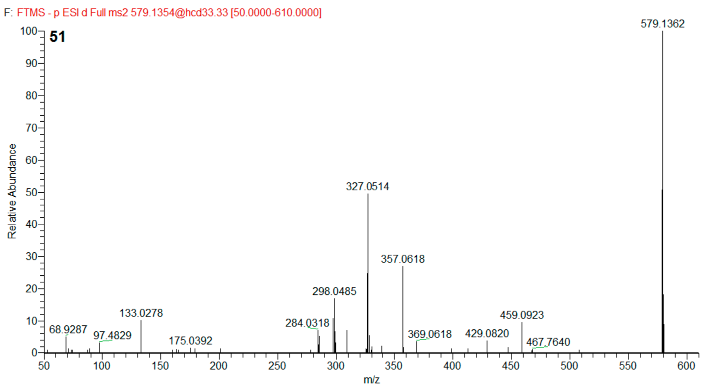
F: FTMS - p ESI d Full ms2 579.1354@hcd33.33 [50.0000-610.0000]
51
Relative Abundance
m/z
579.1362
327.0514
357.0618
298.0485
284.0318
133.0278
68.9287
97.4829
175.0392
369.0618
429.0820
459.0923
467.7640

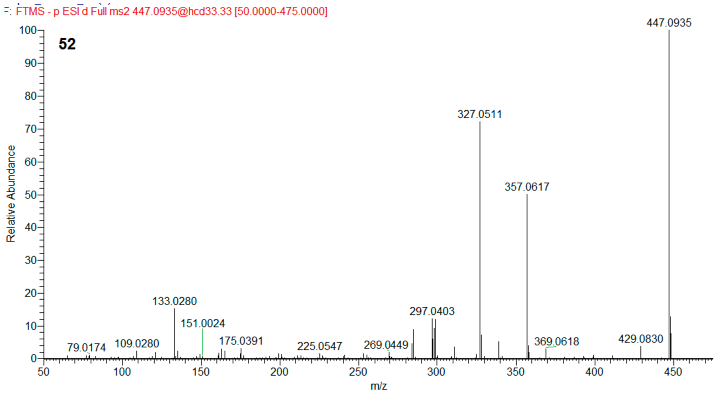
F: FTMS - p ESI d Full ms2 447.0935@hcd33.33 [50.0000-475.0000]
52
Relative Abundance
m/z
447.0935
327.0511
357.0617
133.0280
151.0024
297.0403
79.0174
109.0280
175.0391
225.0547
269.0449
369.0618
429.0830

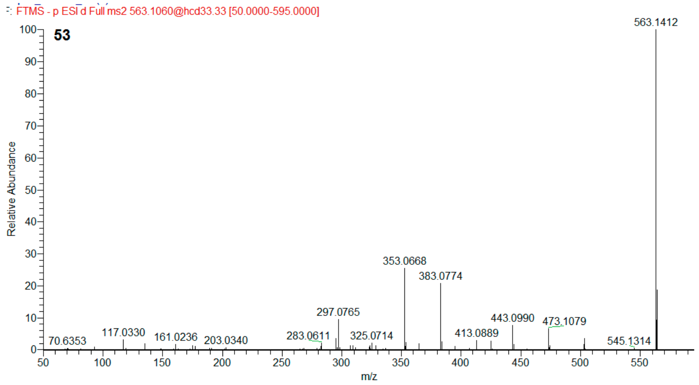
F: FTMS - p ESI d Full ms2 563.1060@hcd33.33 [50.0000-595.0000]
53
Relative Abundance
m/z
563.1412
353.0668
383.0774
297.0765
443.0990
473.1079
70.6353
117.0330
161.0236
203.0340
283.0611
325.0714
413.0889
545.1314

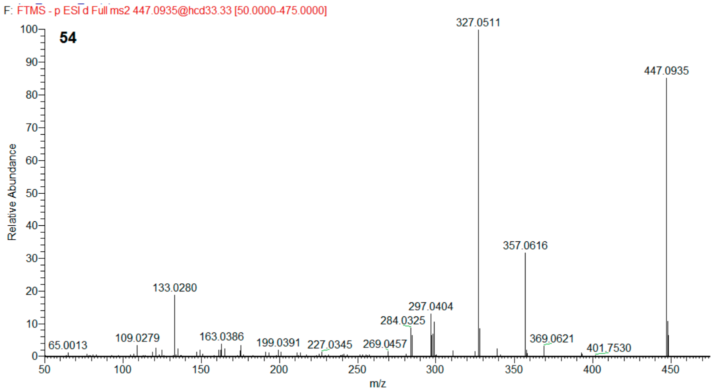
F: FTMS - p ESI d Full ms2 447.0935@hcd33.33 [50.0000-475.0000]
54
Relative Abundance
m/z
327.0511
447.0935
357.0616
133.0280
297.0404
284.0325
65.0013
109.0279
163.0386
199.0391
227.0345
269.0457
369.0621
401.7530

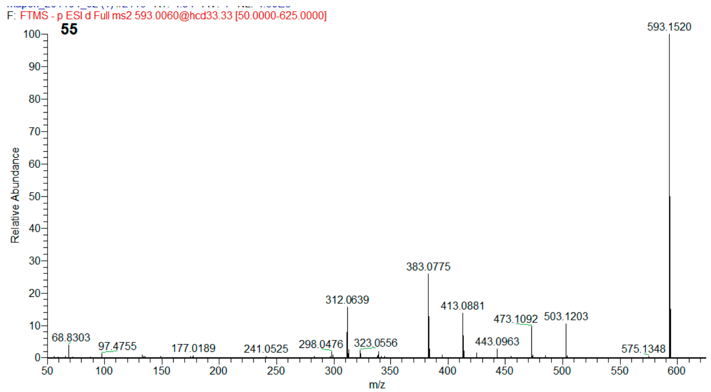
F: FTMS - p ESI d Full ms2 593.0060@hcd33.33 [50.0000-625.0000]
55
593.1520
383.0775
312.0639
413.0881
473.1092
503.1203
443.0963
68.8303
97.4755
177.0189
241.0525
298.0476
323.0556
575.1348
Relative Abundance
m/z

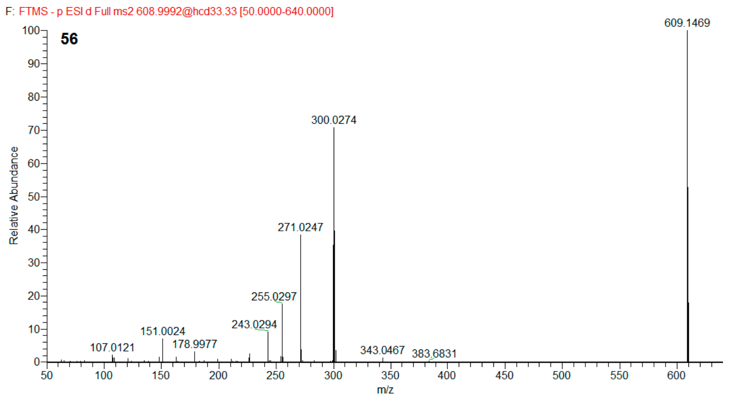
F: FTMS - p ESI d Full ms2 608.9992@hcd33.33 [50.0000-640.0000]
56
609.1469
300.0274
271.0247
255.0297
243.0294
151.0024
178.9977
107.0121
343.0467
383.6831
Relative Abundance
m/z

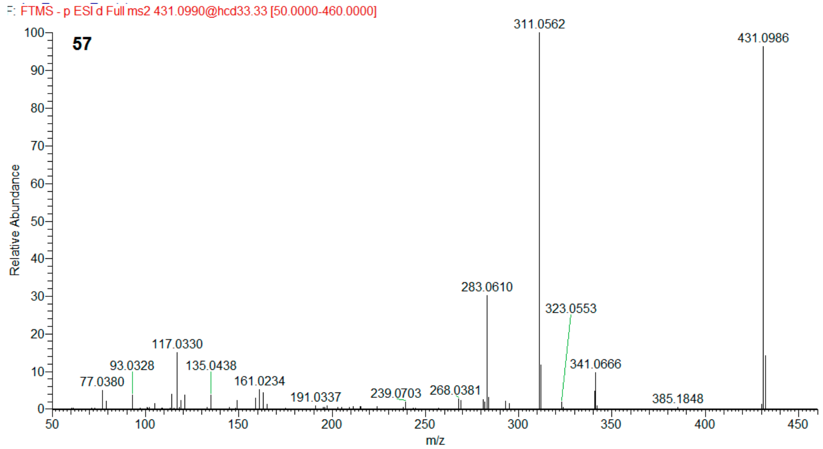
F: FTMS - p ESI d Full ms2 431.0990@hcd33.33 [50.0000-460.0000]
57
311.0562
431.0986
283.0610
323.0553
341.0666
117.0330
93.0328
135.0438
77.0380
161.0234
191.0337
239.0703
268.0381
385.1848
Relative Abundance
m/z

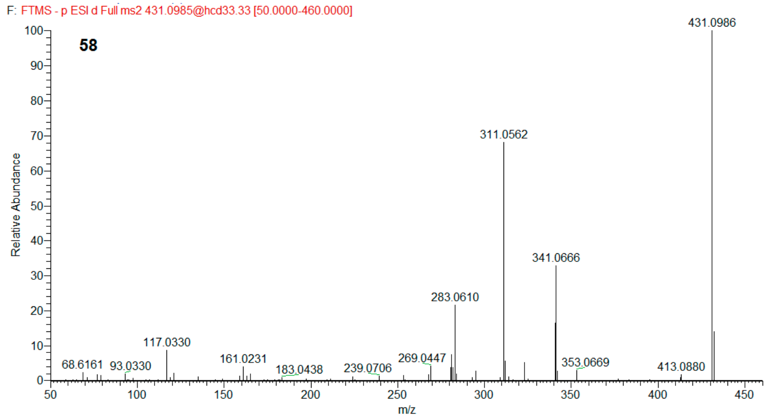
F: FTMS - p ESI d Full ms2 431.0985@hcd33.33 [50.0000-460.0000]
58
431.0986
311.0562
341.0666
283.0610
117.0330
161.0231
269.0447
353.0669
68.6161
93.0330
183.0438
239.0706
413.0880
Relative Abundance
m/z

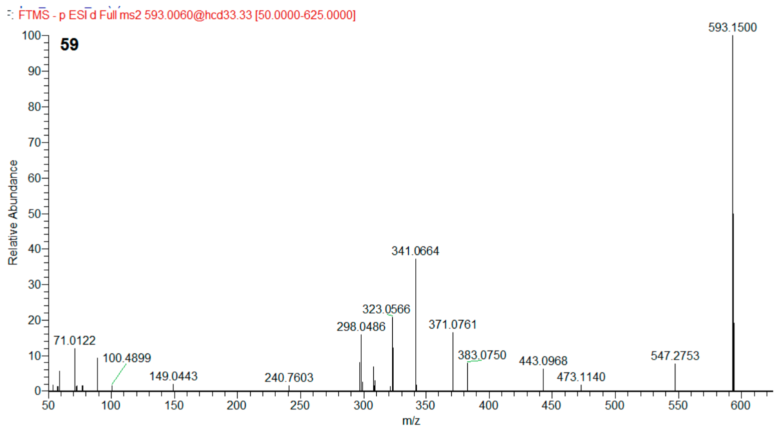
FTMS - p ESI d Full ms2 593.0060@hcd33.33 [50.0000-625.0000]
59
593.1500
341.0664
323.0566
298.0486
371.0761
383.0750
443.0968
473.1140
547.2753
71.0122
100.4899
149.0443
240.7603
Relative Abundance
m/z

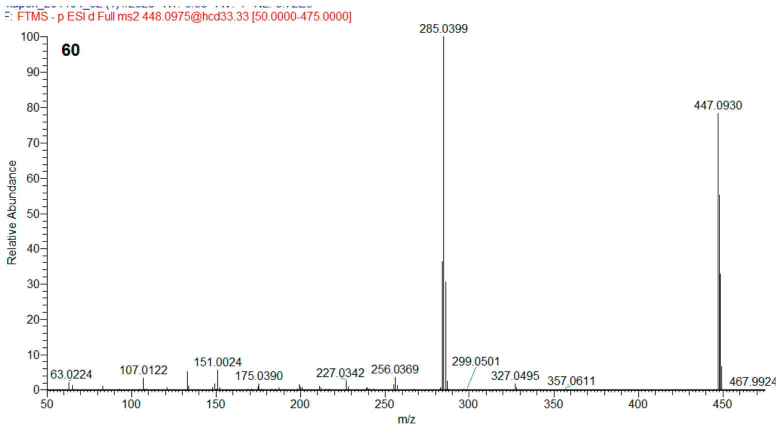
FTMS - p ESI d Full ms2 448.0975@hcd33.33 [50.0000-475.0000]
60
285.0399
447.0930
63.0224
107.0122
151.0024
175.0390
227.0342
256.0369
299.0501
327.0495
357.0611
467.9924
Relative Abundance
m/z

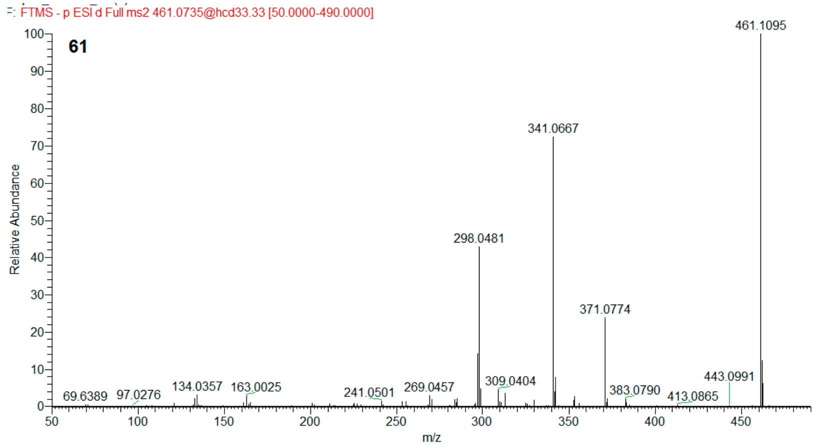
FTMS - p ESI d Full ms2 461.0735@hcd33.33 [50.0000-490.0000]
61
461.1095
341.0667
298.0481
371.0774
69.6389
97.0276
134.0357
163.0025
241.0501
269.0457
309.0404
383.0790
413.0865
443.0991
Relative Abundance
m/z

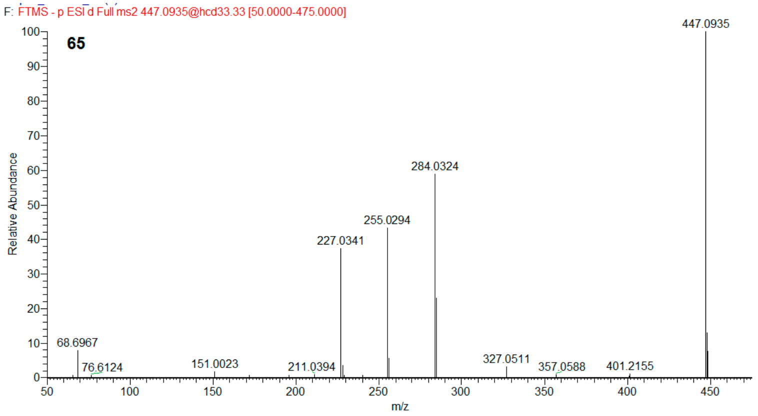
F: FTMS - p ESI d Full ms2 447.0935@hcd33.33 [50.0000-475.0000]
65
447.0935
284.0324
255.0294
227.0341
68.6967
76.6124
151.0023
211.0394
327.0511
357.0588
401.2155
Relative Abundance
m/z

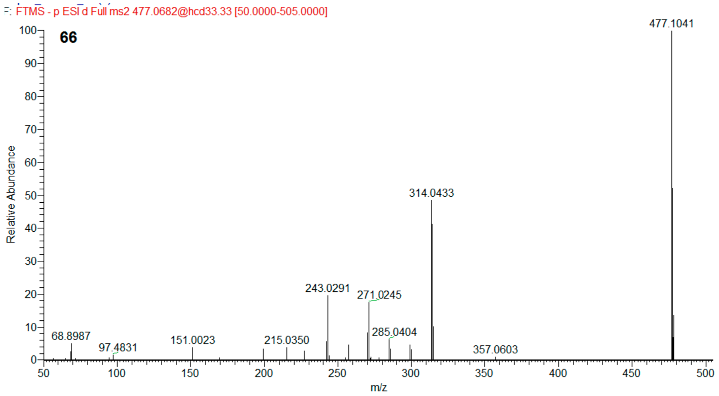
FTMS - p ESI d Full ms2 477.0682@hcd33.33 [50.0000-505.0000]
66
477.1041
314.0433
243.0291
271.0245
285.0404
68.8987
97.4831
151.0023
215.0350
357.0603
Relative Abundance
m/z

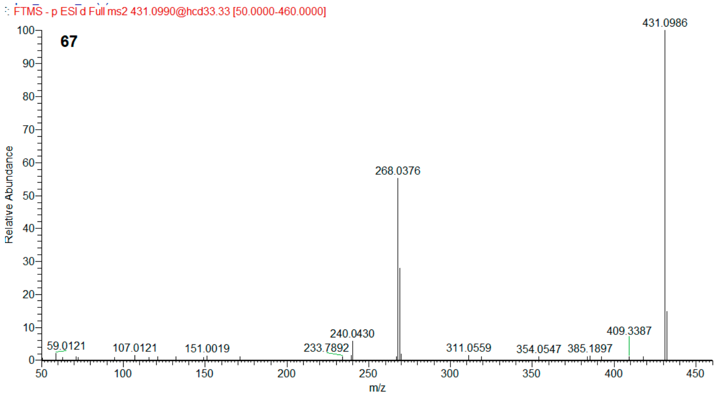
FTMS - p ESI d Full ms2 431.0990@hcd33.33 [50.0000-460.0000]
67
431.0986
268.0376
240.0430
409.3387
59.0121
107.0121
151.0019
233.7892
311.0559
354.0547
385.1897
Relative Abundance
m/z

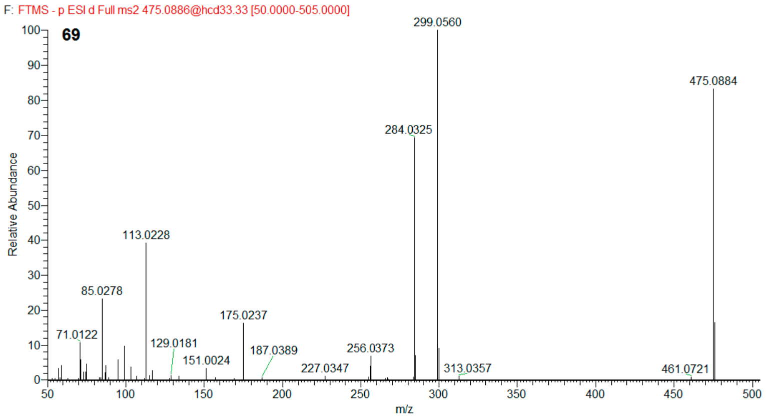
F: FTMS - p ESI d Full ms2 475.0886@hcd33.33 [50.0000-505.0000]
69
299.0560
475.0884
284.0325
113.0228
85.0278
175.0237
71.0122
129.0181
151.0024
187.0389
256.0373
227.0347
313.0357
461.0721
Relative Abundance
m/z

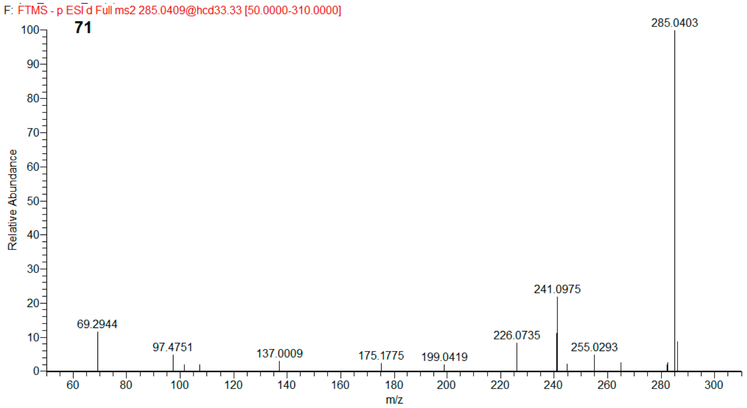
F: FTMS - p ESI d Full ms2 285.0409@hcd33.33 [50.0000-310.0000]
71
285.0403
241.0975
69.2944
97.4751
137.0009
175.1775
199.0419
226.0735
255.0293
Relative Abundance
m/z

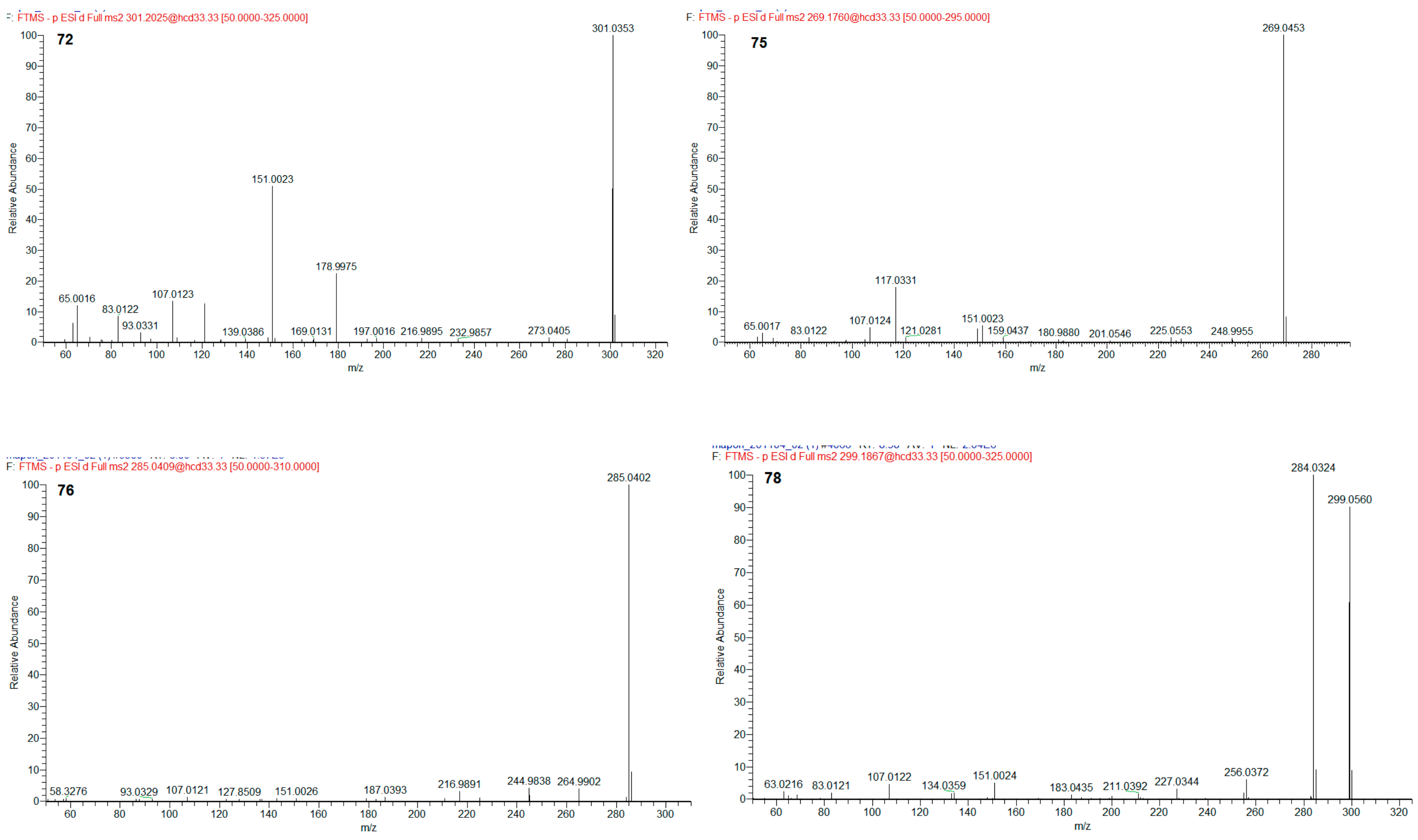

Figure S7. MS/MS spectra of C-, C,O- and O-flavonoid glycosides(for the compound numbers see Table 2).

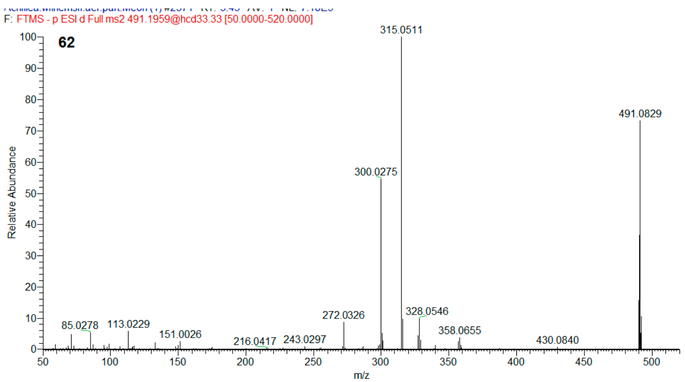
F: FTMS - p ESI d Full ms2 491.1959@hcd33.33 [50.0000-520.0000]
62
315.0511
491.0829
300.0275
85.0278
113.0229
151.0026
216.0417
243.0297
272.0326
328.0546
358.0655
430.0840
Relative Abundance
m/z

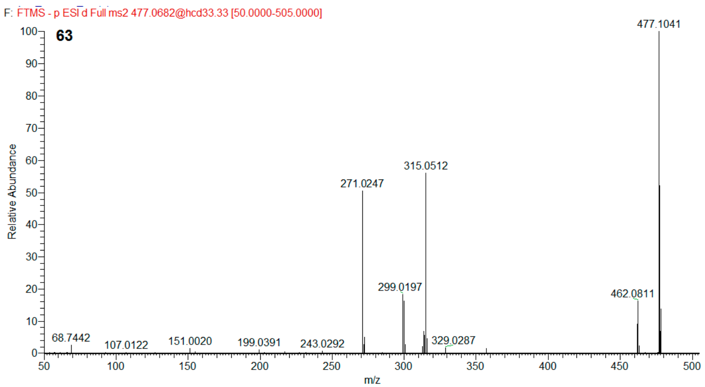
F: FTMS - p ESI d Full ms2 477.0682@hcd33.33 [50.0000-505.0000]
63
477.1041
315.0512
271.0247
299.0197
462.0811
68.7442
107.0122
151.0020
199.0391
243.0292
329.0287
Relative Abundance
m/z

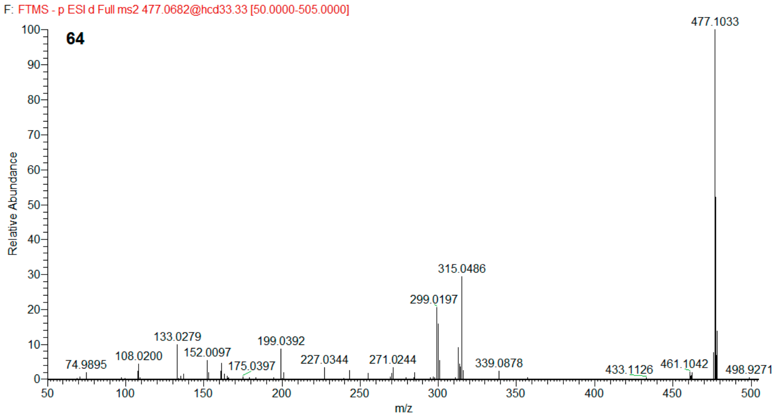
F: FTMS - p ESI d Full ms2 477.0682@hcd33.33 [50.0000-505.0000]
64
477.1033
315.0486
299.0197
133.0279
152.0097
199.0392
74.9895
108.0200
175.0397
227.0344
271.0244
339.0878
433.1126
461.1042
498.9271
Relative Abundance
m/z

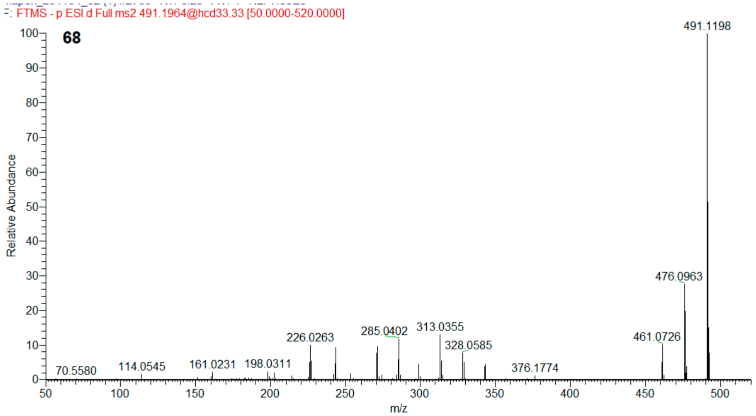
F: FTMS - p ESI d Full ms2 491.1964@hcd33.33 [50.0000-520.0000]
68
491.1198
476.0963
461.0726
226.0263
285.0402
313.0355
328.0585
70.5580
114.0545
161.0231
198.0311
376.1774
Relative Abundance
m/z

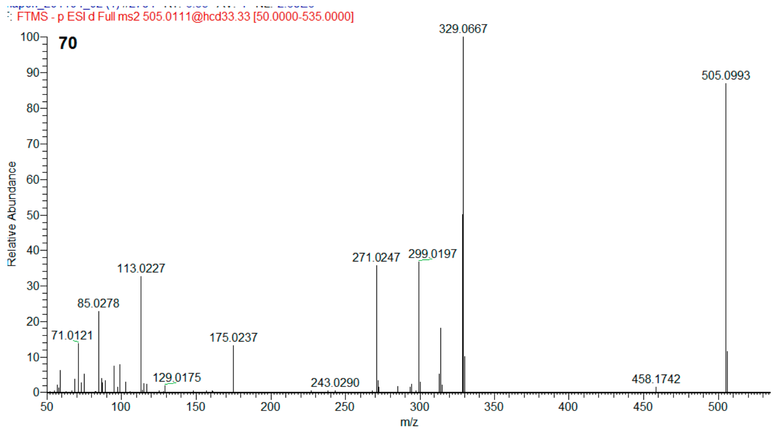
FTMS - p ESI d Full ms2 505.0111@hcd33.33 [50.0000-535.0000]
70
Relative Abundance
m/z
329.0667
505.0993
113.0227
85.0278
71.0121
129.0175
175.0237
243.0290
271.0247
299.0197
458.1742

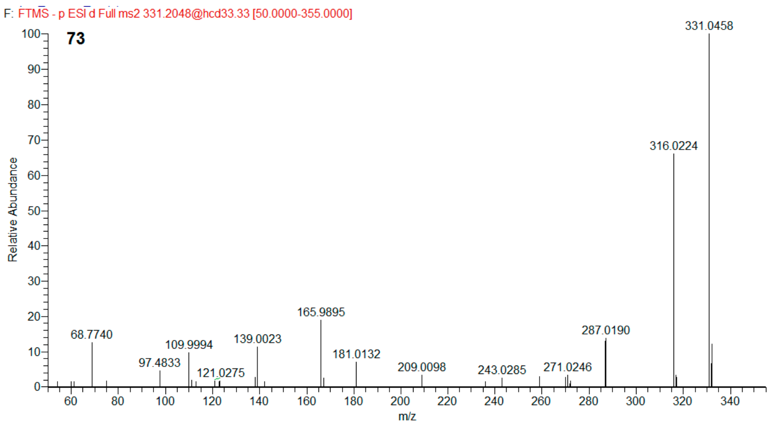
F: FTMS - p ESI d Full ms2 331.2048@hcd33.33 [50.0000-355.0000]
73
Relative Abundance
m/z
331.0458
316.0224
165.9895
68.7740
109.9994
97.4833
121.0275
139.0023
181.0132
209.0098
243.0285
271.0246
287.0190

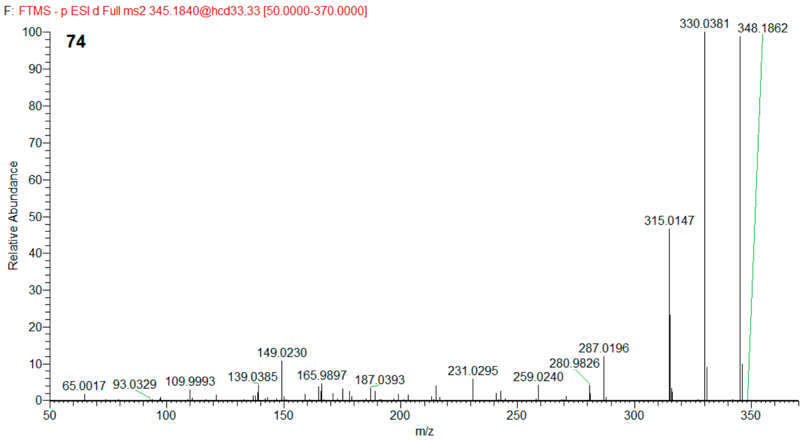
F: FTMS - p ESI d Full ms2 345.1840@hcd33.33 [50.0000-370.0000]
74
Relative Abundance
m/z
330.0381
348.1862
315.0147
149.0230
287.0196
280.9826
65.0017
93.0329
109.9993
139.0385
165.9897
187.0393
231.0295
259.0240

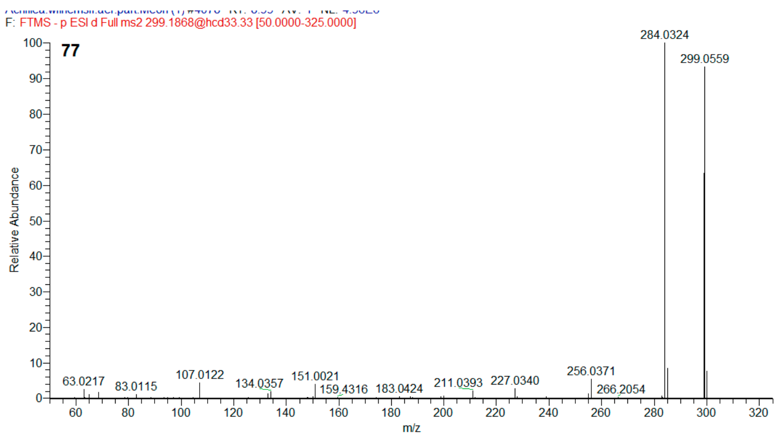
F: FTMS - p ESI d Full ms2 299.1868@hcd33.33 [50.0000-325.0000]
77
Relative Abundance
m/z
284.0324
299.0559
63.0217
83.0115
107.0122
134.0357
151.0021
159.4316
183.0424
211.0393
227.0340
256.0371
266.2054

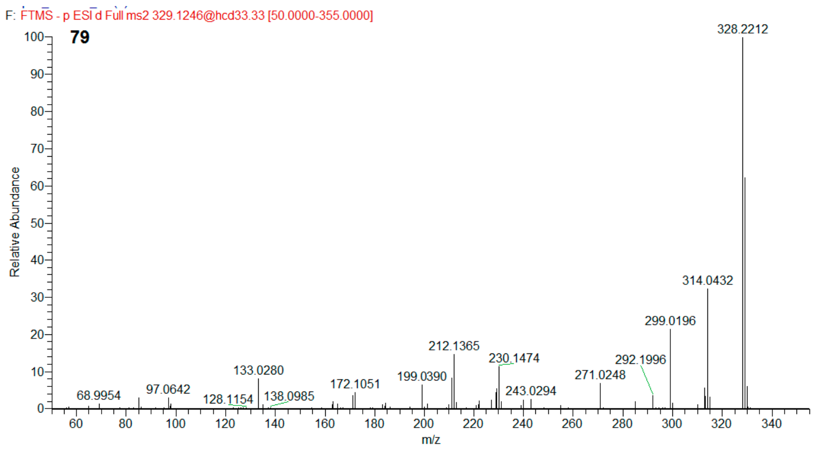
FTMS - p ESI d Full ms2 329.1246@hcd33.33 [50.0000-355.0000]
79
328.2212
314.0432
299.0196
292.1996
271.0248
243.0294
230.1474
212.1365
199.0390
172.1051
138.0985
133.0280
128.1154
97.0642
68.9954
Relative Abundance
m/z

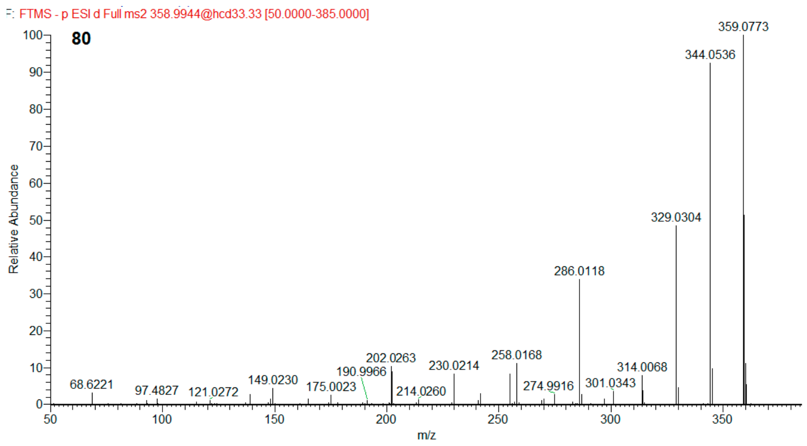
FTMS - p ESI d Full ms2 358.9944@hcd33.33 [50.0000-385.0000]
80
359.0773
344.0536
329.0304
286.0118
314.0068
301.0343
274.9916
258.0168
230.0214
214.0260
202.0263
190.9966
175.0023
149.0230
121.0272
97.4827
68.6221
Relative Abundance
m/z

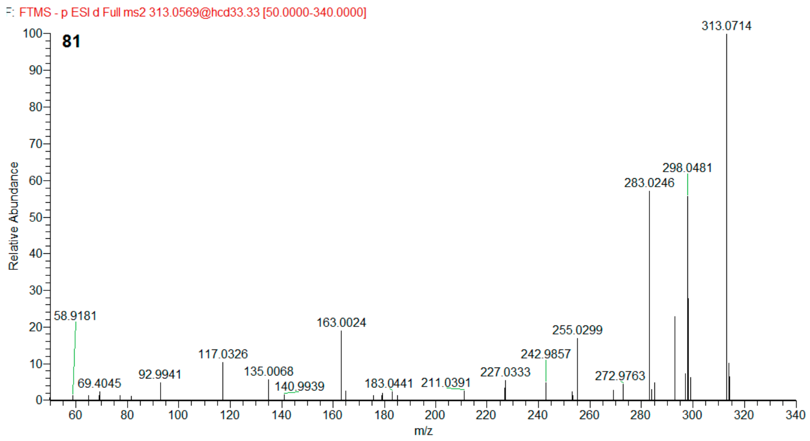
FTMS - p ESI d Full ms2 313.0569@hcd33.33 [50.0000-340.0000]
81
313.0714
298.0481
283.0246
272.9763
255.0299
242.9857
227.0333
211.0391
183.0441
163.0024
140.9939
135.0068
117.0326
92.9941
69.4045
58.9181
Relative Abundance
m/z

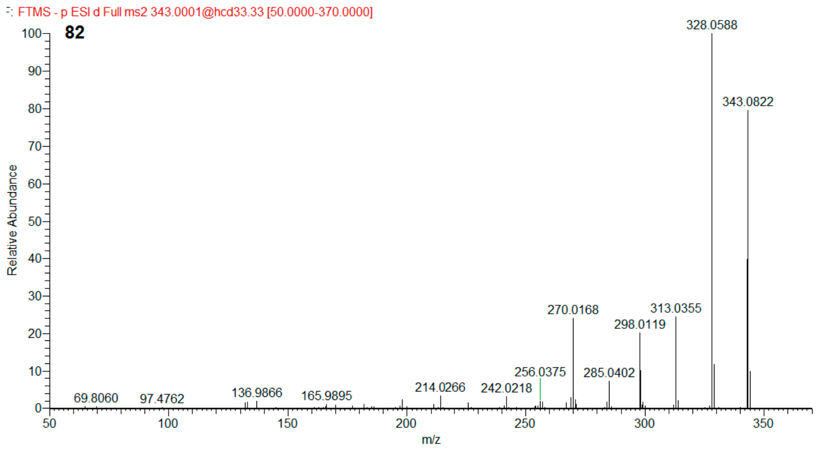
FTMS - p ESI d Full ms2 343.0001@hcd33.33 [50.0000-370.0000]
82
328.0588
343.0822
313.0355
298.0119
285.0402
270.0168
256.0375
242.0218
214.0266
165.9895
136.9866
97.4762
69.8060
Relative Abundance
m/z

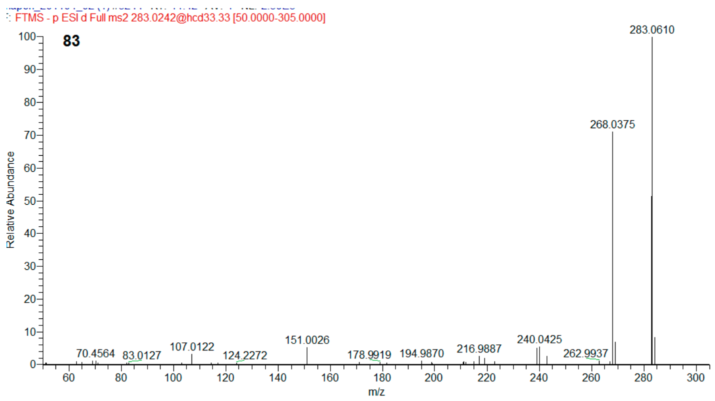

Figure S8. MS/MS spectra of 6-methoxyflavonoids (aglycones and glycosides) (for the compound numbers see Table 2).

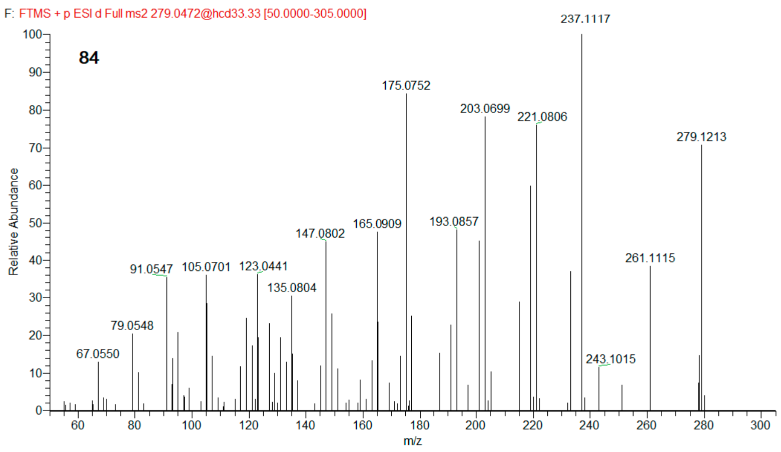
F: FTMS + p ESI d Full ms2 279.0472@hcd33.33 [50.0000-305.0000]
84
Relative Abundance
m/z
237.1117
175.0752
203.0699
221.0806
279.1213
165.0909
193.0857
147.0802
261.1115
91.0547
105.0701
123.0441
135.0804
79.0548
67.0550
243.1015

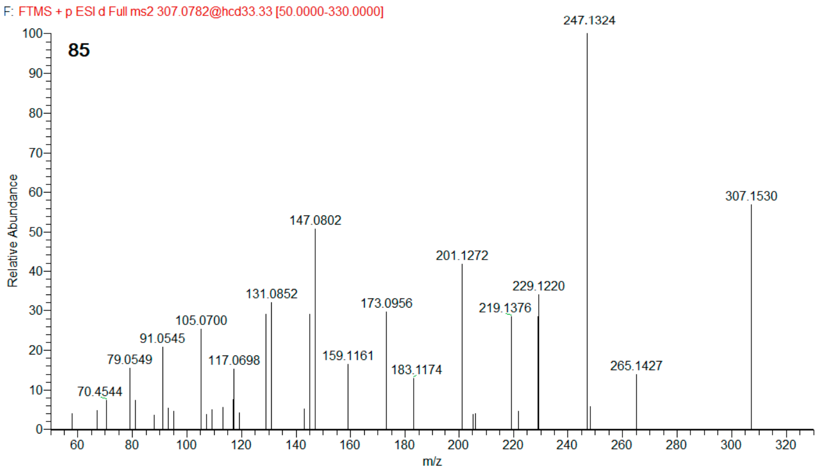
F: FTMS + p ESI d Full ms2 307.0782@hcd33.33 [50.0000-330.0000]
85
Relative Abundance
m/z
247.1324
307.1530
147.0802
201.1272
131.0852
229.1220
173.0956
219.1376
105.0700
91.0545
79.0549
117.0698
159.1161
183.1174
265.1427
70.4544

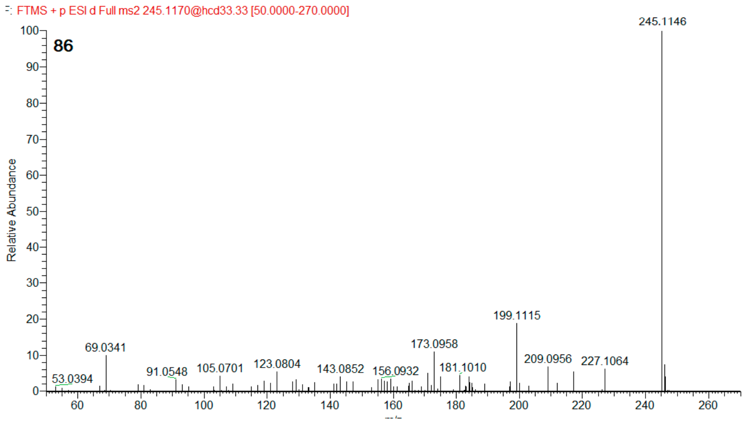
F: FTMS + p ESI d Full ms2 245.1170@hcd33.33 [50.0000-270.0000]
86
Relative Abundance
245.1146
199.1115
173.0958
69.0341
209.0956
227.1064
123.0804
181.1010
105.0701
143.0852
156.0932
91.0548
53.0394

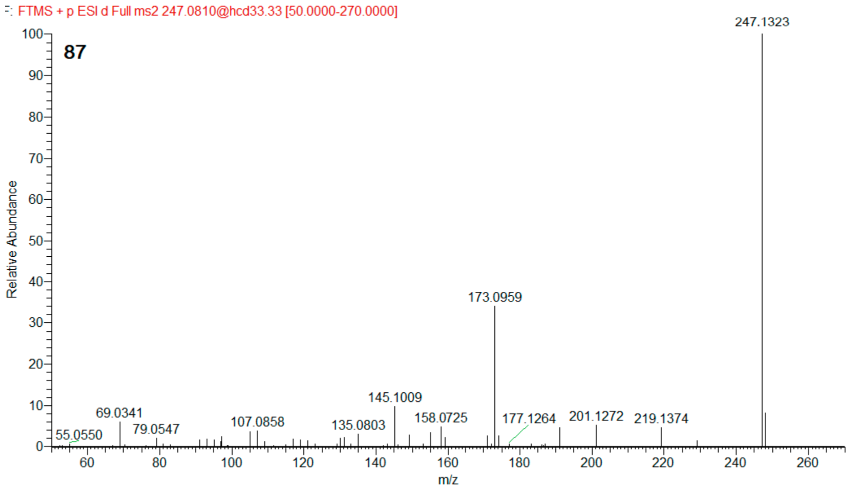
F: FTMS + p ESI d Full ms2 247.0810@hcd33.33 [50.0000-270.0000]
87
Relative Abundance
m/z
247.1323
173.0959
145.1009
69.0341
107.0858
158.0725
177.1264
201.1272
219.1374
135.0803
79.0547
55.0550

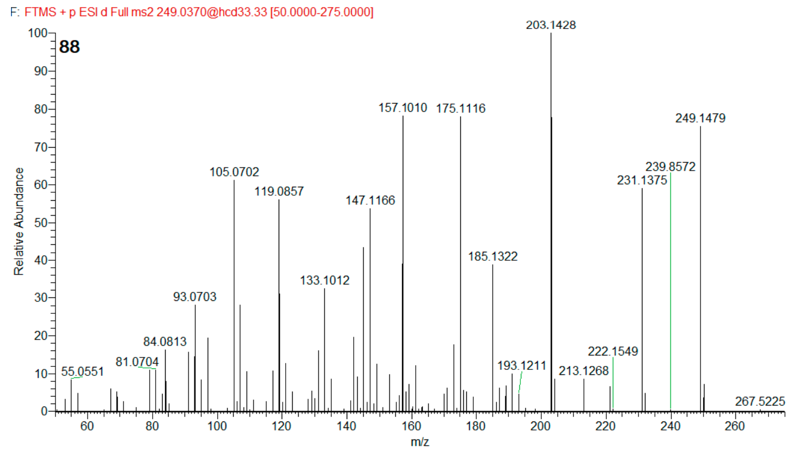
F: FTMS + p ESI d Full ms2 249.0370@hcd33.33 [50.0000-275.0000]
88
Relative Abundance
m/z
203.1428
157.1010
175.1116
249.1479
105.0702
239.8572
231.1375
119.0857
147.1166
185.1322
133.1012
93.0703
84.0813
222.1549
81.0704
193.1211
55.0551
213.1268
267.5225

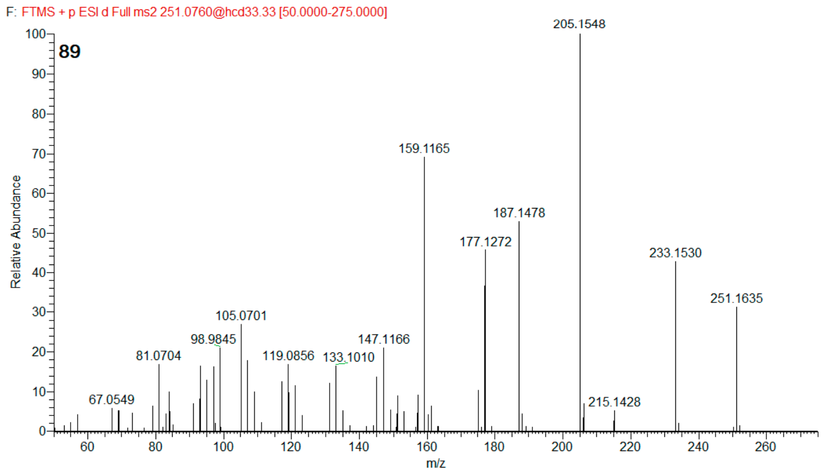
F: FTMS + p ESI d Full ms2 251.0760@hcd33.33 [50.0000-275.0000]
89
Relative Abundance
m/z
205.1548
159.1165
187.1478
177.1272
233.1530
251.1635
105.0701
98.9845
147.1166
81.0704
119.0856
133.1010
67.0549
215.1428

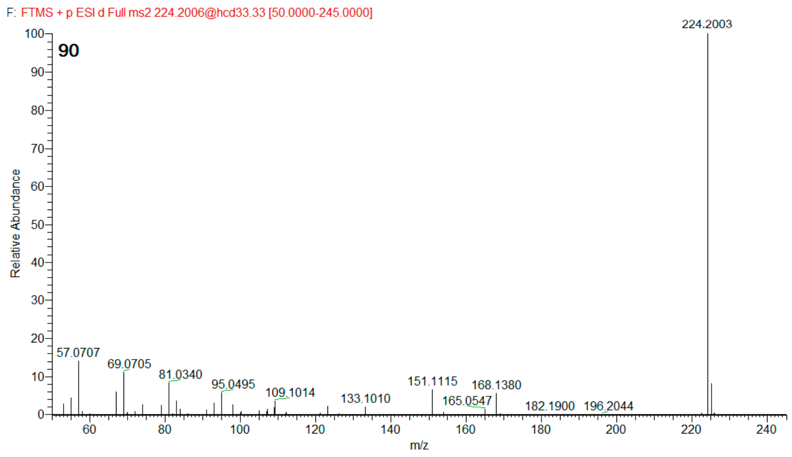
F: FTMS + p ESI d Full ms2 224.2006@hcd33.33 [50.0000-245.0000]
90
Relative Abundance
m/z
224.2003
57.0707
69.0705
81.0340
95.0495
109.1014
133.1010
151.1115
168.1380
165.0547
182.1900
196.2044

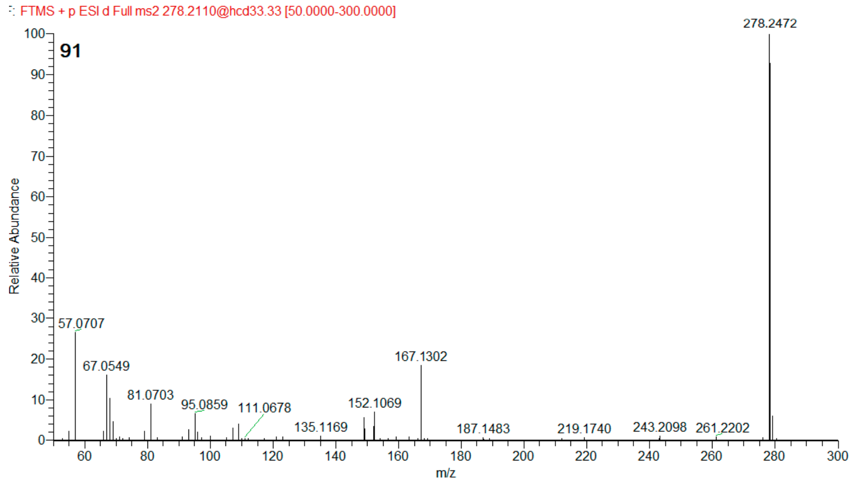
FTMS + p ESI d Full ms2 278.2110@hcd33.33 [50.0000-300.0000]
91
Relative Abundance
m/z
278.2472
57.0707
67.0549
167.1302
81.0703
95.0859
111.0678
152.1069
135.1169
187.1483
219.1740
243.2098
261.2202

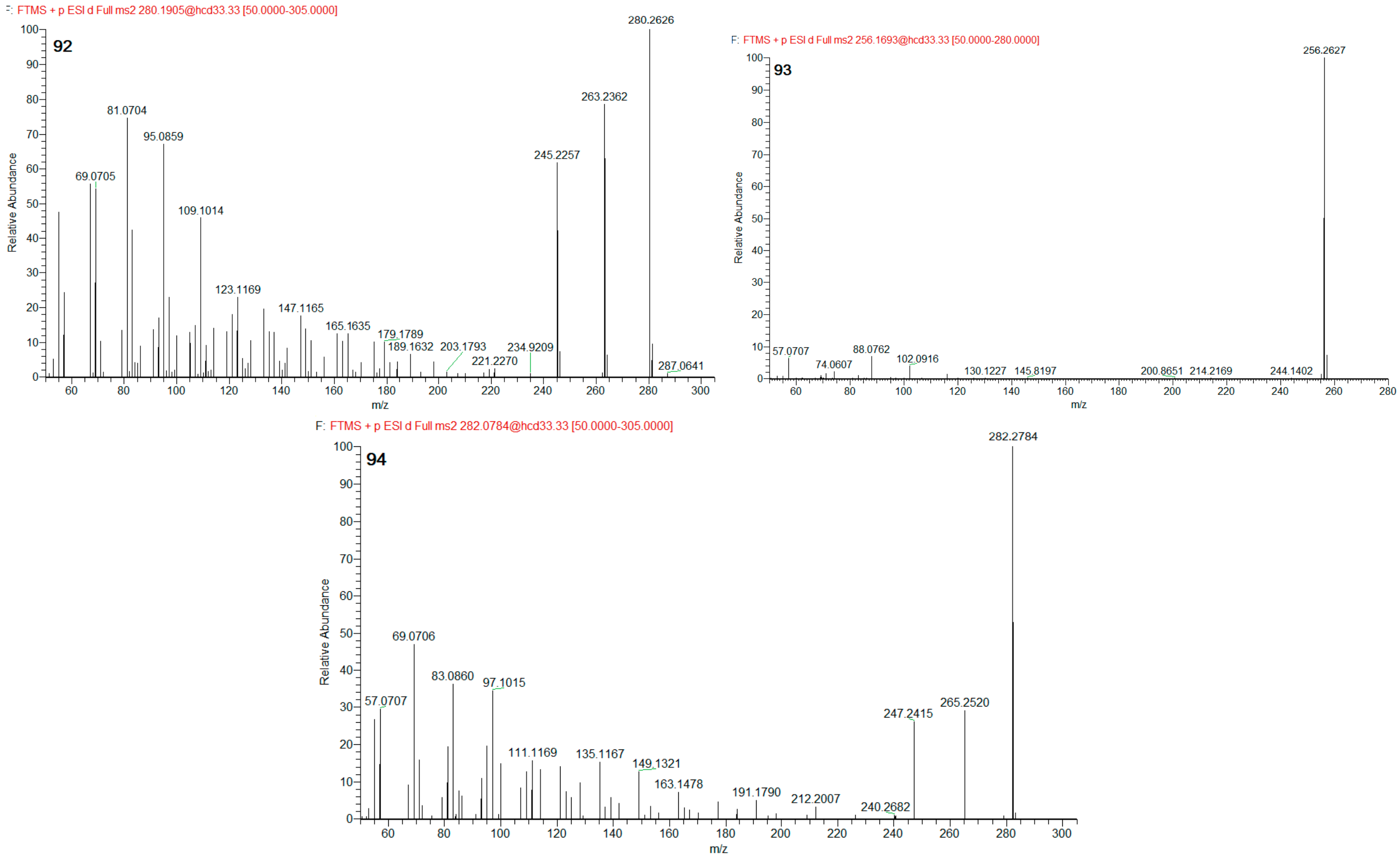

Figure S9. MS/MS spectra of sesquiterpene lactones (for the compound numbers see Table 2).
